# Chiral Fjord‐Edged Nanographenes Constructed by Allylic‐Migration‐Mediated Cyclization

**DOI:** 10.1002/anie.3554358

**Published:** 2026-09-08

**Authors:** Zhen‐Lin Qiu, Jiang‐Feng Xing, Boris Borrisov, Qing‐Song Deng, Renxiang Liu, Muhammad Imran, Ji Ma, Xinliang Feng

**Affiliations:** ^1^ Max Planck Institute of Microstructure Physics Halle Germany; ^2^ Center For Advancing Electronics Dresden (cfaed) & Faculty of Chemistry and Food Chemistry Technische Universität Dresden Dresden Germany; ^3^ State Key Laboratory for Physical Chemistry of Solid Surfaces and Department of Chemistry College of Chemistry and Chemical Engineering Xiamen University Xiamen China; ^4^ Beijing National Laboratory for Molecular Science CAS Key Laboratory for Organic Solids Institute of Chemistry Chinese Academy of Sciences Beijing China

**Keywords:** allylic migration, chiral nanographenes, cyclization, fjord‐edges

## Abstract

Fjord‐edged nanographenes (NGs) with non‐planar π‐conjugated frameworks and intrinsic chirality are attractive for nonlinear optics and chiroptical applications. However, synthetic routes to these highly twisted NGs remain elusive, largely restricted to Scholl‐type cyclization, which typically requires bulky substituents to prevent over‐dehydrogenation into conventional armchair edge structures. Here we report an allylic‐migration‐mediated cyclization strategy for the construction of fjord‐edged NGs. In this strategy, a first allylic migration drives an intramolecular cyclization that pre‐installs the fjord region. A second migration then enables a subsequent cyclization, and the final selective dehydrogenation completes the aromatization while preserving the fjord‐edged topology. DFT calculations support the essential role of each migration in enabling the corresponding cyclization. Using this strategy, we successfully synthesized fjord‐edged NGs **1** and **2**, built on chrysene and bischrysene cores, respectively. Their structures are unambiguously confirmed by single‐crystal X‐ray diffraction. Notably, **2** exhibits near‐infrared (NIR) emission extending into the NIR‐II region. Despite the absence of substituents at the fjord region, both compounds possess high racemization barriers and are readily resolved into pure enantiomers by chiral HPLC. It is remarkable that **2** exhibits an exceptional dissymmetry factor (|*g*_abs_| = 2.5 × 10^−2^), exceeding those of pristine [5]helicene and previously reported fjord‐edged NGs.

## Graphical abstract

A novel allylic‐migration‐mediated cyclization strategy enables the construction of two fjord‐edged nanographenes without steric blocking groups. The resulting non‐planar molecules exhibit remarkable chiroptical properties, with an absorption dissymmetry factor surpassing those of pristine [5]helicene and previously reported fjord‐edged nanographenes.

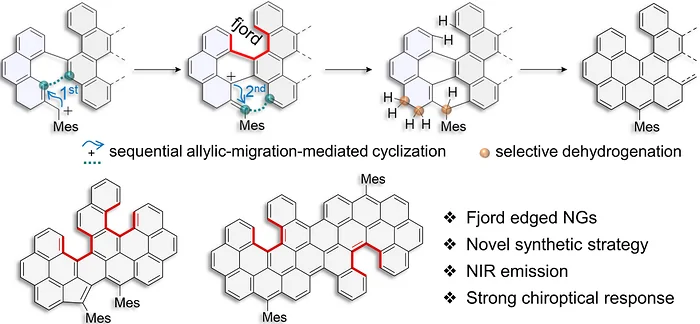

## 1 Introduction

Bottom‐up synthetic nanographenes (NGs) have garnered considerable attention owing to their tunable opto‐electronic and magnetic properties, as well as their broad potential applications in organic electronics, spintronics, bioimaging, and beyond [1, 2, 3, 4, 5]. The physicochemical properties of NGs are intrinsically governed by their structural parameters, among which molecular geometry plays a paramount role [6]. Compared to planar π‐systems, non‐planar NGs have emerged as a distinct and rapidly growing class of functional molecules, offering unique molecular geometry and fascinating chirality‐related properties [7, 8, 9, 10, 11]. Embedding non‐hexagonal rings into the sp^2^‐carbon frameworks represents a well‐established strategy for constructing non‐planar NGs, where five‐ or seven‐membered rings have been successfully incorporated to generate bowl‐ or saddle‐shaped backbones [12, 13, 14, 15, 16, 17, 18, 19]. Besides non‐hexagonal ring incorporation, congested edge topology engineering [20] provides another powerful approach for introducing non‐planarity, for example through the introduction of cove [21, 22, 23, 24] or fjord edges [25, 26, 27, 28, 29, 30, 31, 32] (Figure 1a). In particular, fjord edges, which typically correspond to [5]helicene units, arise from strong steric repulsion between overcrowded peripheral hydrogen atoms. This steric congestion enforces significant out‐of‐plane distortion without disrupting the all‐hexagonal carbon skeleton, thereby effectively suppressing intermolecular π‐π stacking in solution and imparting unique chiroptical properties. Despite these attractive features, only a few examples of fjord‐edged NGs have been reported to date, largely due to the lack of efficient and selective synthetic strategies. For instance, the widely employed Scholl reaction often suffers from poor and unpredictable regioselectivity in constructing fjord edges. In the absence of bulky substituents at the peri‐positions, uncontrolled over‐cyclization readily occurs, converting the desired fjord edges into fully fused armchair edges [33]. Consequently, sterically demanding bulky substituents are typically introduced to kinetically block the fjord positions, which substantially increases synthetic complexity (Figure 1b). These limitations highlight the need for alternative strategies with intrinsic chemoselectivity that enable the controllable and selective construction of fjord‐edged NGs, without relying on steric blocking groups.

**FIGURE 1:**
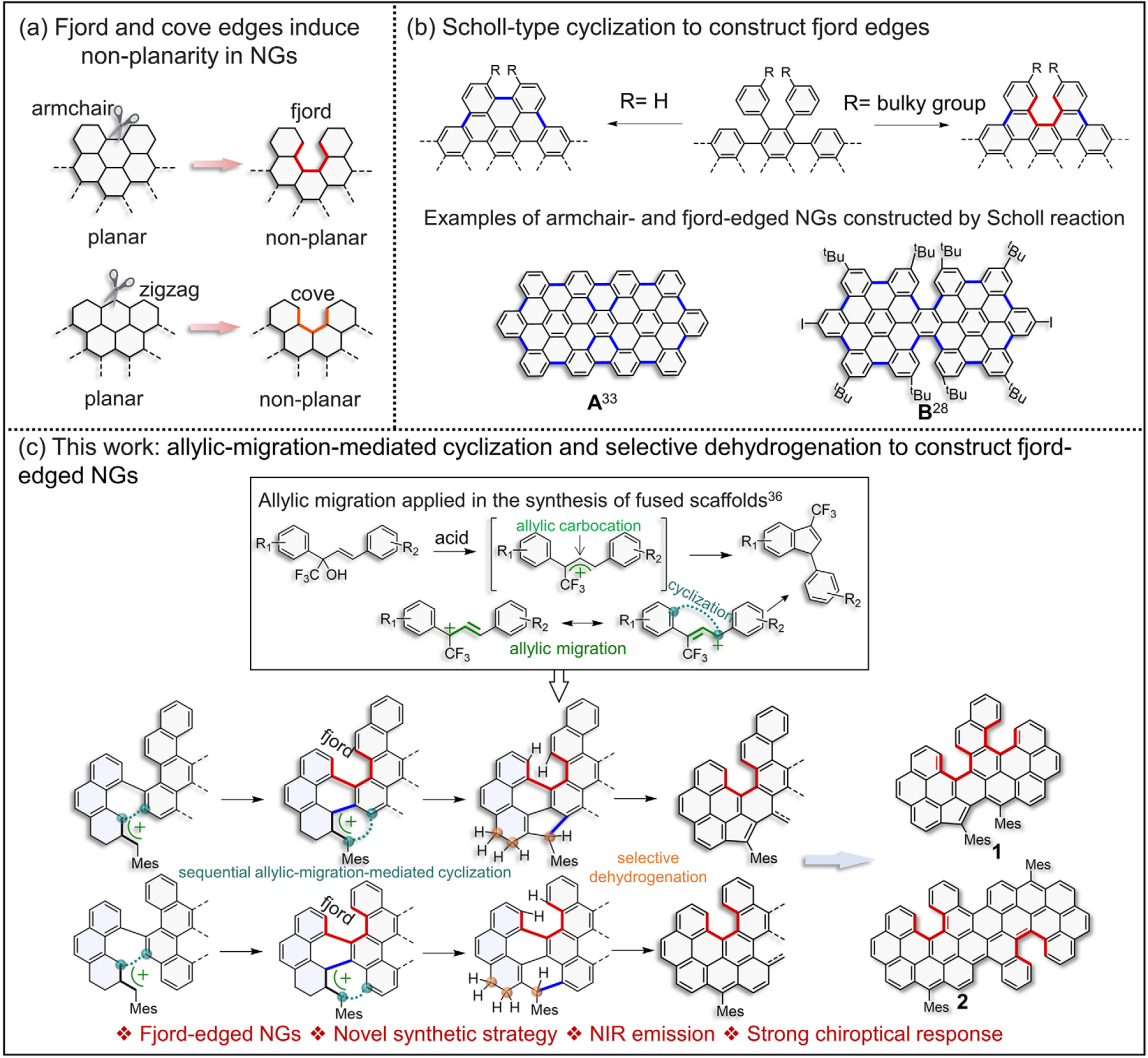
(a) Fjord and cove edges induce non‐planarity in NGs. (b) Scholl‐type cyclization to construct fjord edges. (c) This work: allylic‐migration‐mediated cyclization and selective dehydrogenation to construct fjord‐edged NGs.

Allylic migration [34], proceeding through delocalized allylic carbocation intermediates under Lewis or Brønsted acid activation, represents a well‐established strategy in organic synthesis [35]. In particular, acid‐promoted generation of an allylic cation from diaryl precursors, followed by a 1,3‐migration of the cationic center that enables subsequent intramolecular electrophilic aromatic substitution (S_E_Ar)‐based cyclization to forge a new C─C bond, offers an efficient route to fused carbocycles [36] (Figure 1c). Building on this, here we develop a novel sequential allylic‐migration‐mediated cyclization strategy for constructing fjord‐edged NGs. In this strategy, installing an unsaturated aryl unit at the bay region of an extended π‐scaffold could preserve the alkene character needed to generate the allylic cation. Upon acid promotion, a first allylic migration directs intramolecular S_E_Ar‐based cyclization to forge the first C─C bond, which establishes the fjord region and furnishes an intermediate with a pre‐installed fjord‐edge structure. Crucially, this intermediate retains its alkene character, allowing a second allylic migration to forge the second C─C bond. Because the unsaturated region is more reactive than the preformed fjord edges, the subsequent selective dehydrogenation occurs exclusively at the former, completing the aromatization while preserving the fjord topology to deliver the target fjord‐edged NGs (Figure 1c).

Using this strategy, we successfully constructed two novel fjord‐edged NGs, **1** and **2**. They feature a chrysene and a bischrysene core, respectively. Their formation relies on two key steps: a BF_3_·OEt_2_‐promoted Friedel‐Crafts alkylation and a 2,3‐dichloro‐5,6‐dicyano‐1,4‐benzoquinone (DDQ)‐mediated dehydrogenation. The former proceeds through the first allylic‐migration‐mediated cyclization, while the latter enables the second allylic‐migration‐mediated cyclization and completes the aromatization through selective dehydrogenation. DFT calculations further suggest that each allylic migration is essential for subsequent cyclization. The structures of **1** and **2** are unambiguously confirmed by NMR spectroscopy, HRMS, and single‐crystal x‐ray diffraction. Their optical and electrochemical properties were systematically investigated. Notably, **2** displays NIR emission extending into the NIR‐II region. Furthermore, despite the absence of substituents in the fjord region, both compounds exhibit high racemization barriers, thereby enabling their resolution into pure enantiomers by chiral HPLC. Remarkably, **2** exhibits a pronounced dissymmetry factor (|*g*_abs_| = 2.5 × 10^−2^) that exceeds those of pristine [5]helicene and previously reported fjord‐edged NGs.

## 2 Results and Discussion

### 2.1 Synthesis and Structure Determination of 1 and 2

The synthesis of fjord‐edged NGs **1** and **2**, featuring chrysene and bischrysene cores, is outlined in Scheme 1. First, 5‐(4,4,5,5‐tetramethyl‐1,3,2‐dioxaborolan‐2‐yl)‐1,2‐dihydrophenanthrene‐3‐carbaldehyde (**4**) bearing the requisite unsaturated aryl unit was successfully obtained in 95% yield from 4‐bromo‐1,2‐dihydrophenanthrene‐3‐carbaldehyde (**3**) via Miyaura borylation, during which a 1, 4‐migration occurred. The structure of **4** was unambiguously confirmed by NMR spectroscopy and single‐crystal x‐ray analysis (Figure S1). Subsequently, Suzuki coupling of **4** with 5,11‐dibromochrysene (**5**, synthesized as detailed in the Supporting Information) afforded 5,5'‐(chrysene‐4,11‐diyl)bis(1,2‐dihydrophenanthrene‐3‐carbaldehyde) (**6**) in 30% yield, in which a 1,4‐migration at the bay position of the chrysene core occurred to give the unsymmetrical product, as confirmed by single‐crystal x‐ray analysis (Figure S1). Treatment of **6** with mesitylmagnesium bromide gave a diol intermediate, which upon BF_3_·OEt_2_‐promoted Friedel‐Crafts alkylation underwent the first allylic carbocation migration and ensuing intramolecular cyclization, affording the proposed intermediate **7** with the fjord region pre‐installed. After quick purification through a short pad of silica gel, the formation of **7** was confirmed by high‐resolution matrix‐assisted laser desorption/ionization‐time‐of‐flight (MALDI‐TOF) mass spectrometry (Figure S2a). Owing to its limited stability (Figure S2b), **7** was used directly in the next step. Subsequent treatment of **7** with DDQ in dry toluene at 50°C under argon promoted the second allylic migration to forge the second C─C bond, followed by selective dehydrogenation that completed the aromatization, delivering **1** as a green solid in 23% yield over three steps from **6**. Compound **2** was prepared by a similar route. Suzuki coupling of **4** with the prepared 9,18‐dibromobenzo[*a*]dinaphtho[2,1,8‐*cde*:1',2',3',4'‐*ghi*]perylene (**8**) afforded **9** in 35% yield, with its structure confirmed by single‐crystal x‐ray analysis (Figure S1). Subsequent Grignard reaction and BF_3_·OEt_2_‐promoted Friedel‐Crafts alkylation of **9** proceeded through the first allylic carbocation migration and cyclization to give the proposed intermediate **10**, which was similarly confirmed by high‐resolution mass spectrometry and used directly owing to its limited stability (Figure S2c,d). Treatment of **10** with DDQ in dry toluene at 50°C under argon then prompted the second allylic migration and subsequent selective dehydrogenation to furnish **2** as a dark‐green solid in 24% yield over three steps from **9**. To further corroborate the structures of the key intermediates **7** and **10**, a more stable single‐sided analogue **11** was prepared under identical conditions (Figure S3). Benefiting from its enhanced stability, **11** was fully characterized, and its structure was unambiguously confirmed by ^1^H NMR together with 2D COSY and NOESY experiments, in which all proton resonances were assigned (Figures S4, S5). As **11** is formed through the same reaction pathway, its unambiguous structural determination supports the proposed structures of **7** and **10**.

**SCHEME 1:**
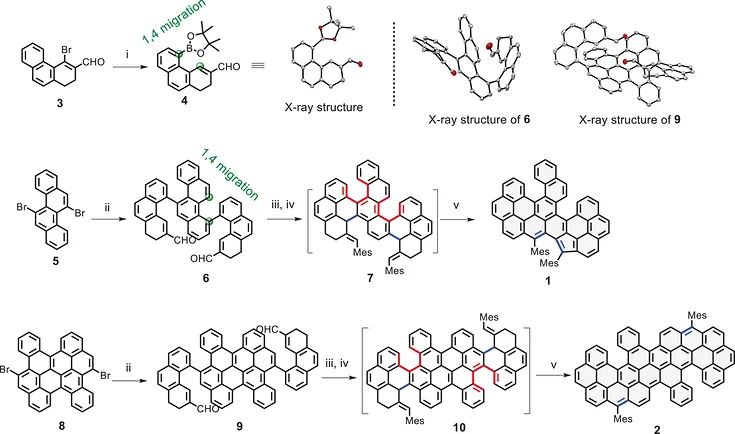
Synthetic routes toward **1** and **2**. Reagents and conditions: (i) Bis(pinacolato)diboron, Pd(dppf)Cl_2_, KOAc, 1,4‐dioxane, 90°C, 12 h; (ii) **4**, Pd(PPh_3_)_4_, CsF, 1,2‐Dimethoxyethane, 85°C, 12 h; (iii) MesMgBr, tetrahydrofuran (THF), rt, 2 h; (iv) BF_3_·OEt_2_, dichloromethane (DCM), rt, 4 h; (v) 2,3‐Dichloro‐5,6‐dicyano‐1,4‐benzoquinone, toluene, 30 min.

The obtained compounds, **1** and **2**, were initially analyzed using high‐resolution mass spectrometry (Figure S6). Both compounds exhibited a single peak, confirming their defined molecular composition, with isotopic distribution patterns in good agreement with the calculated values. NMR analysis was subsequently conducted to further validate their chemical structures (Figures S7–S8). Furthermore, the structures of **1** and **2** were unambiguously confirmed by single‐crystal x‐ray analysis (Figure 2). Crystals of **1** and **2** suitable for x‐ray diffraction were each obtained by slow diffusion of methanol vapor into their respective chlorobenzene solutions at room temperature. As shown in Figure 2, compound **1** adopts a non‐planar geometry with a curvature depth of 1.6 Å owing to the presence of two fjord edges, with torsion angles of 42.2° and 31.0° (Figure 2a,b). Both values exceed that of pristine [5]helicene (27.8°), likely reflecting the additional steric strain imposed by the two adjacent fjord regions. Owing to the unsymmetric molecular backbone, the two [5]helicene units are inequivalent, rendering the (*P/M*) and (*M*/*P*) forms a genuine pair of enantiomers rather than a meso structure. Consistent with this, the packing view along the *c*‐axis reveals that **1** exists as a pair of enantiomers ((*P*/*M*) and (*M*/*P*) in a 1:1 ratio, which assemble into a quadrilateral arrangement through intermolecular C‐H···π interactions (Figure 2c). As for **2**, it adopts a saddle‐shaped conformation, with the upper segment measuring 17.8 Å in width and 2.4 Å in depth. The torsion angles of the two fjord regions are 27.1° and 23.9°, smaller than those in **1**, indicating less steric strain at the fjord edges of **2** (Figure 2d,e). This saddle shape arises from the combination of the two fjord edges and the two cove edges interposed between them. Because each fjord and its neighboring cove region shares two benzene rings, they are forced to adopt opposite helical configurations, which drives the shared and adjacent rings to tilt alternately upward and downward along the backbone. This up‐and‐down alternation of the benzene rings produces the saddle‐shaped, negatively curved geometry. The packing structure shows that **2** exists as a pair of enantiomers ((*P*/*P*) and (*M*/*M*)) in a ratio of 1:1 in the unit cell, featuring partially overlapping π‐π interactions with a stacking distance of approximately 3.4 Å (Figure 2f).

**FIGURE 2:**
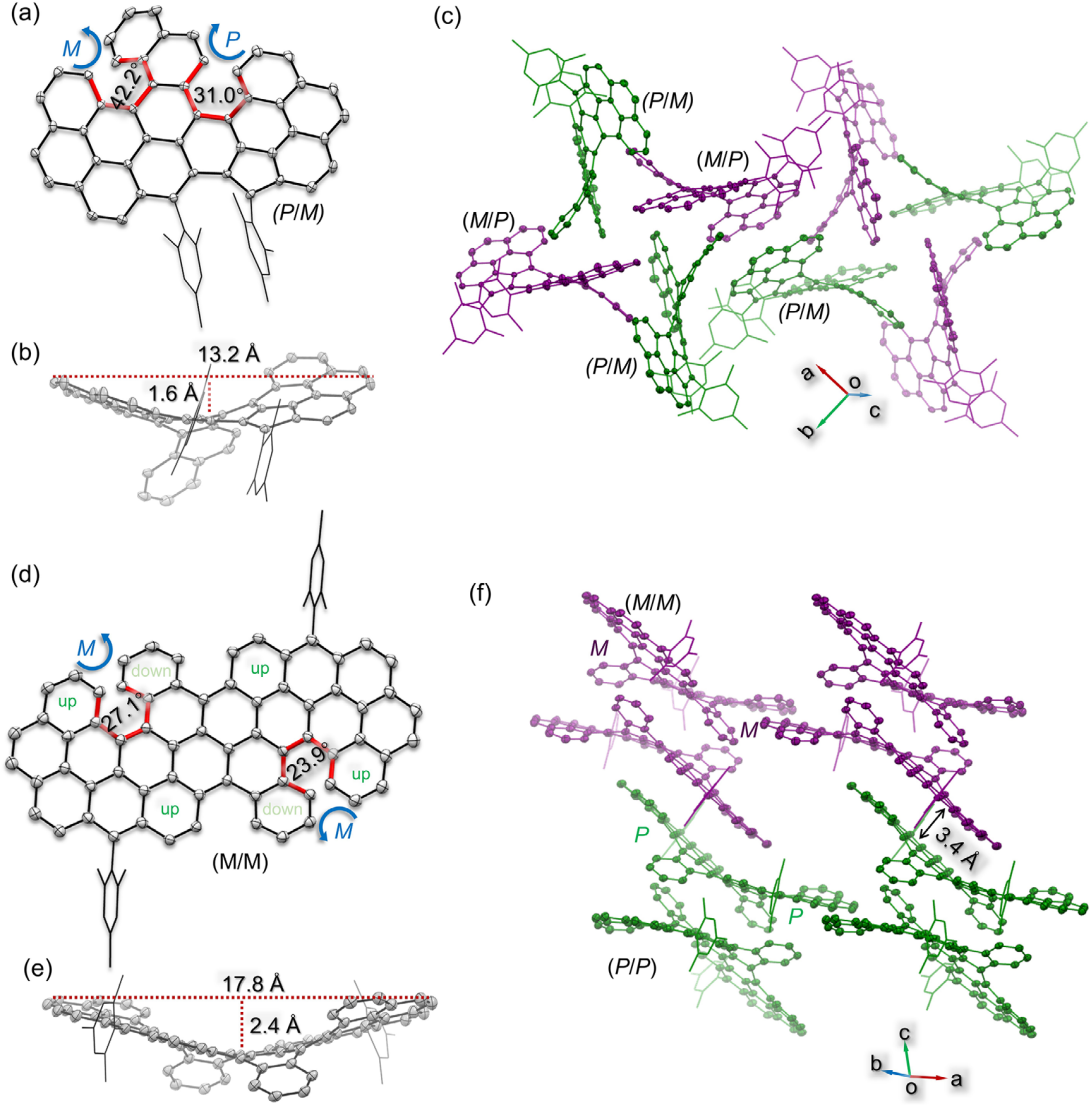
X‐ray crystallographic structures of **1** and **2**. (a) top view, (b) side view, and (c) molecular packing of **1**; (d) top view, (e) side view, and (f) molecular packing of **2**. The thermal ellipsoids are set at a probability level of 30%. The hydrogen atoms are omitted for clarity.

### 2.2 Mechanistic Investigations

To elucidate the reaction mechanism, DFT calculations were performed at the B3LYP‐D3(BJ)/def2‐TZVP level of theory on compound **1** (Figure 3). Consistent with the two‐step strategy described above, the construction of **1** involves two key steps, each enabled by a cationic allylic migration that relocates the carbocation to the position required for the ensuing cyclization. The first migration occurs during the BF_3_·OEt_2_‐promoted Friedel‐Crafts alkylation, which builds the precursor **7** with preinstalled fjord regions. The second occurs upon treatment with DDQ, in which a further migration enables the final cyclization, followed by dehydrogenation by DDQ that aromatizes the precursor into the fully conjugated product **1**.

**FIGURE 3:**
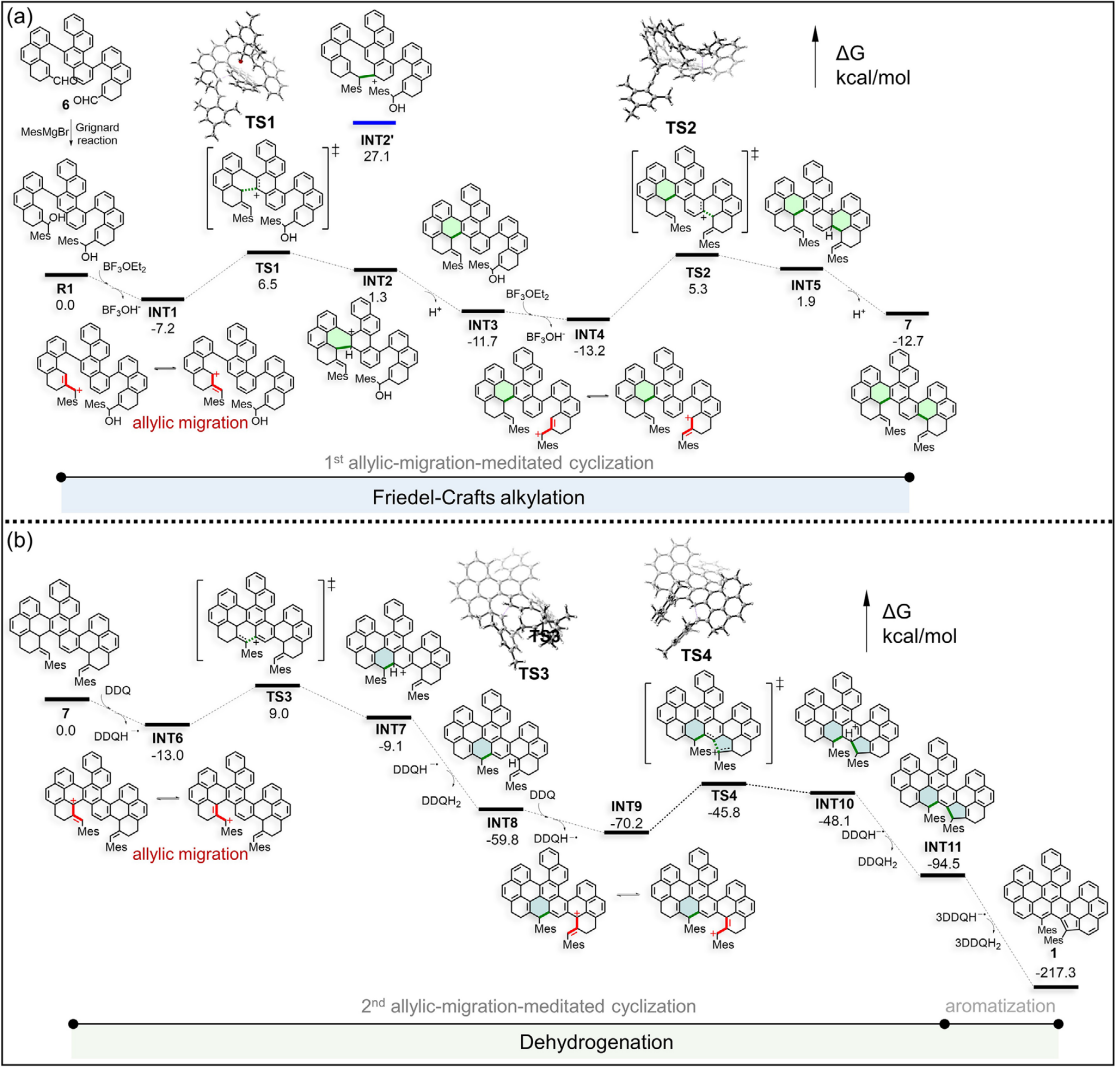
Gibbs free energy profiles calculated for **R1** → **7** (a) and **7** → **1** (b). Gibbs free energy is given in kcal/mol. The optimized structures of **TS1**, **TS2**, **TS3**, and **TS4** are shown.

In the Friedel‐Crafts alkylation step (Figure 3a), BF_3_·OEt_2_ coordinates to the hydroxyl group of the diol intermediate **R1** (formed from **6** by Grignard addition), facilitating ionization of the C─O bond to generate the allylic carbocation intermediate **INT1** with an exergonicity of 7.2 kcal/mol. From **INT1**, the carbocation undergoes allylic migration and subsequent intramolecular S_E_Ar‐based cyclization via the transition state **TS1** with a barrier of 13.7 kcal/mol to afford **INT2**. An alternative pathway in which S_E_Ar occurs directly without prior allylic migration was also evaluated; however, the barrier from **INT1** to **INT2'** is 34.3 kcal/mol, significantly higher than that of the migration pathway, indicating that this route is energetically prohibitive. The pronounced energy difference between the two pathways underscores the essential role of allylic migration in enabling the cyclization. Deprotonation of **INT2** then yields **INT3** with an exergonicity of 13.0 kcal/mol. The same sequence operates on the other side of the molecule (**INT3** → **INT4** → **TS2** → **INT5** →** 7**), completing the allylic migration on both sides and furnishing the intermediate **7** with a pre‐installed fjord‐edge region.

In the dehydrogenation step (Figure 3b), DDQ first abstracts a hydrogen atom from the sp^3^ carbon of **7**, generating the carbocation intermediate **INT6** with an exergonicity of 13.0 kcal/mol. This carbocation then undergoes the second allylic migration, which enables the intramolecular S_E_Ar via **TS3** (barrier of 22.0 kcal/mol) to afford **INT7**. Subsequent deprotonation yields **INT8** with an exergonicity of 50.7 kcal/mol. The same sequence operates on the other side of the molecule (**INT8** → **INT9** → **TS4** → **INT10** → **INT11**), furnishing **INT11** with an exergonicity of 34.7 kcal/mol. The large exergonicity of both the Friedel‐Crafts alkylation and the dehydrogenation steps provides a strong thermodynamic driving force for the allylic migrations. Further selective dehydrogenation of the unsaturated C─H bonds in **INT11** completes the aromatization, delivering **1** with its fjord‐edge topology preserved.

### 2.3 Optical and Electrochemical Properties

Benefiting from their non‐planar geometries that suppress intermolecular π‐π aggregation, **1** and **2** show good solubility in common organic solvents such as DCM, toluene, and THF, even though each molecule bears only two mesityl groups. Their optical properties were investigated by UV‐vis absorption and fluorescence spectroscopy in degassed toluene (Figure 4a). Compound **1** displays broad absorption bands with a maximum absorption peak (*λ*_max_) at 642 nm (*ε* = 5.5 × 10^4^ M^−1^ cm^−1^), while **2** exhibits red‐shifted absorption relative to **1** with *λ*_max_ at 733 nm (*ε* = 9.2 × 10^4^ M^−1^ cm^−1^), owing to the extended π‐conjugation. The optical band gaps are estimated to be 1.63 and 1.60 eV for **1** and **2**, respectively. To further elucidate the experimental results, computational investigations of nanographenes **1** and **2** were performed at the TD‐B3LYP‐D3(BJ)/6‐31G(d) level of theory, utilizing the polarizable continuum model (PCM) to implicitly account for toluene solvation (Figure S9). Based on these calculations, the corresponding absorption peaks for both **1** and **2** are assigned to the S_0_ → S_1_* transition. Additionally, frontier molecular orbital (FMO) analysis reveals that in **1,** the transition involves contributions from the HOMO, LUMO, and HOMO‐1, whereas in **2**, it arises exclusively from a HOMO‐LUMO excitation (Figure 4c).

**FIGURE 4:**
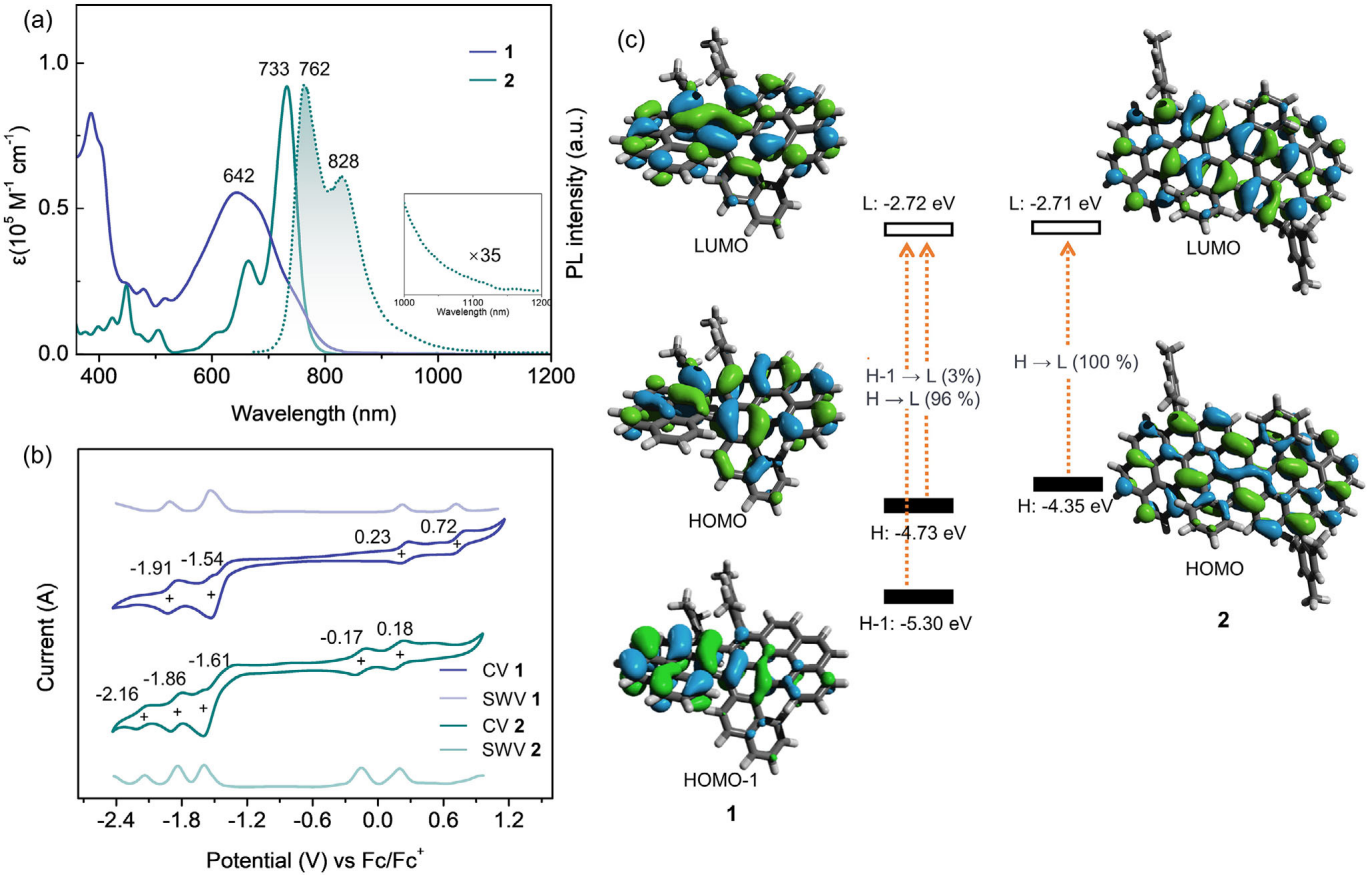
(a) Absorption (solid line) and emission (dashed line) spectra of **1** and **2** in toluene. The inset shows the enlarged emission spectrum of **2** in the region above 1000 nm (×35). (b) Cyclic voltammetry (CV) and square wave voltammetry (SWV) of **1** and **2** in a solution of Bu_4_NPF_6_ (0.1 M) in DCM with a scan rate of 100 mV s^−1^ at room temperature. (c) Molecular orbitals and the energy diagrams of **1** and **2** calculated at B3LYP‐D3(BJ)/6‐31G(d) level of theory with an implicit solvent toluene (PCM).

Notably, **2** shows NIR emission with two principal emission bands at 762 and 828 nm, followed by a long tail extending into the NIR‐II region. Fluorescence lifetime measurements yielded a value of 8.1 ns (Figure S10), and the absolute photoluminescence quantum yield (PLQY) of **2** was determined to be 5% using an integrating sphere. This low quantum yield may be attributed to the energy gap law, whereby the small emission energy gap of NIR emitters promotes non‐radiative decay. Moreover, the electrochemical redox behavior of **1** and **2** were examined by cyclic voltammetry (CV) and square wave voltammetry (SWV) in DCM containing 0.1 M Bu_4_NPF_6_ as supporting electrolyte (Figure 4b). Compound **1** exhibits two reversible oxidation waves with half‐wave potentials E^ox^_1/2_ of 0.23 and 0.72 V (vs. Fc/Fc^+^) and two quasi‐reversible reduction waves with E^red^_1/2_ of ‐1.54 and ‐1.91 V (vs. Fc/Fc^+^). Compound **2** displays two reversible oxidation waves with E^ox^_1/2_ of ‐0.17 and 0.18 V (vs. Fc/Fc^+^) and three quasi‐reversible reduction waves with E^red^_1/2_ of ‐1.61, ‐1.86, and ‐2.16 V (vs. Fc/Fc^+^). These half‐wave potentials are in good agreement with square wave voltammetry (SWV) measurements. The highest occupied molecular orbital (HOMO) and lowest unoccupied molecular orbital (LUMO) energy levels were estimated to be −5.03 and −3.26 eV for **1** and −4.63 and −3.19 eV for **2**, corresponding to electrochemical energy gaps of 1.77 and 1.44 eV, respectively, in good agreement with the trend obtained from DFT calculations.

To further investigate the influence of the fjord edge topology on the physicochemical properties of these NGs, compound **2** was subjected to a Scholl reaction to afford the fully fused product **2F** (Figure S11). Despite its more extended π‐conjugation, **2F** displays blue‐shifted absorption and emission relative to **2** (Figure S11c). In addition, cyclic voltammetry reveals that the oxidation potentials of **2F** are anodically shifted relative to those of **2** measured under identical conditions (Figure S11d). Together, these results demonstrate the pronounced influence of the fjord edge topology on both the optical and electrochemical properties of NGs.

### 2.4 Chiral Optical Properties

Considering the presence of [5]helicene units in compounds **1** and **2**, DFT calculations of their enantiomerization pathways were performed at the B3LYP/6‐31G(d) level to evaluate their configurational stability. For both compounds, racemization proceeds through sequential inversion of the helicene units and is governed by the inversion of [5]helicenes, with barriers of 38.2 and 34.4 kcal/mol for **1** and **2**, respectively. These high barriers indicate robust configurational stability at room temperature, even in the absence of substituents at the fjord region and thus enabled their successful enantiomeric separation by chiral high‐performance liquid chromatography (HPLC) using a (*R,R*)‐Whelk‐O 15 µm Kromasil column, affording pure enantiomers **1**‐*PM*/**1**‐*MP* and **2**‐*PP*/**2**‐*MM*, respectively (Figure S12). The chiroptical properties of the enantiomers were investigated by circular dichroism (CD) spectroscopy (Figure 5b–e), with absolute configurations assigned based on the correlation between experimental CD spectra and TD‐DFT results (Figure S13). As shown in Figure 5b, 1‐P*M* and **1**‐*MP* display mirror‐image Cotton effects across the UV‐visible region, with |Δ*ε*|_max_ = 224 M^–^^1^·cm^–^^1^ at 309 nm and |Δ*ε*|_max_ = 61 M^–^^1^·cm^–^^1^ at 538 nm. Absorption dissymmetry factor analysis revealed a maximum |g_abs_| of 3.1 × 10^–^^3^ at 538 nm for **1** (Figure 5c). The enantiomeric pair **2**‐*PP* and **2**‐*MM* likewise exhibits strong mirror‐image Cotton effects, |Δε|_max_ = 197 M^–^^1^·cm^–^^1^ at 500 nm and |Δε|_max_ = 67 M^–^^1^·cm^–^^1^ at 722 nm. Notably, **2** displays a maximum |*g*_abs_| of 2.5 × 10^−2^ at 500 nm (Figure 5d), superior to those pristine [5]helicene and previously reported fjord‐edged NGs (|*g*_abs_| ranging from 4.7× 10^−4^ to 1.7× 10^−2^) [25, 26, 31, 37, 38] (Figure S14, Table S1). Circularly polarized luminescence (CPL) measurements of **2** revealed mirror‐image responses from the enantiomers, with a CPL signal at 815 nm and a |*g*_lum_| of 7.5 × 10^−4^ (Figure S15).

**FIGURE 5:**
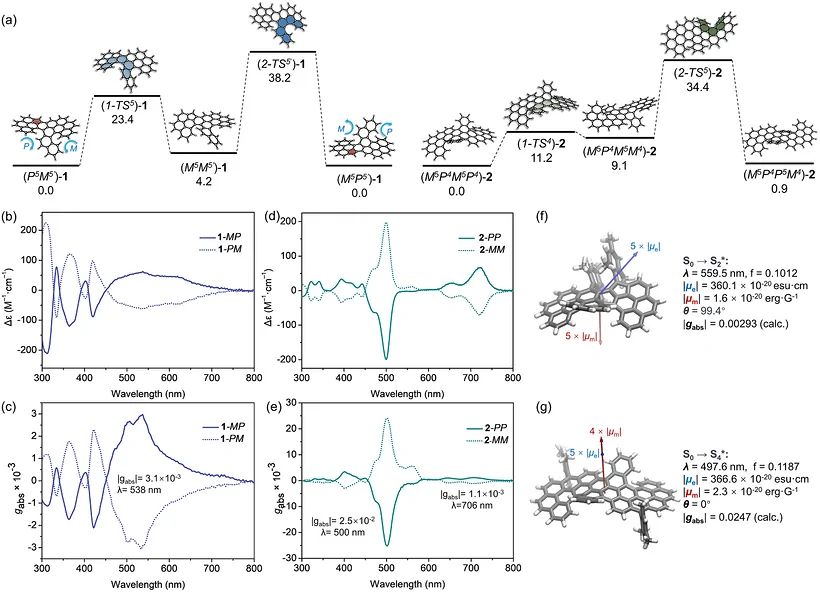
(a) Theoretical isomerization process of **1** and **2** in units of kcal/mol simulated at B3LYP/6‐31G(d) level. (b, c) Experimental CD spectra of the pure enantiomers of **1** (*c* = 3.2 × 10^−6^ M) and **2** (*c* = 1.6 × 10^−5^ M), and (d, e) the corresponding *g*_abs_ factors in anhydrous toluene. (f) Transition dipole moments of **1** for the S_0_ → S_2_* transition (g) Transition dipole moments of **2** for the S_0 _→ S_4_* transition. The electric transition dipole moments (*µ*_e_) are shown in blue, while the magnetic transition dipole moments (*µm*) are shown in red. The lengths of the transition dipole moment vectors are scaled for clarity.

To gain insight into the unique chiroptical properties of **1** and **2**, TD‐DFT calculations were performed with the same level of theory as those for UV‐Vis calculations. The CD response with the maximum *g*_abs_ is assigned to the S_0_ → S_2_* transition for **1** and S_0_ → S_4_* transition for **2**, with the corresponding electric transition dipole moments (*µ*_e_), magnetic transition dipole moments (*µm*), and the angle between them (*θ*) shown in Figure 5f,g. Both compounds exhibit similar magnitudes of |*µm*| and |*µ*_e_|, such that the *g*‐factor is critically governed by the value of *θ* according to the simplified formula *g* = 4 cos(*θ*) × |*µm*|/|*µ*_e_|. Compound **2** features a parallel alignment of *µ*_e_ and *µm* (*θ* = 0°), whereas the two moments are nearly perpendicular in **1** (*θ* = 99.4°). The parallel alignment in **2** is a consequence of its *C_2_* symmetry, which constrains the electric and magnetic transition dipole moments to lie parallel and thereby maximizes cos(*θ*) [31, 39]. Compound **1** lacks such symmetry, and its two moments remain nearly perpendicular. As the dissymmetry factor scales with cos(*θ*), this difference accounts for the nearly order‐of‐magnitude enhancement in |*g*_abs_| of **2** relative to **1**, as well as for its |*g*_abs_| being superior to those of previously reported fjord‐edged NGs.

## 3 Conclusion

In summary, we have developed an allylic‐migration‐mediated cyclization strategy for the construction of fjord‐edged NGs. Using this approach, we synthesized two novel fjord‐edged NGs, **1** and **2**, built on chrysene and bischrysene cores. Their formation involves two sequential allylic‐migration‐mediated cyclizations and a final aromatization, realized through a BF_3_·OEt_2_‐promoted Friedel‐Crafts alkylation and a subsequent DDQ‐mediated dehydrogenation. DFT calculations show that each migration is essential for the corresponding cyclization, with the direct pathway being energetically prohibitive. Both NGs adopt non‐planar structures arising from the introduction of fjord edges. Notably, compound **2** exhibits NIR emission with a tail extending into the NIR‐II region. Despite the absence of substituents at the fjord region, both compounds possess high racemization barriers and were resolved into configurationally stable enantiomers by chiral HPLC. Remarkably, **2** exhibits a pronounced chiroptical response that exceeds those of pristine [5]helicene and previously reported fjord‐edged NGs. This work establishes allylic‐migration‐mediated cyclization as an effective synthetic approach for constructing fjord‐edged NGs, expanding the synthetic toolbox for nanographene construction and offering new insights into the design of chiral π‐conjugated carbon nanostructures.

## Author Contributions

**Zhen‐Lin Qiu**: investigation, writing – original draft, conceptualization, validation, writing – review and editing, formal analysis, data curation. **Jiang‐Feng Xing**: methodology, investigation. **Boris Borrisov**: investigation, software. **Qing‐Song Deng**: investigation. **Renxiang Liu**: methodology. **Muhammad Imran**: data curation. **Ji Ma**: funding acquisition, writing – review and editing, supervision, conceptualization. **Xinliang Feng**: conceptualization, supervision, resources, writing – review and editing, funding acquisition, project administration.

## Conflicts of Interest

The authors declare no conflicts of interest.

## Supporting information



The data that support the findings of this study are available in Supplementary Information.**Supporting File**: anie74707‐sup‐0001‐SuppMat.docx.

## Data Availability

The data that support the findings of this study are openly available in the Cambridge Crystallographic Data Centre at https://www.ccdc.cam.ac.uk/, reference number 2541397–2541401.
