# Supplementary material for: Chiral Fjord‐Edged Nanographenes Constructed by Allylic‐Migration‐Mediated Cyclization
Source: Angew Chem Int Ed Engl. 2026 Sep 8;65(44):e3554358. doi: 10.1002/anie.3554358 (PMC13703807; doi:10.1002/anie.3554358)
Supplement: Supplementary file 1 [file ANIE-65-e3554358-s001.docx]

**Supporting information**

**Chiral Fjord-Edged Nanographenes Constructed by Allylic-Migration-Mediated Cyclization**

Zhen-Lin Qiu^1,2^, Jiang-Feng Xing^3^, Boris Borrisov^2^, Qing-Song Deng^3^, Renxiang Liu^1,2^, Muhammad Imran^1,2^, Ji Ma^4^* and Xinliang Feng^1,2^*

^1^ Max Planck Institute of Microstructure Physics, Halle, Germany. ^2^ Center for Advancing Electronics Dresden (cfaed) & Faculty of Chemistry and Food Chemistry, Technische Universität Dresden, Dresden, Germany. ^3^ State Key Laboratory for Physical Chemistry of Solid Surfaces, and Department of Chemistry, College of Chemistry and Chemical Engineering, Xiamen University, Xiamen, China. ^4^ Beijing National Laboratory for Molecular Science, CAS Key Laboratory for Organic Solids, Institute of Chemistry, Chinese Academy of Sciences, Beijing, China.

**S1. Supplementary Figures and Tables**

**Figure S1**. The crystal structure of **9** (a), **5** (b) and **6** (c). The thermal ellipsoids are set at a probability level of 30%.

**Figure S2**. (a) HR-MALDI-TOF mass spectra of **7**. (b) Time-dependent HR-MALDI-TOF mass spectra of **7** upon exposure to air. (c) HR-MALDI-TOF mass spectra of **7**. (d) Time-dependent HR-MALDI-TOF mass spectra of **10** upon exposure to air.

**Figure S3**. Synthesis and characterization of **11**. (a) Synthetic of **11**. (b) HR-MALDI-TOF mass spectra of **11**. (c) ^1^H NMR spectrum of **11**. (300 MHz, CD_2_Cl_2_).

**Figure S4**. 2D ^1^H-^1^H COSY spectrum of **11** showing the proton coupling between protons. The insert is the simulated structure of **11**.

**Figure S5**. 2D ^1^H-^1^H NOESY spectrum of **11** showing the proton coupling between protons.

**Figure S6**. HR-MALDI-TOF mass spectra of **1** (a) and **2** (b).

**Figure S7**. ^1^H NMR spectrum of compound **1** (300 MHz, CD_2_Cl_2_).

**Figure S8**. ^1^H NMR spectrum of compound **2** (300 MHz, bromobenzene-d5).

**Figure S9**. Simulated UV-Vis spectra in toluene of **1** (a) and **2** (b) at B3LYP-D3(BJ)/6-31G(d) level of theory.

**Figure S10**. Time-resolved photo luminescence kinetic of **2**.

**Figure S11**. Synthesis and characterization of **2F**. (a) Synthetic of **2F**. (b) HR-MALDI-TOF mass spectra of **2F**. (c). Absorption (solid line) and emission (dashed line) spectra of **2F** in toluene. (d) Cyclic voltammetry (CV) and square wave voltammetry (SWV) of **2** and **2F** in a solution of Bu_4_NPF_6_ (0.1 M) in chlorobenzene with a scan rate of 100 mV s^−1^ at room temperature. (e) Simulated UV-Vis spectrum in toluene of **2F** at B3LYP-D3(BJ)/6-31G(d) level of theory. (f) Molecular orbitals and the energy diagrams of **2F** calculated at B3LYP-D3(BJ)/6-31G(d) level of theory with an implicit solvent toluene (PCM).

**Figure S12**. Chiral HPLC chromatogram of **1** (a) and **2** (b). Analysis was performed at room temperature using an SHIMADZU Labsolution system equipped with a (*R,R)*-Whelk-O 15 μm Kromasil column (25 cm x 21.1 mm). Eluent: CHCl_3_/n-hexane (v/v = 2:1); flow rate: 10 mL/min.

**Figure S13**. Comparison between experimental CD (dotted) and simulated CD spectra (solid), alongside with the theoretically calculated rotatory strengths of the calculated excited states (bars) for compound **1** (a) and **2** (b). Theoretical excited states and the simulated CD spectra are shifted (shift: 0.16 eV) to better match with the experiment.

**Figure S14**. Previously reported fjord-edged NGs.

| **Table S1.** Summary of the optical and chiroptical properties of reported fjord-edged NGs | | | | | | |  |  |
| --- | --- | --- | --- | --- | --- | --- | --- | --- |
| Compound | solvent | Temperature (^o^C) | \|*g*_abs_\| | \|*g*_lum_\| | | |  |  |
| **1** (this work) | toluene | 25 | 3.1 × 10^–3^ (538 nm) | None |  |  |  |  |
| **2** (this work) | toluene | 25 | 2.5 × 10^–2^ (500 nm) | 7.5 × 10^–4^ (815 nm) | | |  |  |
| C^1^ | CH_2_Cl_2_ | 0 | 4.2 × 10^–3^ (310 nm) | 2.1 × 10^–3^ (406 nm) | | |  |  |
| D^1^ | CH_2_Cl_2_ | 0 | 1.8 × 10^–3^ (370 nm) | 1.3 × 10^–3^ (480 nm) | | |  |  |
| E^2^ | CHCl_3_ | 20 | 2.3 × 10^–3^ (433 nm) | Not reported | | |  |  |
| F^3^ | CH_2_Cl_2_ | 25 | 2.7 × 10^–3^ (370 nm) | 2.3 × 10^–4^ (560 nm) | | |  |  |
| G^4^ | CH_2_Cl_2_ | 25 | 2.7 × 10^−4^ (550 nm) | 3.0 × 10^−4^ (643 nm) | | |  |  |
| H^5^ | toluene | 25 | 1.7 × 10^−2^ (616 nm) | 3.0 × 10^−4^ (643 nm) | | |  |  |
| I^6^ |  |  | Not reported | Not reported | | |  |  |
| J^7^ |  |  | Not reported | Not reported | | |  |  |
| K^8^ |  |  | Not reported | Not reported | | |  |  |
| L^9^ |  |  | Not reported | Not reported | | |  |  |

**Figure S15**. Circularly polarized luminescence (CPL) spectra of enantiomers of **2** in toluene; the light gray line shows the original, unsmoothed data.

**S2. Supplementary Methods**

**General Methods and Materials**

All the reagents were obtained from Sigma Aldrich, TCI, abcr, Alfa Aesar, Strem, fluorochem, or chempur. All these chemicals were used as received without further purification. All reactions dealing with air- or moisture-sensitive compounds were carried out in oven-dried glassware under an argon atmosphere. Anhydrous dichloromethane (DCM), toluene, and tetrahydrofuran (THF) were obtained from the MBRAUN MB-SPS-5 solvent purification system. All the sensitive reactions were performed using standard vacuum-line and Schlenk techniques. Thin layer chromatography (TLC) was performed on silica-coated aluminium sheets with a fluorescence indicator (TLC silica gel 60 F254, purchased from Merck KGaA). Column chromatography was performed on silica (SiO_2_, particle size 0.063-0.200 mm, purchased from VWR). Preparative recycling gel permeation chromatography (GPC) was performed on a Japan Analytical Industry JAI-HPLC LC 9110 II Next equipped with a JAIGEL-2HH and a JAIGEL-1HH column. Chloroform was used as the eluent in a flow rate of 10 mL/min.

**NMR spectra** were recorded using Bruker AV-II 300 MHz NMR spectrometers at room temperature (23°C). The NMR spectra were recorded using the standard Bruker pulse programs and processed using MestReNova software. Chemical shifts (δ) are expressed in ppm relative to the residual of solvents, CDCl_3_ (δ (1H) = 7.26 ppm, δ (13C) = 77.0 ppm), CD_2_Cl_2_ (δ (1H) = 5.32 ppm, δ (13C) = 53.8 ppm), and C_2_D_2_Cl_4_ (δ (1H) = 5.98 ppm, δ (13C) = 73.8 ppm). The following abbreviations are used to describe peak patterns as appropriate: s = singlet, d = doublet, t = triplet, q = quartet, and m = multiplet. Coupling constants (J) were recorded in Hertz.

**High resolution mass spectrometry (HRMS)** were performed on a Bruker Autoflex Speed MALDI TOF MS (Bruker Daltonics, Bremen, Germany) with the linear model using trans-2-[3-(4-tert-butylphenyl)-2-methyl-2-propenylidene]malononitrile (DCTB) as matrix.

**UV-visible spectra** were measured on an Agilent Cary 5000 UV-vis-NIR spectrophotometer by using a 10 mm optical-path quartz cell at room temperature. Fluorescence spectra were recorded at room temperature on FLS-1000 Fluorescence Spectrophotometer.

**Cyclic voltammetry (CV)** measurements and differential pulse voltammetry (DPV) were carried out on a CHI660E in a three-electrode cell. Electrolyte solutions were prepared by solving 0.1 M nBu_4_NPF_6_ in the solvents indicated and the experiments were performed with a scan rate of 100 mVs^-1^ at room temperature. A Pt electrode and a Pt wire were used as working and counter electrode, respectively. A silver wire was used as reference electrode.

**Circular dichroism (CD)** spectra were recorded using a Chirascan^TM^ V100 spectropolarimeter (Applied Photophysics, Ltd, Britain). The experiments were performed at room temperature in anhydrous toluene using a quartz cuvette with a 1 cm path length. The baseline was corrected by subtracting the spectrum of the neat solvent recorded under identical conditions. Circularly polarized luminescence (CPL) spectra were obtained using a JASCO CPL 300 spectrometer at 298 K.

Scheme S1. Synthetic routes towards **1** and **2**.

**Synthetic procedures and characterization**
((2-iodophenyl)ethynyl)triisopropylsilane (**15**)^10^, (3-bromonaphthalen-2-yl)boronic acid (**14**)^11^, ((2-(3-bromonaphthalen-2-yl)phenyl)ethynyl)triisopropylsilane (**13**)^10^, 2-bromo-3-(2-ethynylphenyl)naphthalene (**12**)^10^, 4-bromo-1,2-dihydrophenanthrene-3-carbaldehyde (**3**)^12^, 9,18-dibromobenzo[a]dinaphtho[2,1,8-cde:1',2',3',4'-ghi]perylene (**8**)^13^ were synthesized according to previous reported procedure.
**2-bromo-3-(2-(bromoethynyl)phenyl)naphthalene (11)**

To a degassed solution of compound **12** (500 mg, 1.63 mmol) in acetone (20 mL) were added NBS (432.14 mg, 2.44 mmol) and AgNO_3_ (27.65 mg, 162.77 μmol). The resulting mixture was stirred at room temperature for 2 h. After the reaction, the mixture was extracted three times with DCM, washed with brine and dried over MgSO_4_. The solvent was removed under reduced pressure and the residue was applied to chromatography on silica gel with eluent (iso-hexane:DCM = 6:1) to give compound **11** (550 mg, 87%).^1^H NMR (300 MHz, CD_2_Cl_2_) δ 8.22 (s, 1H), 7.85 (td, *J* = 5.0, 1.7 Hz, 2H), 7.81 (s, 1H), 7.63-7.53 (m, 3H), 7.50-7.34 (m, 3H). ^13^C NMR (76 MHz, CD_2_Cl_2_) δ 143.71, 138.37, 133.56, 132.36, 131.79, 130.80, 129.91, 128.20, 127.64, 126.86 (s), 126.45, 122.40, 120.90, 78.66.

**5,11-dibromochrysene (5)**

To a mixture of compound **11** (400 mg, 1.04 mmol) and InCl_3_ (458.28 mg, 2.07 mmol) was added degassed anhydrous toluene (40 mL). The reaction mixture was stirred at 100 °C for 24 h under argon atmosphere. After the reaction, the solvent was evaporated under reduced pressure and the residue was purified by flash chromatography on silica gel with eluent (iso-hexane/DCM=10:1) to give compound **5** (250 mg, 63%).^1^H NMR (300 MHz, CD_2_Cl_2_) δ 9.36 (dd, *J* = 6.9, 2.7 Hz, 2H), 8.30 (s, 2H), 7.86 (dd, *J* = 6.3, 3.1 Hz, 2H), 7.62 (pd, *J* = 6.9, 1.8 Hz, 4H). ^13^C NMR (76 MHz, CD_2_Cl_2_) δ 134.07, 132.32, 129.46, 128.93, 127.50, 126.64, 125.10, 115.85. HRMS (MALDI-TOF, m/z): calcd for [C_18_H_10_Br_2_]^+^, 385.9124; observed 385.9120, error = -1.04 ppm.

**5-(4,4,5,5-tetramethyl-1,3,2-dioxaborolan-2-yl)-1,2-dihydrophenanthrene-3-carbaldehyde (4)**

A 50 mL Schlenk flask containing a magnetic stirring bar was charged with **3** (1.00 g, 3.48 mmol), 4,4,4',4',5,5,5',5'-octamethyl-2,2'-bi(1,3,2-dioxaborolane) (1.33 g), Pd(dppf)Cl_2_ CH_2_Cl_2_·(284.39 mg, 348.24 μmol), KOAc (1.03 g, 10.45 mmol), and then 25 ml degassed 1,4-dioxane was added into flask. After the reaction stirring at 95 °C for 12 h, the mixture was cooled down, quenched with water and then extracted with DCM. The combined organic phase was dried over MgSO_4_ and concentrated under reduced pressure. The crude products were purified by column chromatography (iso-hexane/EtOAc= 5:1) to afford compound **4** (920 mg, 79% yield) as yellow solid. ^1^H NMR (300 MHz, CDCl_3_) δ 9.82 (s, 1H), 7.97 (s, 1H), 7.90 (ddd, *J* = 9.5, 7.5, 1.4 Hz, 2H), 7.82 (d, *J* = 8.2 Hz, 1H), 7.46 (dd, *J* = 8.1, 6.9 Hz, 1H), 7.35 (d, *J* = 8.2 Hz, 1H), 3.01 (t, *J* = 8.5 Hz, 2H), 2.68 (dd, *J* = 8.8, 7.9 Hz, 2H), 1.42 (s, 12H). ^13^C NMR (76 MHz, CDCl_3_) δ 193.52, 146.25, 140.62, 137.54, 136.47, 134.89, 134.08, 133.04, 132.63, 130.02, 127.61, 125.70, 85.35, 30.01, 26.02, 19.59.

**5,5'-(chrysene-4,11-diyl)bis(1,2-dihydrophenanthrene-3-carbaldehyde) (6)**

A 25 mL Schlenk flask containing a magnetic stirring bar was charged with **5** (50 mg, 129.50 µmol), **4** (130 mg, 388.51 µmol), CsF (78.69 mg, 518.02 µmol), Pd(PPh_3_)_4_ (29.93 mg, 25.90 µmol), and then 15 ml degassed DME was added into flask. After the reaction stirring at 85 °C for 12 h, the mixture was cooled down, quenched with water and then extracted with DCM. The combined organic phase was dried over MgSO_4_ and concentrated under reduced pressure. The crude products were purified by column chromatography (iso-hexane/EtOAc= 4:1) and recycle GPC to afford compound **6** (18 mg, 22% yield) as pale yellow solid. ^1^H NMR (300 MHz, CD_2_Cl_2_) δ 8.48 (s, 1H), 8.11 (dd, *J* = 6.7, 2.7 Hz, 1H), 8.02 (s, 1H), 8.01 – 7.94 (m, 4H), 7.88 – 7.79 (m, 4H), 7.77 – 7.70 (m, 3H), 7.41 (d, *J* = 7.3 Hz, 1H), 7.22 – 7.00 (m, 5H), 6.81 (d, *J* = 9.2 Hz, 1H), 6.56 (ddd, *J* = 8.5, 6.9, 1.4 Hz, 1H), 6.28 (d, *J* = 1.6 Hz, 1H), 5.95 (d, *J* = 1.9 Hz, 1H), 2.50 – 2.27 (m, 4H), 2.10 – 2.01 (m, 2H), 1.17 – 1.04 (m, 1H), 0.96 – 0.84 (m, 1H). ^13^C NMR (76 MHz, CD_2_Cl_2_) δ 191.96, 191.52, 144.90, 144.53, 141.54, 141.10, 140.93, 140.52, 139.78, 139.61, 139.58, 135.15, 134.08, 133.10, 132.31, 131.84, 131.16, 131.06, 130.96, 130.43, 130.38, 130.23, 129.79, 129.67, 129.46, 129.43, 129.37, 129.07, 129.03, 128.84, 128.72, 128.54, 128.29, 128.18, 127.20, 127.15, 126.84, 126.79, 126.46, 126.43, 125.98, 125.91, 125.70, 125.39, 124.91, 123.90, 28.74, 28.50, 16.68, 16.67. HRMS (MALDI-TOF, m/z): calcd for [C_48_H_32_O_2_]^+^, 640.2397; observed 640.2386, error = - 1.72 ppm.

**12,13-dimesitylbenzo[***rs***]indeno[2,1,7,6-***klmn***]naphtho[2,1,8,7-***defg***]phenanthro[1,2,3,4-***vwx***]hexaphene (1)**

Compound **6** (20 mg, 31.21 µmol) was dissolved in 10 mL of dry tetrahydrofuran (THF) under argon atmosphere. Mesitylmagnesium bromide solution (0.31 mL, 1M in THF, 0.31 mmol) was added to the solution and the mixture was stirred at room temperature for 12 hours. The reaction mixture was then poured into 50 mL water and extracted by DCM. The organic layer was dried over MgSO_4_ and the solvent was removed under reduced pressure. The obtained residue diol intermediate was then redissolved in 10 mL dry DCM under argon atmosphere and 2 mL of BF_3_·OEt_2_ was added. The mixture was stirred for 12 h and then washed with NaHCO_3_ solution (3x20 mL) and water (3x20 mL). The organic layer was dried over MgSO_4_ and the solvent was removed under reduced pressure. The residue was purified through a short pad of silica gel to yield coumpound **7**. The obtained compound **7** was then dissolved in 3 mL anhydrous toluene under argon atmosphere and 2,3-Dichloro-5,6-dicyano-1,4-benzoquinone (DDQ) (6 mg, 0.026 mmol, dissolved in 5 mL anhydrous toluene) was added dropwise. The mixture was stirred at 50 °C for 30 minutes. Then the residue was purified by column chromatography (silica gel, hexane:DCM = 2:1, v/v) to give compound **1** (6 mg, 23 % yield over three steps) as a green solid. ^1^H NMR (300 MHz, CD_2_Cl_2_) δ 9.32 (d, J = 7.9 Hz, 1H), 9.00 (d, J = 7.8 Hz, 1H), 8.83 (d, J = 8.5 Hz, 1H), 8.53 (d, J = 9.3 Hz, 1H), 8.40 (d, J = 7.1 Hz, 1H), 8.28 – 8.04 (m, 8H), 7.93 – 7.80 (m, 2H), 7.73 (d, J = 9.2 Hz, 1H), 7.53 – 7.42 (m, 3H), 7.22 – 7.14 (m, 1H), 6.87 – 6.72 (m, 3H), 6.42 (s, 1H), 2.37 (d, J = 4.4 Hz, 6H), 2.16 (s, 3H), 1.89 – 1.72 (m, 9H). HRMS (MALDI-TOF, m/z): calcd for [C_66_H_42_]^+^, 834.2381; observed 834. 3279 error = - 0.24 ppm.

**5,5'-(benzo[a]dinaphtho[2,1,8-cde:1',2',3',4'-ghi]perylene-9,18-diyl)bis(1,2-dihydrophenanthrene-3-carbaldehyde) (9)**

A 25 mL Schlenk flask containing a magnetic stirring bar was charged with **8** (50 mg, 0.044 mmol), (58 mg, 0.18 mmol), **9** (50 mg, 0.044 mmol), CsF (), (58 mg, 0.18 mmol), Pd(PPh_3_)_4_ (209 mg, 1.06 mmol), and then 5 ml degassed DME was added into flask. After the reaction stirring at 85 °C for 12 h, the mixture was cooled down, quenched with water and then extracted with DCM. The combined organic phase was dried over MgSO_4_ and concentrated under reduced pressure. The crude products were purified by column chromatography (iso-hexane/EtOAc= 2:1) and recycle GPC to afford compound **9** (33 mg, 36% yield) as orange solid. ^1^H NMR (300 MHz, C_2_D_2_Cl_4_) δ 8.91 (d, *J* = 8.4 Hz, 2H), 8.77 (d, *J* = 7.7 Hz, 2H), 8.64 (s, 2H), 8.43 (d, *J* = 7.8 Hz, 2H), 8.30 (d, *J* = 6.8 Hz, 2H), 8.17 (t, *J* = 7.8 Hz, 2H), 8.05 (d, *J* = 7.4 Hz, 2H), 7.84 (dd, *J* = 16.2, 8.2 Hz, 4H), 7.40 – 7.26 (m, 6H), 7.19 (d, *J* = 8.3 Hz, 2H), 6.76 (t, *J* = 7.7 Hz, 2H), 2.51 (dd, *J* = 16.2, 5.0 Hz, 2H), 2.24 (t, *J* = 16.7 Hz, 2H), 2.08 (dd, *J* = 15.5, 4.2 Hz, 2H), 1.05 (m, 2H). ^13^C NMR (76 MHz, C_2_D_2_Cl_4_) δ 191.42, 144.18, 140.86, 139.92, 139.66, 134.10, 132.11, 131.15, 130.68, 130.54, 130.28, 129.53, 129.09, 128.32, 126.49, 125.68, 124.91, 124.30, 123.87, 123.31, 28.80, 16.52. HRMS (MALDI-TOF, m/z): calcd for [C_66_H_38_O_2_]^+^, 862.2866; observed 862.2834 , error = - 3.71 ppm.

**Compound 2**

Compound **9** (20 mg, 23.17 µmol) was dissolved in 10 mL of dry THF under argon atmosphere. Mesitylmagnesium bromide solution ( 0.23 mL, 1M in THF, 0.23 mmol) was added to the solution and the mixture was stirred at room temperature for 12 hours. The reaction mixture was then poured into 50 mL water and extracted by DCM. The organic layer was dried over MgSO_4_ and the solvent was removed under reduced pressure. The obtained residue diol intermediate was then redissolved in 10 mL dry DCM under argon atmosphere and 2 mL of BF_3_·OEt_2_ was added. The mixture was stirred for 12 h and then washed with NaHCO_3_ solution (3x20 mL) and water (3x20 mL). The organic layer was dried over MgSO_4_ and the solvent was removed under reduced pressure. The residue was purified through a short pad of silica gel to yield coumpound **10**. The obtained compound **10** was then dissolved in 3 mL anhydrous toluene under argon atmosphere and DDQ (26 mg, 0.12 mmol, dissolved in 5 mL anhydrous toluene) was added dropwise. The mixture was stirred at 50 °C for 30 minutes. Then the residue was purified by column chromatography (silica gel, hexane:DCM = 2:1, v/v) to give compound **2** ( 6 mg, 24% yield over three steps) as a green solid. ^1^H NMR (300 MHz, bromobenzene-d5) δ 9.87 (d, *J* = 7.8 Hz, 2H), 9.60 (d, *J* = 9.6 Hz, 2H), 9.08 (dd, *J* = 14.6, 8.0 Hz, 4H), 8.43 (d, *J* = 8.7 Hz, 2H), 8.34 (d, *J* = 8.8 Hz, 2H), 8.26 (d, *J* = 9.6 Hz, 2H), 8.14 (d, *J* = 8.7 Hz, 2H), 8.07 (dd, *J* = 8.1, 4.4 Hz, 4H), 7.65 (t, *J* = 7.8 Hz, 2H), 7.56 (t, *J* = 7.5 Hz, 2H), 7.36 (s, 2H), 7.25 (s, 4H), 2.59 (s, 6H), 2.28 (s, 6H), 1.93 (s, 6H). HRMS (MALDI-TOF, m/z): calcd for [C_84_H_48_]^+^ 1056.3750, observed 1056.3742 , error = - 0.75 ppm.

**Compound 2F**

To a mixture of **2** (5 mg, 4.73 µmol) and 2,3-dichloro-5,6-dicyano-1,4-benzoquinone (DDQ) (7 mg, 30.75 µmol) in dry dichloromethane (DCM) (10 mL) at 0 °C was added trifluoromethanesulfonic acid (0.5 mL). After stirring for 1 hour, the resulting mixture was quenched by saturated NaHCO_3_ solution (20 mL), and then extracted with DCM twice. The combined organic layers were washed with brine and dried over MgSO_4_. After removal of the solvent under reduced pressure, the crude product was purified through a short pad of silica gel and recrystallized from hot chlorobenzene to afford **2F** (4 mg, 80 %) as a green solid. HRMS (MALDI-TOF, m/z): calcd for [C_84_H_44_]^+^ 1052.3438; observed 1052.3440 , error = 0.19 ppm.

**S3. Crystallographic information**

The crystal was then mounted and measured on a Rigaku XtaLAB Synergy Diffractometer and the temperature of the crystal was controlled by Oxford Cryostream 700. Using Olex2^14^, the initial structure was solved with the SHELX-XT structure solution program using direct method and refined with the XL refinement package using Least Squares minimization^15^. Crystallographic data were deposited in the Cambridge Crystallographic Data Centre (CCDC 2541397-2541401). The data can be obtained free of charge from The Cambridge Crystallographic Data Centre via www.ccdc.cam.ac.uk/data_request/cif.

| **Table S2**. Crystal data and structure refinement for compound **4** | |
| --- | --- |
| **CCDC No** | 2541397 |
| **Empirical formula** | C_21_H_23_BO_3_ |
| **Formula weight** | 334.2 |
| **Temperature/K** | 100.0(6) |
| **Crystal system** | monoclinic |
| **Space group** | P2_1_/c |
| **a/Å** | 10.0274(5) |
| **b/Å** | 13.5209(7) |
| **c/Å** | 13.1215(6) |
| **α/°** | 90 |
| **β/°** | 94.017(5) |
| **γ/°** | 90 |
| **Volume/Å^3^** | 1774.64(15) |
| **Z** | 4 |
| **ρ_calc_g/cm^3^** | 1.251 |
| **μ/mm^‑1^** | 0.644 |
| **F(000)** | 712 |
| **Crystal size/mm^3^** | 0.5 × 0.3 × 0.2 |
| **Radiation** | CuKα (λ = 1.54184) |
| **2Θ range for data collection/°** | 8.84 to 149.21 |
| **Index ranges** | -8 ≤ h ≤ 12, -16 ≤ k ≤ 15, -16 ≤ l ≤ 16 |
| **Reflections collected** | 12283 |
| **Independent reflections** | 3476 [R_int_= 0.0713, R_sigma_= 0.0640] |
| **Data/restraints/parameters** | 3476/0/230 |
| **Goodness-of-fit on F^2^** | 1.05 |
| **Final R indexes [I>=2σ (I)]** | R_1_= 0.0637, wR_2_= 0.1707 |
| **Final R indexes [all data]** | R_1_= 0.0794, wR_2_= 0.1900 |
| **Largest diff. peak/hole / e Å^-3^** | 0.34/-0.23 |

| **Table S3**. Crystal data and structure refinement for compound **6** | |
| --- | --- |
| **CCDC No** | 2541399 |
| **Empirical formula** | C_48_H_32_O_2_ |
| **Formula weight** | 640.73 |
| **Temperature/K** | 100.00(10) |
| **Crystal system** | monoclinic |
| **Space group** | P2_1_/n |
| **a/Å** | 9.2304(2) |
| **b/Å** | 8.5407(2) |
| **c/Å** | 41.0600(8) |
| **α/°** | 90 |
| **β/°** | 95.685(2) |
| **γ/°** | 90 |
| **Volume/Å^3^** | 3221.01(12) |
| **Z** | 4 |
| **ρ_calc_g/cm^3^** | 1.321 |
| **μ/mm^‑1^** | 0.614 |
| **F(000)** | 1344 |
| **Crystal size/mm^3^** | 0.5 × 0.1 × 0.1 |
| **Radiation** | CuKα (λ = 1.54184) |
| **2Θ range for data collection/°** | 8.656 to 153.076 |
| **Index ranges** | -11 ≤ h ≤ 8, -10 ≤ k ≤ 10, -50 ≤ l ≤ 51 |
| **Reflections collected** | 21185 |
| **Independent reflections** | 6453 [R_int_= 0.0310, R_sigma_= 0.0312] |
| **Data/restraints/parameters** | 6453/0/451 |
| **Goodness-of-fit on F^2^** | 1.044 |
| **Final R indexes [I>=2σ (I)]** | R_1_= 0.0479, wR_2_= 0.1250 |
| **Final R indexes [all data]** | R_1_= 0.0617, wR_2_= 0.1327 |
| **Largest diff. peak/hole / e Å^-3^** | 0.23/-0.21 |

| **Table S4**. Crystal data and structure refinement for compound **9** | |
| --- | --- |
| **CCDC No** | 2541400 |
| **Empirical formula** | C_66_H_38_O_2_ |
| **Formula weight** | 862.96 |
| **Temperature/K** | 100.00(10) |
| **Crystal system** | monoclinic |
| **Space group** | C2/c |
| **a/Å** | 11.2506(2) |
| **b/Å** | 20.8408(4) |
| **c/Å** | 19.0269(3) |
| **α/°** | 90 |
| **β/°** | 92.585(2) |
| **γ/°** | 90 |
| **Volume/Å^3^** | 4456.73(14) |
| **Z** | 4 |
| **ρ_calc_g/cm^3^** | 1.286 |
| **μ/mm^‑1^** | 0.589 |
| **F(000)** | 1800 |
| **Crystal size/mm^3^** | 0.6 × 0.1 × 0.1 |
| **Radiation** | CuKα (λ = 1.54184) |
| **2Θ range for data collection/°** | 8.486 to 153.538 |
| **Index ranges** | -11 ≤ h ≤ 14, -25 ≤ k ≤ 24, -24 ≤ l ≤ 24 |
| **Reflections collected** | 18010 |
| **Independent reflections** | 4542 [R_int_= 0.0310, R_sigma_= 0.0308] |
| **Data/restraints/parameters** | 4542/0/307 |
| **Goodness-of-fit on F^2^** | 1.028 |
| **Final R indexes [I>=2σ (I)]** | R_1_= 0.0443, wR_2_= 0.1214 |
| **Final R indexes [all data]** | R_1_= 0.0523, wR_2_= 0.1276 |
| **Largest diff. peak/hole / e Å^-3^** | 0.21/-0.25 |

| **Table S5**. Crystal data and structure refinement for compound **1** | |
| --- | --- |
| **CCDC No** | 2541401 |
| **Empirical formula** | C_66_H_42_ |
| **Formula weight** | 834.99 |
| **Temperature/K** | 100.00(10) |
| **Crystal system** | tetragonal |
| **Space group** | P4_2_/n |
| **a/Å** | 25.9268(3) |
| **b/Å** | 25.9268(3) |
| **c/Å** | 12.3582(2) |
| **α/°** | 90 |
| **β/°** | 90 |
| **γ/°** | 90 |
| **Volume/Å^3^** | 8307.2(2) |
| **Z** | 8 |
| **ρ_calc_g/cm^3^** | 1.335 |
| **μ/mm^‑1^** | 0.574 |
| **F(000)** | 3504 |
| **Crystal size/mm^3^** | 0.5 × 0.2 × 0.1 |
| **Radiation** | CuKα (λ = 1.54184) |
| **2Θ range for data collection/°** | 4.82 to 156.318 |
| **Index ranges** | -32 ≤ h ≤ 18, -29 ≤ k ≤ 17, -13 ≤ l ≤ 15 |
| **Reflections collected** | 29611 |
| **Independent reflections** | 8469 [R_int_= 0.0466, R_sigma_= 0.0411] |
| **Data/restraints/parameters** | 8469/0/601 |
| **Goodness-of-fit on F^2^** | 1.136 |
| **Final R indexes [I>=2σ (I)]** | R_1_= 0.0812, wR_2_= 0.1793 |
| **Final R indexes [all data]** | R_1_= 0.0927, wR_2_= 0.1844 |
| **Largest diff. peak/hole / e Å^-3^** | 0.49/-0.25 |

| **Table S6**. Crystal data and structure refinement for compound **2** | |
| --- | --- |
| **CCDC No** | 2541398 |
| **Empirical formula** | C_84_H_48_ |
| **Formula weight** | 1057.22 |
| **Temperature/K** | 100.00(10) |
| **Crystal system** | monoclinic |
| **Space group** | C2/c |
| **a/Å** | 27.8460(13) |
| **b/Å** | 18.8598(8) |
| **c/Å** | 26.2323(8) |
| **α/°** | 90 |
| **β/°** | 101.784(4) |
| **γ/°** | 90 |
| **Volume/Å^3^** | 13486.1(10) |
| **Z** | 8 |
| **ρ_calc_g/cm^3^** | 1.041 |
| **μ/mm^‑1^** | 0.45 |
| **F(000)** | 4416 |
| **Crystal size/mm^3^** | 0.5 × 0.1 × 0.1 |
| **Radiation** | CuKα (λ = 1.54184) |
| **2Θ range for data collection/°** | 6.306 to 130.176 |
| **Index ranges** | -32 ≤ h ≤ 32, -19 ≤ k ≤ 22, -27 ≤ l ≤ 30 |
| **Reflections collected** | 37549 |
| **Independent reflections** | 11366 [R_int_= 0.0864, R_sigma_= 0.0811] |
| **Data/restraints/parameters** | 11366/0/763 |
| **Goodness-of-fit on F^2^** | 0.975 |
| **Final R indexes [I>=2σ (I)]** | R_1_= 0.0898, wR_2_= 0.2357 |
| **Final R indexes [all data]** | R_1_= 0.1231, wR_2_= 0.2630 |
| **Largest diff. peak/hole / e Å^-3^** | 0.45/-0.35 |

**S4. DFT calculations**

**Mechanistic investigations part**: All the structures were optimized in the gas phase by the B3LYP density functional^16^ with the def2-SVP basis set^17^ by Gaussian 16 software package^18^. The DFT-D3(BJ) method was used for dispersion correction.^19-21^ Frequency analysis at the same level of theory were calculated to confirm that all stationary points were minima (no imaginary frequencies) or transition states (one imaginary frequency). The single-point energies were calculated at the B3LYP-D3(BJ)/def2-TZVP level. The solution model density (SMD) was used for modelling the solvent effect6 in CH2Cl2 for path (a) and in toluene for path (b).

**Chiral optical properties part**: All calculations were performed within the framework of density functional theory (DFT) using the B3LYP functional and the 6-31G(d) basis set, implemented in Gaussian16 software package^22^. The systems were modelled in the gas phase as isolated molecules. The fully optimized structures **1** and **2**, as well as the aromatic cores for the different isomers (*P^5^M^5’^*)-**1**, (*M^5^M^5’^*)-**1**, (*M^5^P^5’^*)-**1** and (*M^5^P^4^M^5^P^4^*)-**2**, (*M^5^P^4^M^5^M^4^*)-**2**, (*M^5^P^4^P^5^M^4^*)-**2** are obtained with no imaginary frequencies. The transition states (*1-TS^5^*)-**1**, (*2-TS^5'^*)-**1**, (*1-TS^4^*)-**2,** and (*2-TS^5^*)-**2** were characterized by a single imaginary frequency, the vibrational mode of which corresponds to the helicene rotation along the reaction coordinate.

For transition state (TS) calculation, the structures were optimized in the gas phase with B3LYP/6-31G(d). For the isomerization process, energy barriers were characterized by Gibbs free energies (ΔG^‡^), ensuring a comprehensive thermodynamic description of the reaction profile. Note: The superscripts represent the size of the [n]helicene subunit.

To simulate the UV-Vis and CD spectra of the molecules, structures of **1** and **2** were optimized with a solvent *Toluene* included implicitly (polarizable continuum model, PCM)^23-25^ and the Grimme’s D3 empirical correction with Becke-Johnson (BJ) damping^20^. Then, excitation energies were calculated using TD-DFT formalism with 80 excited states. Structures and isosurfaces (isovalue = 0.02) were visualized using Multiwfn 3.8^26, 27^, Avogadro 1.2 software^28^ and VMD 1.9.4a53^29^.

**Figure S16.** Optimized structures of **1** (a), **2** (b) and **2F** (c).

| **Table S7**. Thermal corrections to free energies (*G*_corr,def2-SVP_) and electronic energies (*E*_def2-TZVP_) of the intermediates and other molecules in path (a) and (b). | | | |
| --- | --- | --- | --- |
| path |  | *G*_corr,def2-SVP_ (Hartree) | *E*_def2-TZVP_ (Hartree) |
| a | BF_3_∙OEt_2_ | 0.114781 | -558.5242503 |
|  | BF_3_∙OH^-^ | -0.00335 | -400.7707681 |
|  | OEt_2_ | 0.105777 | -233.7811438 |
|  | BF_3_∙OH_2_ | 0.006504 | -401.2068016 |
|  | **R1** | 0.939066 | -2700.619578 |
|  | **INT1** | 0.921121 | -2624.573085 |
|  | **TS1** | 0.923988 | -2624.554136 |
|  | **INT2** | 0.926434 | -2624.564908 |
|  | **INT2**′ | 0.926626 | -2624.523995 |
|  | **INT3** | 0.903192 | -2624.136110 |
|  | **INT4** | 0.902121 | -2548.097474 |
|  | **TS2** | 0.901664 | -2548.067499 |
|  | **INT5** | 0.902935 | -2548.074153 |
|  | **7** | 0.887930 | -2547.656235 |
| b | DDQ | 0.023303 | -1485.426786 |
|  | DDQH^∙-^ | 0.035175 | -1486.206153 |
|  | DDQH_2_ | 0.048273 | -1486.685808 |
|  | **7** | 0.887930 | -2547.647405 |
|  | **INT6** | 0.87698 | -2546.889683 |
|  | **TS3** | 0.880149 | -2546.857735 |
|  | **INT7** | 0.883714 | -2546.890212 |
|  | **INT8** | 0.874564 | -2546.495289 |
|  | **INT9** | 0.864835 | -2545.734649 |
|  | **TS4** | 0.861768 | -2545.692619 |
|  | **INT10** | 0.86434 | -2545.698975 |
|  | **INT11** | 0.856372 | -2545.29836 |
|  | **1** | 0.788642 | -2541.724167 |

| Table S8. First five UV-Vis transitions of 1 and 2. | | | | |
| --- | --- | --- | --- | --- |
|  | **Excited state** | **Molecular Orbital** | **Wavelength, nm** | **Oscillator strength, a.u.** |
| 1 | **S_0_ → S_1_*** | H-1 → L (3%)  H → L (96%) | 731.92 | 0.4608 |
|  | **S_0_ → S_2_*** | H-1 → L (93%)  H → L (3%) | 603.07 | 0.1012 |
|  | **S_0_ → S_3_*** | H-2 → L (94%) | 510.26 | 0.1206 |
|  | **S_0_ → S_4_*** | H-3 → L (90%)  H → L+2 (5%) | 495.26 | 0.0226 |
|  | **S_0_ → S_5_*** | H-4 → L (3%)  H → L+1 (93%) | 481.21 | 0.0257 |
| 2 | **S_0_ → S_1_*** | H → L (100%) | 821.34 | 1.5213 |
|  | **S_0_ → S_2_*** | H-1 → L (46%)  H → L+1 (53%) | 591.44 | 0 |
|  | **S_0_ → S_3_*** | H-2 → L (36%)  H → L+2 (61%) | 533.14 | 0.0008 |
|  | **S_0_ → S_4_*** | H-1 → L (52%)  H → L+1 (46%) | 531.77 | 0.1187 |
|  | **S_0_ → S_5_*** | H-2 → L (48%)  H → L+2 (27%)  H → L+3 (22%) | 464.84 | 0.2718 |

| Table S9. CD transitions, transition dipole moments, rotatory strengths and *g*_calc_ of compound 1 | | | | | | |
| --- | --- | --- | --- | --- | --- | --- |
| Excited state | **λ, nm** | **\|*µ*_e_\|, × 10^-20^ esu ⋅ cm** | **\|*µ*_m_\|, × 10^-20^ erg ⋅ G^-1^** | ***R*_vel_, × 10^-40^ erg ⋅ esu ⋅ cm ⋅ G^-1^** | **θ** | ***g*_calc_** |
| 1 | 737.9 | 846.4 | 0.7 | -34.8 | 93.3 | -1.94E-04 |
| 2 | 608.0 | 360.1 | 1.6 | -94.9 | 99.4 | -2.92E-03 |
| 3 | 514.4 | 361.6 | 0.3 | 70.9 | 50.1 | 2.17E-03 |
| 4 | 499.3 | 154.2 | 0.4 | -68.4 | 172.3 | -1.15E-02 |
| 5 | 485.2 | 161.9 | 0.5 | -52.8 | 134.1 | -8.04E-03 |
| 6 | 465.3 | 183.9 | 1.0 | -1.7 | 90.5 | -2.00E-04 |
| 7 | 441.1 | 147.7 | 0.7 | 70.9 | 43.6 | 1.30E-02 |
| 8 | 433.2 | 69.3 | 0.3 | 17.1 | 39.2 | 1.43E-02 |
| 9 | 423.2 | 75.0 | 0.1 | -5.9 | 138.4 | -4.18E-03 |
| 10 | 415.4 | 457.4 | 0.5 | -61.1 | 106.6 | -1.17E-03 |
| 11 | 408.4 | 120.4 | 0.8 | 30.3 | 72.7 | 8.34E-03 |
| 12 | 393.7 | 261.9 | 1.7 | -24.9 | 93.2 | -1.45E-03 |
| 13 | 389.7 | 269.7 | 0.7 | 124.2 | 45.9 | 6.82E-03 |
| 14 | 381.0 | 168.9 | 0.9 | 57.2 | 66.8 | 8.01E-03 |
| 15 | 375.2 | 311.2 | 0.3 | 67.3 | 40.7 | 2.78E-03 |
| 16 | 371.0 | 165.0 | 2.5 | -65.9 | 99.2 | -9.66E-03 |
| 17 | 359.7 | 60.1 | 0.4 | 10.3 | 64.7 | 1.15E-02 |
| 18 | 356.8 | 147.4 | 0.2 | 6.0 | 78.5 | 1.10E-03 |
| 19 | 349.2 | 216.2 | 0.3 | -47.3 | 140.1 | -4.04E-03 |
| 20 | 343.9 | 289.3 | 0.3 | -46.7 | 118.0 | -2.23E-03 |
| 21 | 341.3 | 290.1 | 0.1 | -17.3 | 113.9 | -8.23E-04 |
| 22 | 333.0 | 239.5 | 0.8 | 34.5 | 79.5 | 2.41E-03 |
| 23 | 331.0 | 379.0 | 0.3 | -19.3 | 101.4 | -5.37E-04 |
| 24 | 327.1 | 302.5 | 0.3 | 22.7 | 75.4 | 9.93E-04 |
| 25 | 326.4 | 110.7 | 0.7 | -9.8 | 97.6 | -3.20E-03 |
| 26 | 320.8 | 228.5 | 1.3 | -42.5 | 98.0 | -3.25E-03 |
| 27 | 317.7 | 181.7 | 0.4 | 48.9 | 52.1 | 5.91E-03 |
| 28 | 315.7 | 61.4 | 0.7 | -25.9 | 126.4 | -2.74E-02 |
| 29 | 315.4 | 168.4 | 0.9 | 16.4 | 83.8 | 2.32E-03 |
| 30 | 313.8 | 113.4 | 1.1 | 75.6 | 52.7 | 2.35E-02 |
| 31 | 310.5 | 414.0 | 0.4 | 159.7 | 19.7 | 3.72E-03 |
| 32 | 307.7 | 92.2 | 0.1 | 3.9 | 70.2 | 1.84E-03 |
| 33 | 306.0 | 464.5 | 0.2 | 15.8 | 79.0 | 2.93E-04 |
| 34 | 304.8 | 206.6 | 0.4 | 15.3 | 79.9 | 1.44E-03 |
| 35 | 301.7 | 107.2 | 0.2 | 9.4 | 61.3 | 3.27E-03 |
| 36 | 299.4 | 293.7 | 0.5 | 41.2 | 74.1 | 1.91E-03 |
| 37 | 295.8 | 165.4 | 1.1 | -40.8 | 102.6 | -5.96E-03 |
| 38 | 294.0 | 183.2 | 0.4 | 38.6 | 58.9 | 4.60E-03 |
| 39 | 292.3 | 111.2 | 0.7 | -1.4 | 91.1 | -4.64E-04 |
| 40 | 291.5 | 188.0 | 0.6 | -31.5 | 105.9 | -3.56E-03 |
| 41 | 290.1 | 54.7 | 0.2 | -8.2 | 143.3 | -1.10E-02 |
| 42 | 289.4 | 164.6 | 0.7 | -49.2 | 114.7 | -7.26E-03 |
| 43 | 288.5 | 260.0 | 0.5 | -12.6 | 95.9 | -7.44E-04 |
| 44 | 286.6 | 152.9 | 0.7 | -33.1 | 108.8 | -5.66E-03 |
| 45 | 285.6 | 137.6 | 0.3 | 16.7 | 63.1 | 3.53E-03 |
| 46 | 284.8 | 115.7 | 1.0 | -86.7 | 138.9 | -2.59E-02 |
| 47 | 282.4 | 197.9 | 0.7 | -17.1 | 96.9 | -1.75E-03 |
| 48 | 282.3 | 140.9 | 0.2 | 9.3 | 72.9 | 1.87E-03 |
| 49 | 281.8 | 142.7 | 0.4 | -27.4 | 119.9 | -5.37E-03 |
| 50 | 281.7 | 249.4 | 0.4 | 2.5 | 88.6 | 1.58E-04 |
| 51 | 279.0 | 14.1 | 0.8 | -7.7 | 134.0 | -1.54E-01 |
| 52 | 277.7 | 213.6 | 0.3 | 13.5 | 76.1 | 1.18E-03 |
| 53 | 276.7 | 181.2 | 0.4 | -16.8 | 104.5 | -2.04E-03 |
| 54 | 276.1 | 213.2 | 0.7 | -80.7 | 123.1 | -7.09E-03 |
| 55 | 274.9 | 217.6 | 1.2 | -122.1 | 117.3 | -1.03E-02 |
| 56 | 274.6 | 158.2 | 1.0 | 20.6 | 82.5 | 3.28E-03 |
| 57 | 272.9 | 315.7 | 1.0 | 2.9 | 89.5 | 1.17E-04 |
| 58 | 271.8 | 61.5 | 0.4 | 4.7 | 79.0 | 5.01E-03 |
| 59 | 271.6 | 80.2 | 0.7 | -7.1 | 96.8 | -4.38E-03 |
| 60 | 270.6 | 225.1 | 0.4 | -55.7 | 125.6 | -4.39E-03 |
| 61 | 267.9 | 33.4 | 0.3 | 2.2 | 78.7 | 7.69E-03 |
| 62 | 267.0 | 58.8 | 0.2 | -9.3 | 129.8 | -1.08E-02 |
| 63 | 265.6 | 75.8 | 0.3 | -4.2 | 100.3 | -2.92E-03 |
| 64 | 264.1 | 123.6 | 1.3 | 91.0 | 56.9 | 2.38E-02 |
| 65 | 263.7 | 46.1 | 0.7 | -27.8 | 150.4 | -5.22E-02 |
| 66 | 262.3 | 212.5 | 2.3 | 16.6 | 88.0 | 1.47E-03 |
| 67 | 261.3 | 182.0 | 0.1 | 20.0 | 41.1 | 2.41E-03 |
| 68 | 260.8 | 118.8 | 0.9 | -29.2 | 105.8 | -8.28E-03 |
| 69 | 260.1 | 33.5 | 0.1 | -3.6 | 144.0 | -1.27E-02 |
| 70 | 259.9 | 49.7 | 0.1 | 0.5 | 86.3 | 7.39E-04 |
| 71 | 259.5 | 80.5 | 0.8 | 40.3 | 48.3 | 2.48E-02 |
| 72 | 257.5 | 258.6 | 1.0 | -56.9 | 102.3 | -3.40E-03 |
| 73 | 256.7 | 146.5 | 0.1 | 7.0 | 31.1 | 1.29E-03 |
| 74 | 256.4 | 111.1 | 0.3 | 18.1 | 57.9 | 5.86E-03 |
| 75 | 255.8 | 20.5 | 0.9 | -7.9 | 114.7 | -7.49E-02 |
| 76 | 255.0 | 162.4 | 0.2 | -14.7 | 114.2 | -2.23E-03 |
| 77 | 254.6 | 174.7 | 0.3 | -43.1 | 157.8 | -5.65E-03 |
| 78 | 254.2 | 109.4 | 0.9 | -37.9 | 111.7 | -1.27E-02 |
| 79 | 253.2 | 132.3 | 0.8 | -23.9 | 102.6 | -5.45E-03 |
| 80 | 252.9 | 139.2 | 0.3 | -5.8 | 98.6 | -1.20E-03 |

| Table S10. CD transitions, transition dipole moments, rotatory strengths and *g*_calc_ of compound 2 | | | | | | |
| --- | --- | --- | --- | --- | --- | --- |
| Excited state | **λ, nm** | **\|*µ*_e_\|, × 10^-20^ esu ⋅ cm** | **\|*µ*_m_\|, × 10^-20^ erg ⋅ G^-1^** | ***R*_vel_, × 10^-40^ erg ⋅ esu ⋅ cm ⋅ G^-1^** | **θ** | ***g*_calc_** |
| 1 | 821.5 | 1629.1 | 0.8 | -371.9 | 106.1 | -5.60E-04 |
| 2 | 591.5 | 1.1 | 0.1 | -0.1 | 171.6 | -3.04E-01 |
| 3 | 533.2 | 30.9 | 0.1 | -1.7 | 133.2 | -7.21E-03 |
| 4 | 531.8 | 366.1 | 2.3 | 826.4 | 0.0 | 2.46E-02 |
| 5 | 464.9 | 518.0 | 0.4 | -124.0 | 124.2 | -1.85E-03 |
| 6 | 457.5 | 6.3 | 1.7 | -10.6 | 178.0 | -1.06E+00 |
| 7 | 454.9 | 264.4 | 0.1 | -22.8 | 168.5 | -1.30E-03 |
| 8 | 449.8 | 13.4 | 2.9 | 38.8 | 0.6 | 8.66E-01 |
| 9 | 443.7 | 5.3 | 0.1 | 0.5 | 2.5 | 7.39E-02 |
| 10 | 435.0 | 451.3 | 0.4 | -167.2 | 176.8 | -3.28E-03 |
| 11 | 406.6 | 8.4 | 3.0 | -24.7 | 169.2 | -1.40E+00 |
| 12 | 405.8 | 119.1 | 0.1 | -14.2 | 153.7 | -4.00E-03 |
| 13 | 404.6 | 255.9 | 0.4 | -85.8 | 153.9 | -5.23E-03 |
| 14 | 395.0 | 3.6 | 0.3 | -0.8 | 134.9 | -2.51E-01 |
| 15 | 394.8 | 39.1 | 0.1 | 4.1 | 34.1 | 1.06E-02 |
| 16 | 393.0 | 205.0 | 0.6 | 95.5 | 39.8 | 9.08E-03 |
| 17 | 381.2 | 12.1 | 0.6 | 6.7 | 0.5 | 1.82E-01 |
| 18 | 377.1 | 12.9 | 0.1 | -0.8 | 139.0 | -1.94E-02 |
| 19 | 376.6 | 6.0 | 0.2 | 1.4 | 2.7 | 1.60E-01 |
| 20 | 376.5 | 12.3 | 0.1 | -1.3 | 176.1 | -3.38E-02 |
| 21 | 375.6 | 24.6 | 0.2 | 5.6 | 2.7 | 3.68E-02 |
| 22 | 364.9 | 20.6 | 0.5 | -10.5 | 178.6 | -9.85E-02 |
| 23 | 362.0 | 22.9 | 0.6 | -14.6 | 177.8 | -1.11E-01 |
| 24 | 356.2 | 293.4 | 0.1 | 28.8 | 21.0 | 1.34E-03 |
| 25 | 355.1 | 32.3 | 2.1 | 68.2 | 1.1 | 2.61E-01 |
| 26 | 347.2 | 14.9 | 1.3 | 19.2 | 1.7 | 3.45E-01 |
| 27 | 345.9 | 69.3 | 0.2 | 7.3 | 54.1 | 6.06E-03 |
| 28 | 338.5 | 32.3 | 0.3 | -11.3 | 179.8 | -4.32E-02 |
| 29 | 336.5 | 400.1 | 0.5 | -123.2 | 124.5 | -3.08E-03 |
| 30 | 336.4 | 52.8 | 0.8 | 39.7 | 16.3 | 5.69E-02 |
| 31 | 334.0 | 746.6 | 0.8 | -212.3 | 110.5 | -1.52E-03 |
| 32 | 329.8 | 226.6 | 0.0 | 4.0 | 67.4 | 3.08E-04 |
| 33 | 324.7 | 576.9 | 0.1 | 8.4 | 78.3 | 1.01E-04 |
| 34 | 321.2 | 11.2 | 0.5 | -5.6 | 178.9 | -1.79E-01 |
| 35 | 320.0 | 272.7 | 0.2 | -54.5 | 148.3 | -2.93E-03 |
| 36 | 317.8 | 3.8 | 0.3 | 1.0 | 3.1 | 2.71E-01 |
| 37 | 316.1 | 278.3 | 0.1 | -9.1 | 102.8 | -4.72E-04 |
| 38 | 315.8 | 375.1 | 0.2 | 15.7 | 77.8 | 4.47E-04 |
| 39 | 315.0 | 5.8 | 0.7 | -3.4 | 151.9 | -3.99E-01 |
| 40 | 313.8 | 273.0 | 0.1 | 16.3 | 61.4 | 8.76E-04 |
| 41 | 310.1 | 438.6 | 0.1 | -12.9 | 116.5 | -2.67E-04 |
| 42 | 309.0 | 47.7 | 2.2 | 103.0 | 1.6 | 1.80E-01 |
| 43 | 306.8 | 28.5 | 3.6 | 103.4 | 0.9 | 5.09E-01 |
| 44 | 304.9 | 148.8 | 0.4 | 0.1 | 89.9 | 9.55E-06 |
| 45 | 302.9 | 183.8 | 0.3 | -34.3 | 128.2 | -4.06E-03 |
| 46 | 302.4 | 100.2 | 1.7 | -168.7 | 179.4 | -6.71E-02 |
| 47 | 301.9 | 88.9 | 0.4 | -28.1 | 146.3 | -1.42E-02 |
| 48 | 301.9 | 108.0 | 0.2 | -2.7 | 99.2 | -9.37E-04 |
| 49 | 301.4 | 47.0 | 0.9 | 40.9 | 1.2 | 7.41E-02 |
| 50 | 299.1 | 54.5 | 0.2 | -9.5 | 179.5 | -1.27E-02 |
| 51 | 298.5 | 25.7 | 0.2 | 0.4 | 85.9 | 2.57E-03 |
| 52 | 297.0 | 0.7 | 0.3 | 0.2 | 32.1 | 1.20E+00 |
| 53 | 296.6 | 36.4 | 1.1 | 40.6 | 0.7 | 1.22E-01 |
| 54 | 295.0 | 177.8 | 0.2 | 31.3 | 13.2 | 3.95E-03 |
| 55 | 293.7 | 51.0 | 1.0 | -49.4 | 179.7 | -7.60E-02 |
| 56 | 292.4 | 71.1 | 0.2 | -9.5 | 127.8 | -7.48E-03 |
| 57 | 292.1 | 90.1 | 0.2 | -15.4 | 140.4 | -7.57E-03 |
| 58 | 292.1 | 9.6 | 1.1 | 10.3 | 2.7 | 4.44E-01 |
| 59 | 291.9 | 27.4 | 0.7 | 18.3 | 5.2 | 9.77E-02 |
| 60 | 290.9 | 547.3 | 0.7 | -134.2 | 110.1 | -1.79E-03 |
| 61 | 289.8 | 179.4 | 1.0 | 179.0 | 0.1 | 2.22E-02 |
| 62 | 286.8 | 135.3 | 0.7 | 64.9 | 44.5 | 1.42E-02 |
| 63 | 286.6 | 174.6 | 0.2 | -16.8 | 122.8 | -2.21E-03 |
| 64 | 286.5 | 242.8 | 0.2 | 15.9 | 70.3 | 1.08E-03 |
| 65 | 285.8 | 88.6 | 0.3 | 23.3 | 0.1 | 1.18E-02 |
| 66 | 284.3 | 16.2 | 0.3 | 4.1 | 1.4 | 6.20E-02 |
| 67 | 283.7 | 28.4 | 0.1 | -1.9 | 130.5 | -9.28E-03 |
| 68 | 283.3 | 69.2 | 0.7 | 46.0 | 0.4 | 3.84E-02 |
| 69 | 281.7 | 113.2 | 0.3 | -16.9 | 118.7 | -5.27E-03 |
| 70 | 281.6 | 137.9 | 0.3 | -48.1 | 179.7 | -1.01E-02 |
| 71 | 280.2 | 457.0 | 0.6 | 7.3 | 88.5 | 1.40E-04 |
| 72 | 279.4 | 132.1 | 0.1 | 18.3 | 1.4 | 4.20E-03 |
| 73 | 277.4 | 112.6 | 0.2 | -6.7 | 111.7 | -2.12E-03 |
| 74 | 277.0 | 219.6 | 0.2 | 6.4 | 82.2 | 5.32E-04 |
| 75 | 276.4 | 28.2 | 0.3 | -7.2 | 176.9 | -3.59E-02 |
| 76 | 274.6 | 55.4 | 0.2 | -1.3 | 97.5 | -1.73E-03 |
| 77 | 274.4 | 92.1 | 0.7 | -68.9 | 179.6 | -3.25E-02 |
| 78 | 274.3 | 159.3 | 0.4 | 51.0 | 28.2 | 8.04E-03 |
| 79 | 274.0 | 11.9 | 0.2 | -2.2 | 172.1 | -6.29E-02 |
| 80 | 273.7 | 97.6 | 0.1 | 11.2 | 4.9 | 4.69E-03 |

**S5. NMR characterization**

**Figure S17**. ^1^H NMR spectrum of compound **3** (300 MHz, CDCl_3_).

**Figure S18**. ^13^C NMR spectrum of compound **3** (300 MHz, CDCl_3_).

**Figure S19**. ^1^H NMR spectrum of compound **4** (300 MHz, CDCl_3_).

**Figure S20**. ^13^C NMR spectrum of compound **4** (300 MHz, CDCl_3_).

**Figure S21**. ^1^H NMR spectrum of compound **11** (300 MHz, CD_2_Cl_2_).

**Figure S22**. ^13^C NMR spectrum of compound **11** (300 MHz, CD_2_Cl_2_).

**Figure S23**. ^1^H NMR spectrum of compound **5** (300 MHz, CD_2_Cl_2_).

**Figure S24**. ^13^C NMR spectrum of compound **5** (300 MHz, CD_2_Cl_2_).

**Figure S25**. ^1^H NMR spectrum of compound **6** (300 MHz, CD_2_Cl_2_).

**Figure S26**. ^13^C NMR spectrum of compound **6** (300 MHz, CD_2_Cl_2_).

**Figure S27**. ^1^H NMR spectrum of compound **9** (300 MHz, C_2_D_4_Cl_2_).

**Figure S28**. ^13^C NMR spectrum of compound **9** (300 MHz, C_2_D_4_Cl_2_).

**S6. Supplementary References**

(1) Tanaka, H.; Kato, Y.; Fujiki, M.; Inoue, Y.; Mori, T. Combined Experimental and Theoretical Study on Circular Dichroism and Circularly Polarized Luminescence of Configurationally Robust D3-Symmetric Triple Pentahelicene. *J. Phys. Chem. A* **2018,** *122*, 7378-7384.

(2) Hosokawa, T.; Takahashi, Y.; Matsushima, T.; Watanabe, S.; Kikkawa, S.; Azumaya, I.; Tsurusaki, A.; Kamikawa, K. Synthesis, Structures, and Properties of Hexapole Helicenes: Assembling Six [5]Helicene Substructures into Highly Twisted Aromatic Systems. *J. Am. Chem. Soc.* **2017,** *139*, 18512-18521.

(3) Cruz, C. M.; Márquez, I. R.; Mariz, I. F. A.; Blanco, V.; Sánchez-Sánchez, C.; Sobrado, J. M.; Martín-Gago, J. A.; Cuerva, J. M.; Maçôas, E.; Campaña, A. G. Enantiopure distorted ribbon-shaped nanographene combining two-photon absorption-based upconversion and circularly polarized luminescence. *Chem.Sci.* **2018,** *9*, 3917-3924.

(4) Cruz, C. M.; Márquez, I. R.; Castro-Fernández, S.; Cuerva, J. M.; Maçôas, E.; Campaña, A. G. A Triskelion-Shaped Saddle–Helix Hybrid Nanographene. *Angew. Chem. Int. Ed.* **2019,** *58*, 8068-8072.

(5) Xu, X.; Muñoz-Mármol, R.; Vasylevskyi, S.; Villa, A.; Folpini, G.; Scotognella, F.; Maria Paternò, G.; Narita, A. Synthesis of Bioctacene-Incorporated Nanographene with Near-Infrared Chiroptical Properties. *Angew. Chem. Int. Ed.* **2023,** *62*, e202218350.

(6) Yao, X.; Zheng, W.; Osella, S.; Qiu, Z.; Fu, S.; Schollmeyer, D.; Müller, B.; Beljonne, D.; Bonn, M.; Wang, H. I.; Müllen, K.; Narita, A. Synthesis of Nonplanar Graphene Nanoribbon with Fjord Edges. *J. Am. Chem. Soc.* **2021,** *143*, 5654-5658.

(7) Xu, X.; Serra, G.; Villa, A.; Muñoz-Mármol, R.; Vasylevskyi, S.; Gadea, M.; Lucotti, A.; Lin, Z.; Boj, P. G.; Kabe, R.; Tommasini, M.; Díaz-García, M. Á.; Scotognella, F.; Paternò, G. M.; Narita, A. Synthesis of zigzag- and fjord-edged nanographene with dual amplified spontaneous emission. *Chem.Sci.* **2022,** *13*, 13040-13045.

(8) Castro-Fernández, S.; Cruz, C. M.; Mariz, I. F. A.; Márquez, I. R.; Jiménez, V. G.; Palomino-Ruiz, L.; Cuerva, J. M.; Maçôas, E.; Campaña, A. G. Two-Photon Absorption Enhancement by the Inclusion of a Tropone Ring in Distorted Nanographene Ribbons. *Angew. Chem.* **2020,** *132*, 7205-7211.

(9) Zhang, Z.; Zhu, H.; Gu, J.; Shi, H.; Hirose, T.; Jiang, L.; Zhu, Y.; Zhong, D.; Wang, J. Nonplanar Nanographene with a Large Conjugated π-Surface. *J. Am. Chem. Soc.* **2024,** *146*, 24681-24688.

(10) Lombardi, F.; Ma, J.; Alexandropoulos, D. I.; Komber, H.; Liu, J.; Myers, W. K.; Feng, X.; Bogani, L. Synthetic tuning of the quantum properties of open-shell radicaloids. *Chem* **2021,** *7*, 1363-1378.

(11) Liu, J.; Li, B.-W.; Tan, Y.-Z.; Giannakopoulos, A.; Sanchez-Sanchez, C.; Beljonne, D.; Ruffieux, P.; Fasel, R.; Feng, X.; Müllen, K. Toward Cove-Edged Low Band Gap Graphene Nanoribbons. *J. Am. Chem. Soc.* **2015,** *137*, 6097-6103.

(12) Caivano, I.; Bingel, S.; Císařová, I.; Nečas, D.; Kotora, M. Catalytic approach to unsymmetrical [7]-helical indenofluorenes: Cyclotrimerization vs. dehydro-Diels-Alder reaction pathways. *Catal. Today* **2022,** *390-391*, 48-56.

(13) Ma, J.; Zhang, K.; Schellhammer, K. S.; Fu, Y.; Komber, H.; Xu, C.; Popov, A. A.; Hennersdorf, F.; Weigand, J. J.; Zhou, S.; Pisula, W.; Ortmann, F.; Berger, R.; Liu, J.; Feng, X. Wave-shaped polycyclic hydrocarbons with controlled aromaticity. *Chem.Sci.* **2019,** *10*, 4025-4031.

(14) Dolomanov, O. V.; Bourhis, L. J.; Gildea, R. J.; Howard, J. A. K.; Puschmann, H. OLEX2: a complete structure solution, refinement and analysis program. *J. Appl. Crystallogr.* **2009,** *42*, 339-341.

(15) Sheldrick, G. SHELXT - Integrated space-group and crystal-structure determination. *Acta Crystallographica Section A* **2015,** *71*, 3-8.

(16) Stephens, P. J.; Devlin, F. J.; Chabalowski, C. F.; Frisch, M. J. Ab Initio Calculation of Vibrational Absorption and Circular Dichroism Spectra Using Density Functional Force Fields. *J. Phys. Chem.* **1994,** *98*, 11623-11627.

(17) Weigend, F.; Ahlrichs, R. Balanced basis sets of split valence, triple zeta valence and quadruple zeta valence quality for H to Rn: Design and assessment of accuracy. *Phys. Chem. Chem. Phys.* **2005,** *7*, 3297-3305.

(18) Frisch, M. J.; Trucks, G. W.; Schlegel, H. B.; Scuseria, G. E.; Robb, M. A.; Cheeseman, J. R.; Scalmani, G.; Barone, V.; Petersson, G. A.; Nakatsuji, H.; Li, X.; Caricato, M.; Marenich, A. V.; Bloino, J.; Janesko, B. G.; Gomperts, R.; Mennucci, B.; Hratchian, H. P.; Ortiz, J. V.; Izmaylov, A. F.; Sonnenberg, J. L.; Williams; Ding, F.; Lipparini, F.; Egidi, F.; Goings, J.; Peng, B.; Petrone, A.; Henderson, T.; Ranasinghe, D.; Zakrzewski, V. G.; Gao, J.; Rega, N.; Zheng, G.; Liang, W.; Hada, M.; Ehara, M.; Toyota, K.; Fukuda, R.; Hasegawa, J.; Ishida, M.; Nakajima, T.; Honda, Y.; Kitao, O.; Nakai, H.; Vreven, T.; Throssell, K.; Montgomery Jr., J. A.; Peralta, J. E.; Ogliaro, F.; Bearpark, M. J.; Heyd, J. J.; Brothers, E. N.; Kudin, K. N.; Staroverov, V. N.; Keith, T. A.; Kobayashi, R.; Normand, J.; Raghavachari, K.; Rendell, A. P.; Burant, J. C.; Iyengar, S. S.; Tomasi, J.; Cossi, M.; Millam, J. M.; Klene, M.; Adamo, C.; Cammi, R.; Ochterski, J. W.; Martin, R. L.; Morokuma, K.; Farkas, O.; Foresman, J. B.; Fox, D. J. *Gaussian 16 Rev. A.03*, Wallingford, CT, 2016.

(19) Grimme, S.; Antony, J.; Ehrlich, S.; Krieg, H. A consistent and accurate ab initio parametrization of density functional dispersion correction (DFT-D) for the 94 elements H-Pu. *J. Chem. Phys* **2010,** *132*, 154104.

(20) Grimme, S.; Ehrlich, S.; Goerigk, L. Effect of the damping function in dispersion corrected density functional theory. *J. Comput. Chem.* **2011,** *32*, 1456-1465.

(21) Marenich, A. V.; Cramer, C. J.; Truhlar, D. G. Universal Solvation Model Based on Solute Electron Density and on a Continuum Model of the Solvent Defined by the Bulk Dielectric Constant and Atomic Surface Tensions. *J. Phys. Chem. B* **2009,** *113*, 6378-6396.

(22) Frisch, M. J.; Trucks, G. W.; Schlegel, H. B.; Scuseria, G. E.; Robb, M. A.; Cheeseman, J. R.; Scalmani, G.; Barone, V.; Petersson, G. A.; Nakatsuji, H.; Li, X.; Caricato, M.; Marenich, A. V.; Bloino, J.; Janesko, B. G.; Gomperts, R.; Mennucci, B.; Hratchian, H. P.; Ortiz, J. V.; Izmaylov, A. F.; Sonnenberg, J. L.; Williams; Ding, F.; Lipparini, F.; Egidi, F.; Goings, J.; Peng, B.; Petrone, A.; Henderson, T.; Ranasinghe, D.; Zakrzewski, V. G.; Gao, J.; Rega, N.; Zheng, G.; Liang, W.; Hada, M.; Ehara, M.; Toyota, K.; Fukuda, R.; Hasegawa, J.; Ishida, M.; Nakajima, T.; Honda, Y.; Kitao, O.; Nakai, H.; Vreven, T.; Throssell, K.; Montgomery Jr., J. A.; Peralta, J. E.; Ogliaro, F.; Bearpark, M. J.; Heyd, J. J.; Brothers, E. N.; Kudin, K. N.; Staroverov, V. N.; Keith, T. A.; Kobayashi, R.; Normand, J.; Raghavachari, K.; Rendell, A. P.; Burant, J. C.; Iyengar, S. S.; Tomasi, J.; Cossi, M.; Millam, J. M.; Klene, M.; Adamo, C.; Cammi, R.; Ochterski, J. W.; Martin, R. L.; Morokuma, K.; Farkas, O.; Foresman, J. B.; Fox, D. J. *Gaussian 16 Rev. C.01*, Wallingford, CT, 2016.

(23) Tomasi, J.; Mennucci, B.; Cammi, R. Quantum Mechanical Continuum Solvation Models. *Chem. Rev.* **2005,** *105*, 2999-3094.

(24) Cancès, E.; Mennucci, B.; Tomasi, J. A new integral equation formalism for the polarizable continuum model: Theoretical background and applications to isotropic and anisotropic dielectrics. *J. Chem. Phys* **1997,** *107*, 3032-3041.

(25) Cossi, M.; Barone, V.; Cammi, R.; Tomasi, J. Ab initio study of solvated molecules: a new implementation of the polarizable continuum model. *Chem. Phys. Lett.* **1996,** *255*, 327-335.

(26) Lu, T.; Chen, F. Multiwfn: A multifunctional wavefunction analyzer. *J. Comput. Chem.* **2012,** *33*, 580-592.

(27) Lu, T. A comprehensive electron wavefunction analysis toolbox for chemists, Multiwfn. *J. Chem. Phys* **2024,** *161*, 082503.

(28) Hanwell, M. D.; Curtis, D. E.; Lonie, D. C.; Vandermeersch, T.; Zurek, E.; Hutchison, G. R. Avogadro: an advanced semantic chemical editor, visualization, and analysis platform. *J. Cheminform.* **2012,** *4*, 17.

(29) Humphrey, W.; Dalke, A.; Schulten, K. VMD: Visual Molecular Dynamics. *J. Mol. Graph.* **1996**, 14, 33–38.

**Cartesian coordinates of the optimized structures**

**Mechanistic investigations part:**

| **R1** | | | | | | | |
| --- | --- | --- | --- | --- | --- | --- | --- |
| C | 1.701298 | -0.57908 | 2.209485 | C | 4.082473 | -2.58239 | -0.16002 |
| C | 1.831737 | -1.86828 | 2.816895 | C | 4.702191 | -1.32862 | -0.06204 |
| C | 1.054464 | -0.46709 | 0.910096 | C | 5.42353 | -0.97287 | 1.218573 |
| C | 0.647948 | -1.64489 | 0.225748 | C | 3.577773 | 0.210302 | -3.39488 |
| C | 0.821511 | -2.90915 | 0.871542 | C | 2.765855 | -4.33847 | -1.4253 |
| C | 1.361024 | -3.01635 | 2.118215 | H | 0.553506 | -3.8149 | 0.341623 |
| C | 0.871718 | 0.807252 | 0.248563 | H | 1.485686 | -3.99996 | 2.577277 |
| C | 0.673566 | 0.825469 | -1.10972 | H | 0.632837 | 1.787138 | -1.62699 |
| C | 0.34413 | -0.35052 | -1.845 | H | 0.262267 | 0.665118 | -3.75651 |
| C | 0.127165 | -1.56618 | -1.12833 | H | -0.74233 | -1.28316 | -4.95111 |
| C | 0.057837 | -0.26969 | -3.23036 | H | -1.5003 | -3.24807 | -3.6276 |
| C | -0.51518 | -1.34026 | -3.88449 | H | -0.41901 | -5.109 | -2.44967 |
| C | -0.90578 | -2.47103 | -3.14349 | H | -1.17261 | -7.15573 | -1.25521 |
| C | -0.64108 | -2.58905 | -1.77995 | H | -2.25087 | -6.93046 | 0.98187 |
| C | -1.26777 | -3.73166 | -1.05293 | H | -3.03998 | -5.57491 | 2.795653 |
| C | -1.00779 | -5.00754 | -1.53591 | H | -3.44831 | -3.34509 | 3.818785 |
| C | -1.40145 | -6.17137 | -0.84093 | H | -3.73539 | -0.98771 | 3.581722 |
| C | -2.00447 | -6.04699 | 0.387637 | H | -2.14308 | -0.5214 | 2.976445 |
| C | -2.30922 | -4.76495 | 0.923384 | H | -4.85069 | -0.05924 | 1.600357 |
| C | -2.04024 | -3.57355 | 0.160135 | H | -3.68184 | 1.141969 | 2.128823 |
| C | -2.8489 | -4.65938 | 2.23075 | H | -2.28155 | -1.11914 | -1.13861 |
| C | -3.09035 | -3.42442 | 2.78888 | H | -2.68631 | 2.068231 | 0.029658 |
| C | -2.92238 | -2.2486 | 2.029793 | H | 2.554697 | -3.00288 | 4.509609 |
| C | -2.49268 | -2.313 | 0.697474 | H | 3.527633 | -1.0295 | 5.677036 |
| C | -3.13849 | -0.89206 | 2.662164 | H | 3.381637 | 1.22793 | 4.595902 |
| C | -3.76479 | 0.125183 | 1.711909 | H | 2.258882 | 1.512082 | 2.463727 |
| C | -3.14696 | 0.053757 | 0.341917 | H | -0.99292 | 1.193882 | 1.976302 |
| C | -2.58014 | -1.08519 | -0.09924 | H | -1.80492 | 3.312035 | 3.014985 |
| C | -3.24637 | 1.295278 | -0.52464 | H | -0.69202 | 5.465361 | 2.431549 |
| O | -2.67026 | 1.144392 | -1.80923 | H | 1.054905 | 6.690359 | 1.284199 |
| C | 2.480407 | -2.00469 | 4.070418 | H | 3.157991 | 6.723298 | -0.03264 |
| C | 3.023994 | -0.9123 | 4.714678 | H | 4.809698 | 5.531671 | -1.38204 |
| C | 2.93623 | 0.357787 | 4.108175 | H | 5.408172 | 4.625099 | 0.010631 |
| C | 2.293341 | 0.517363 | 2.894577 | H | 4.394251 | 3.457417 | -2.64562 |
| H | -1.74841 | 0.87397 | -1.69281 | H | 6.019755 | 3.345456 | -1.98124 |
| C | 0.665017 | 2.094007 | 0.978633 | H | 3.172123 | 1.191969 | 0.283204 |
| C | -0.45295 | 2.125582 | 1.802593 | H | -6.64982 | 4.463944 | 0.116467 |
| C | -0.92663 | 3.327447 | 2.366143 | H | -7.7088 | 0.843802 | -1.93565 |
| C | -0.30704 | 4.515955 | 2.0515 | H | -6.14802 | -0.70865 | -2.55807 |
| C | 0.857511 | 4.531831 | 1.238127 | H | -4.4014 | -0.31979 | -2.50101 |
| C | 1.409678 | 3.298366 | 0.740989 | H | -5.1732 | -1.11914 | -1.12834 |
| H | -3.16912 | 4.153757 | -0.11078 | C | 1.500684 | 5.758827 | 0.927018 |
| H | -4.51586 | 4.933916 | 0.746797 | C | 2.669579 | 5.773445 | 0.201578 |
| H | -3.68135 | 3.544057 | 1.464208 | C | 3.289858 | 4.56884 | -0.19988 |
| H | -8.91768 | 4.171228 | -0.5767 | C | 2.691455 | 3.335448 | 0.082396 |
| H | -9.07449 | 3.264764 | -2.10081 | C | 4.670258 | 4.597382 | -0.81578 |
| H | -9.48009 | 2.481835 | -0.56435 | C | 4.960442 | 3.38487 | -1.69696 |
| H | 2.619437 | -2.28565 | -3.20042 | C | 4.552496 | 2.116344 | -0.99161 |
| H | 4.155341 | -3.27333 | 0.683188 | C | 3.466121 | 2.12185 | -0.19573 |
| H | 5.264386 | -1.75159 | 1.977397 | C | 5.404242 | 0.870681 | -1.13139 |
| H | 6.511726 | -0.86558 | 1.072775 | C | -4.68237 | 1.786598 | -0.67269 |
| H | 5.05325 | -0.02833 | 1.646185 | C | -5.04863 | 3.04875 | -0.16122 |
| H | 4.516045 | 0.597942 | -3.81124 | C | -6.37676 | 3.483378 | -0.28382 |
| H | 2.983683 | -0.25413 | -4.19383 | C | -7.35299 | 2.706858 | -0.91005 |
| H | 3.017428 | 1.082262 | -3.02009 | C | -6.96532 | 1.465388 | -1.42725 |
| H | 1.719136 | -4.27967 | -1.75755 | C | -5.65425 | 0.988601 | -1.32262 |
| H | 3.309059 | -4.94071 | -2.17357 | C | -5.32722 | -0.36196 | -1.91351 |
| H | 2.791438 | -4.88437 | -0.47175 | C | -4.05371 | 3.963338 | 0.516629 |
| H | 6.066535 | 0.857742 | -0.24676 | C | -8.77701 | 3.184507 | -1.04188 |
| O | 6.229263 | 1.007119 | -2.28902 | C | 4.617086 | -0.43257 | -1.1482 |
| H | 6.692919 | 0.167912 | -2.40589 | C | 3.825693 | -0.76858 | -2.2735 |
| H | 6.692919 | 0.167912 | -2.40589 | C | 3.229016 | -2.03087 | -2.32914 |
| C | 3.372237 | -2.96648 | -1.29809 |  |  |  |  |
| **INT1** | | | | | | | |
| C | -0.27142 | -0.80011 | 2.475037 | H | 3.424783 | -1.09201 | -0.6822 |
| C | -0.88788 | 0.178199 | 3.324389 | H | -1.39292 | 0.75967 | 5.343033 |
| C | -0.25346 | -0.58012 | 1.031804 | H | -0.3632 | -1.24702 | 6.39805 |
| C | -0.70989 | 0.671074 | 0.528831 | H | 0.745027 | -2.95948 | 4.943755 |
| C | -1.38134 | 1.578233 | 1.408226 | H | 0.810575 | -2.68765 | 2.537337 |
| C | -1.48372 | 1.336277 | 2.745655 | H | 2.488861 | -2.68577 | 0.523781 |
| C | 0.064375 | -1.61177 | 0.059627 | H | 3.126912 | -5.07864 | 0.700108 |
| C | -0.07096 | -1.34646 | -1.28553 | H | 1.390507 | -6.84909 | 0.441076 |
| C | -0.35255 | -0.05044 | -1.79877 | H | -0.92149 | -7.46813 | -0.09453 |
| C | -0.55603 | 1.014118 | -0.87497 | H | -3.22644 | -6.85258 | -0.73654 |
| C | -0.33566 | 0.194275 | -3.1956 | H | -4.74969 | -5.01309 | -1.52576 |
| C | -0.4286 | 1.488114 | -3.66679 | H | -4.96648 | -4.37802 | 0.106618 |
| C | -0.45546 | 2.558363 | -2.75293 | H | -3.84017 | -2.85558 | -2.315 |
| C | -0.48382 | 2.358905 | -1.37319 | H | -5.40121 | -2.55228 | -1.53311 |
| C | -0.33228 | 3.546539 | -0.48434 | H | -2.09905 | -1.85199 | 1.055941 |
| C | -1.22099 | 4.59673 | -0.67619 | H | -3.52315 | -0.2745 | 0.870875 |
| C | -1.29669 | 5.687114 | 0.217198 | H | 7.9605 | -1.93654 | -0.93094 |
| C | -0.53136 | 5.672721 | 1.359644 | H | 7.627088 | 2.152221 | -2.19702 |
| C | 0.388851 | 4.617273 | 1.608564 | H | 5.601979 | 3.172493 | -2.52807 |
| C | 0.594744 | 3.587641 | 0.624765 | C | -6.02203 | 1.378455 | -2.51163 |
| C | 1.068563 | 4.546563 | 2.851887 | C | -6.78368 | 2.178105 | -1.65095 |
| C | 1.882529 | 3.474008 | 3.140795 | C | -6.55951 | 2.069225 | -0.26625 |
| C | 2.170174 | 2.503223 | 2.158442 | C | -5.63592 | 1.176762 | 0.259958 |
| C | 1.62889 | 2.613611 | 0.871373 | C | -5.42746 | 1.110434 | 1.750976 |
| C | 2.994468 | 1.284106 | 2.506857 | C | -4.2244 | -0.25833 | -3.04962 |
| C | 3.864394 | 0.778019 | 1.358551 | C | -7.80186 | 3.146012 | -2.18049 |
| C | 3.154776 | 0.847566 | 0.033317 | H | -1.84056 | 2.469103 | 0.988889 |
| C | 2.155302 | 1.726204 | -0.16939 | H | -2.00509 | 2.042472 | 3.395441 |
| C | 3.653264 | -0.07894 | -1.05961 | H | 0.091106 | -2.15668 | -2.00023 |
| O | 3.002852 | 0.095748 | -2.30537 | H | -0.20071 | -0.6453 | -3.88134 |
| C | -0.91548 | -0.00729 | 4.728624 | H | -0.40086 | 1.691103 | -4.73927 |
| C | -0.34442 | -1.11877 | 5.313802 | H | -0.37638 | 3.580134 | -3.12856 |
| C | 0.273653 | -2.08294 | 4.493859 | H | -1.92955 | 4.539316 | -1.50544 |
| C | 0.303566 | -1.92907 | 3.118985 | H | -2.00195 | 6.498847 | 0.027255 |
| H | 2.050473 | 0.013701 | -2.16136 | H | -0.63099 | 6.463271 | 2.107354 |
| C | 0.387201 | -3.05375 | 0.340448 | H | 0.903371 | 5.336548 | 3.588083 |
| C | 1.713246 | -3.45232 | 0.483715 | H | 2.344433 | 3.383554 | 4.127211 |
| C | 2.076786 | -4.81027 | 0.569372 | H | 3.605817 | 1.476245 | 3.401119 |
| C | 1.114414 | -5.79243 | 0.433933 | H | 2.277438 | 0.489087 | 2.788655 |
| C | -0.24153 | -5.43468 | 0.226791 | H | 4.785934 | 1.383716 | 1.266143 |
| C | -0.6237 | -4.056 | 0.240635 | H | 4.213805 | -0.24603 | 1.567531 |
| C | -1.2241 | -6.41794 | -0.08922 | H | 1.791351 | 1.847362 | -1.18321 |
| C | -2.51448 | -6.07614 | -0.44834 | H | 4.109932 | 2.193518 | -2.64695 |
| C | -2.92395 | -4.72736 | -0.43709 | H | 4.581875 | 2.902213 | -1.09821 |
| C | -2.01445 | -3.74234 | 0.004144 | H | 4.589163 | -2.82027 | -1.21294 |
| C | -4.32885 | -4.3076 | -0.79448 | H | 6.150874 | -3.22781 | -0.47411 |
| C | -4.36748 | -2.87369 | -1.34731 | H | 4.919534 | -2.38357 | 0.478903 |
| C | -3.68606 | -1.94772 | -0.37043 | H | 9.96539 | -0.7275 | -1.42383 |
| C | -2.56041 | -2.4612 | 0.285257 | H | 9.730355 | 0.35932 | -2.81311 |
| C | -4.02442 | -0.63834 | -0.03112 | H | 9.895694 | 1.036317 | -1.18475 |
| C | 5.161576 | -0.00756 | -1.25437 | H | -6.13158 | 1.510306 | -3.59106 |
| C | 5.959147 | -1.13856 | -0.98379 | H | -7.12467 | 2.710289 | 0.414935 |
| C | 7.349738 | -1.05473 | -1.14313 | H | -6.03535 | 1.868698 | 2.261853 |
| C | 7.97381 | 0.117934 | -1.57339 | H | -5.70156 | 0.126048 | 2.162255 |
| C | 7.162375 | 1.225077 | -1.84885 | H | -4.37291 | 1.290283 | 2.018669 |
| C | 5.772466 | 1.188989 | -1.69798 | H | -4.71592 | -1.153 | -3.46335 |
| C | 4.974337 | 2.431075 | -2.01371 | H | -4.02107 | 0.411598 | -3.89713 |
| C | 5.370197 | -2.45586 | -0.52619 | H | -3.25689 | -0.55765 | -2.62776 |
| C | 9.467869 | 0.195586 | -1.7546 | H | -7.71821 | 3.276012 | -3.26817 |
| C | -4.92957 | 0.297453 | -0.63091 | H | -8.8216 | 2.785722 | -1.96063 |
| C | -5.09708 | 0.444322 | -2.04235 | H | -7.70261 | 4.131549 | -1.69913 |

| **TS1** | | | | | | | |
| --- | --- | --- | --- | --- | --- | --- | --- |
| C | -0.00917 | 0.952477 | 3.254881 | C | -2.27573 | -4.17289 | 0.833566 |
| C | 0.061073 | 2.303239 | 3.724433 | C | -1.6334 | -2.98563 | 1.166208 |
| C | -0.14211 | 0.715425 | 1.817795 | C | -3.55278 | -4.13645 | 0.027072 |
| C | -0.38285 | 1.825391 | 0.940825 | C | -3.61096 | -2.87265 | -0.84699 |
| C | -0.43546 | 3.135253 | 1.486202 | C | -3.42994 | -1.64433 | 0.005292 |
| C | -0.16557 | 3.368078 | 2.809068 | C | -2.27455 | -1.68277 | 0.88422 |
| C | -0.08243 | -0.57877 | 1.224171 | C | -4.21203 | -0.52433 | 0.010577 |
| C | -0.69741 | -0.79041 | -0.0441 | C | 4.024165 | -1.67376 | -1.87938 |
| C | -0.97577 | 0.325091 | -0.91859 | C | 4.520515 | -2.93508 | -1.48195 |
| C | -0.59575 | 1.619929 | -0.49063 | C | 5.66965 | -3.4455 | -2.10136 |
| C | -1.39159 | 0.093683 | -2.23965 | C | 6.338817 | -2.74758 | -3.1097 |
| C | -1.345 | 1.124432 | -3.16607 | C | 5.821339 | -1.50608 | -3.4994 |
| C | -0.77184 | 2.351077 | -2.80229 | C | 4.681719 | -0.95303 | -2.90695 |
| C | -0.34745 | 2.607111 | -1.4955 | C | 4.201802 | 0.39775 | -3.3852 |
| C | 0.456138 | 3.829836 | -1.23223 | C | 3.856882 | -3.76072 | -0.40208 |
| C | -0.08474 | 5.047852 | -1.62259 | C | 7.57449 | -3.30464 | -3.76731 |
| C | 0.509107 | 6.277649 | -1.26772 | C | -5.43877 | -0.22298 | -0.72402 |
| C | 1.610223 | 6.275193 | -0.44455 | C | -6.55217 | -1.1011 | -0.78468 |
| C | 2.201513 | 5.054224 | -0.01599 | C | -7.66898 | -0.72719 | -1.54259 |
| C | 1.701298 | 3.793696 | -0.49724 | C | -7.73504 | 0.486942 | -2.22986 |
| C | 3.260491 | 5.07892 | 0.927917 | C | -6.65397 | 1.37343 | -2.10166 |
| C | 3.786686 | 3.904621 | 1.41564 | C | -5.52607 | 1.058067 | -1.34893 |
| C | 3.37914 | 2.661377 | 0.888779 | C | -4.41054 | 2.064781 | -1.2245 |
| C | 2.422186 | 2.593977 | -0.13361 | C | -6.64453 | -2.37668 | 0.017808 |
| C | 3.921632 | 1.378337 | 1.476586 | C | -8.93042 | 0.853537 | -3.06669 |
| C | 4.118842 | 0.271499 | 0.446377 | H | -0.67973 | 3.971806 | 0.838449 |
| C | 2.975948 | 0.207739 | -0.52994 | H | -0.16866 | 4.392228 | 3.189241 |
| C | 2.232885 | 1.299123 | -0.80051 | H | -0.40858 | -1.69236 | -0.58363 |
| C | 2.767737 | -1.11836 | -1.2212 | H | -1.68941 | -0.91215 | -2.53981 |
| O | 1.668801 | -1.04565 | -2.13138 | H | -1.66354 | 0.95705 | -4.19693 |
| C | 0.247035 | 2.576388 | 5.103173 | H | -0.57964 | 3.102644 | -3.56997 |
| C | 0.30219 | 1.551581 | 6.025666 | H | -1.03566 | 5.051517 | -2.15981 |
| C | 0.130682 | 0.224007 | 5.585837 | H | 0.058349 | 7.216035 | -1.59664 |
| C | -0.02686 | -0.06646 | 4.24057 | H | 2.042469 | 7.21383 | -0.0899 |
| H | 1.848171 | -1.67429 | -2.84227 | H | 3.625229 | 6.044776 | 1.284574 |
| C | 0.455846 | -1.80609 | 1.846473 | H | 4.555737 | 3.926373 | 2.191774 |
| C | 1.726593 | -1.85602 | 2.400196 | H | 4.855432 | 1.571757 | 2.024599 |
| C | 2.25684 | -3.0749 | 2.873311 | H | 3.185477 | 1.042012 | 2.233826 |
| C | 1.538161 | -4.24768 | 2.749173 | H | 5.04393 | 0.443223 | -0.13654 |
| C | 0.245389 | -4.24587 | 2.15962 | H | 4.268861 | -0.69969 | 0.944654 |
| C | -0.32263 | -3.00539 | 1.745211 | H | 1.535107 | 1.221236 | -1.62467 |
| C | -0.49188 | -5.44116 | 1.913074 | H | 2.48924 | -1.81117 | -0.40819 |
| C | -1.69112 | -5.40437 | 1.239815 | H | 0.317258 | 3.618454 | 5.423321 |
| H | 0.440888 | 1.766352 | 7.087075 | H | 4.449262 | 1.191635 | -2.66225 |
| H | 0.112033 | -0.59072 | 6.312985 | H | 2.790433 | -3.94528 | -0.60899 |
| H | -0.18323 | -1.10164 | 3.956581 | H | 4.348454 | -4.7389 | -0.30806 |
| H | 2.317358 | -0.94235 | 2.460848 | H | 3.905416 | -3.27324 | 0.584049 |
| H | 3.254441 | -3.08797 | 3.316639 | H | 7.82847 | -4.30048 | -3.37715 |
| H | 1.963904 | -5.1961 | 3.08435 | H | 7.440156 | -3.39007 | -4.8579 |
| H | -0.06496 | -6.39657 | 2.226489 | H | 8.44199 | -2.64459 | -3.60222 |
| H | -2.21283 | -6.33577 | 1.006718 | H | -8.52874 | -1.40215 | -1.57437 |
| H | -3.61087 | -5.03922 | -0.60015 | H | -6.70114 | 2.345835 | -2.5995 |
| H | -4.42888 | -4.16984 | 0.697181 | H | -4.66045 | 2.991159 | -1.75925 |
| H | -2.77707 | -2.91799 | -1.57225 | H | -4.22077 | 2.332197 | -0.17103 |
| H | -4.5334 | -2.82139 | -1.43401 | H | -3.46407 | 1.682454 | -1.63107 |
| H | -2.34447 | -1.00551 | 1.738194 | H | -5.91309 | -2.40863 | 0.834821 |
| H | -3.82218 | 0.304697 | 0.612504 | H | -7.64772 | -2.46798 | 0.460167 |
| H | 6.048591 | -4.42196 | -1.7881 | H | -6.49447 | -3.27195 | -0.6073 |
| H | 6.322436 | -0.94646 | -4.29454 | H | -9.76642 | 0.157435 | -2.91007 |
| H | 4.678425 | 0.658544 | -4.34062 | H | -9.28083 | 1.87237 | -2.83787 |
| H | 3.112654 | 0.422944 | -3.51646 | H | -8.67483 | 0.835839 | -4.13985 |
| **INT2** | | | | | | | |
| C | 0.366912 | 1.845689 | 3.116381 | C | 4.208991 | 1.034437 | 1.015162 |
| C | 0.686342 | 3.228536 | 3.288844 | C | 4.029475 | -0.27853 | 0.258793 |
| C | 0.111168 | 1.347438 | 1.760079 | C | 2.807561 | -0.27312 | -0.61629 |
| C | -0.02962 | 2.28625 | 0.677725 | C | 2.267853 | 0.881128 | -1.05879 |
| C | 0.186519 | 3.662513 | 0.935474 | C | 2.288241 | -1.6304 | -1.02424 |
| C | 0.588962 | 4.10341 | 2.171264 | O | 1.134906 | -1.49303 | -1.85915 |
| C | -0.04675 | -0.01919 | 1.445748 | C | 0.979016 | 3.743437 | 4.576804 |
| C | -0.90739 | -0.41525 | 0.292974 | C | 0.892796 | 2.936827 | 5.69381 |
| C | -1.0867 | 0.608584 | -0.7756 | C | 0.468712 | 1.601506 | 5.544829 |
| C | -0.39857 | 1.836955 | -0.66384 | C | 0.20771 | 1.072302 | 4.290887 |
| C | -1.66804 | 0.235232 | -1.99128 | H | 1.103391 | -2.2691 | -2.43369 |
| C | -1.49872 | 1.031119 | -3.11857 | C | 0.446847 | -1.15174 | 2.208174 |
| C | -0.6586 | 2.147315 | -3.06008 | C | 1.69214 | -1.13449 | 2.842191 |
| C | -0.06422 | 2.545222 | -1.86009 | C | 2.211688 | -2.2898 | 3.445146 |
| C | 0.98187 | 3.599623 | -1.8961 | C | 1.501449 | -3.47944 | 3.401766 |
| C | 0.651137 | 4.806888 | -2.49835 | C | 0.244094 | -3.55792 | 2.751232 |
| C | 1.503274 | 5.929603 | -2.44178 | C | -0.30743 | -2.37737 | 2.166809 |
| C | 2.662061 | 5.85252 | -1.70619 | C | -0.48201 | -4.77556 | 2.633295 |
| C | 3.047327 | 4.638207 | -1.0734 | C | -1.66883 | -4.81002 | 1.93923 |
| C | 2.262882 | 3.443923 | -1.24454 | C | -2.23786 | -3.63329 | 1.374974 |
| C | 4.19248 | 4.616684 | -0.23591 | C | -1.58662 | -2.41921 | 1.544674 |
| C | 4.541575 | 3.470284 | 0.439607 | C | -3.50581 | -3.71242 | 0.561581 |
| C | 3.844682 | 2.264591 | 0.216075 | C | -3.59486 | -2.56379 | -0.45745 |
| C | 2.774286 | 2.209509 | -0.68776 | C | -3.41888 | -1.23808 | 0.228627 |
| C | -2.14372 | -1.10494 | 1.02663 | H | 1.240052 | 4.799926 | 4.67087 |
| C | -4.28857 | -0.20679 | 0.205197 | H | 1.111132 | 3.337062 | 6.685734 |
| C | 3.336535 | -2.5432 | -1.64551 | H | 0.332853 | 0.973675 | 6.428079 |
| C | 3.588707 | -3.80611 | -1.06514 | H | -0.14007 | 0.045142 | 4.225959 |
| C | 4.541059 | -4.65126 | -1.65067 | H | 2.276926 | -0.2163 | 2.838126 |
| C | 5.245797 | -4.28706 | -2.8007 | H | 3.190315 | -2.25181 | 3.927075 |
| C | 4.966555 | -3.03881 | -3.37084 | H | 1.916707 | -4.38343 | 3.853609 |
| C | 4.029103 | -2.15917 | -2.82049 | H | -0.07111 | -5.68442 | 3.078254 |
| C | 3.79457 | -0.8324 | -3.50448 | H | -2.19722 | -5.7582 | 1.812775 |
| C | 2.865324 | -4.28299 | 0.174646 | H | -3.54804 | -4.68615 | 0.048747 |
| C | 6.26956 | -5.20189 | -3.42055 | H | -4.3792 | -3.68162 | 1.232867 |
| C | -5.5879 | -0.08675 | -0.48936 | H | -2.77878 | -2.70249 | -1.1922 |
| C | -6.65284 | -0.99848 | -0.3049 | H | -4.5365 | -2.5975 | -1.01798 |
| C | -7.84308 | -0.81467 | -1.02514 | H | -2.37892 | -0.47197 | 1.898007 |
| C | -8.0263 | 0.252142 | -1.90509 | H | -3.9604 | 0.703385 | 0.723989 |
| C | -6.9854 | 1.184984 | -2.02405 | H | 4.732072 | -5.62674 | -1.19563 |
| C | -5.78707 | 1.047664 | -1.32489 | H | 5.496076 | -2.73861 | -4.2796 |
| C | -4.72599 | 2.111477 | -1.45428 | H | 4.229113 | -0.83935 | -4.51389 |
| C | -6.60355 | -2.12846 | 0.694372 | H | 2.725837 | -0.59609 | -3.58475 |
| C | -9.30006 | 0.417212 | -2.69159 | H | 4.260062 | -0.00288 | -2.94843 |
| H | 0.045917 | 4.382518 | 0.134921 | H | 1.769835 | -4.25424 | 0.0608 |
| H | 0.789189 | 5.166762 | 2.323197 | H | 3.143936 | -5.31983 | 0.408507 |
| H | -0.39802 | -1.23218 | -0.2461 | H | 3.107689 | -3.6709 | 1.057108 |
| H | -2.19998 | -0.71133 | -2.06969 | H | 6.342923 | -6.15579 | -2.87923 |
| H | -1.94786 | 0.736461 | -4.06928 | H | 6.019464 | -5.42415 | -4.47063 |
| H | -0.39971 | 2.679054 | -3.97712 | H | 7.268097 | -4.73464 | -3.42116 |
| H | -0.32918 | 4.906286 | -2.96918 | H | -8.66153 | -1.52391 | -0.87191 |
| H | 1.210918 | 6.861264 | -2.93003 | H | -7.11901 | 2.052797 | -2.67632 |
| H | 3.303176 | 6.728453 | -1.58277 | H | -5.04158 | 2.89778 | -2.15409 |
| H | 4.77317 | 5.533932 | -0.11527 | H | -4.52162 | 2.59519 | -0.48333 |
| H | 5.38927 | 3.474233 | 1.129256 | H | -3.76973 | 1.702384 | -1.80741 |
| H | 5.234855 | 1.120162 | 1.402298 | H | -5.83007 | -1.96941 | 1.456716 |
| H | 3.550455 | 1.036573 | 1.904874 | H | -7.57242 | -2.22345 | 1.206965 |
| H | 4.900877 | -0.47127 | -0.39563 | H | -6.40754 | -3.09964 | 0.210447 |
| H | 4.00465 | -1.12757 | 0.961155 | H | -10.0835 | -0.27177 | -2.34483 |
| H | 1.494288 | 0.800327 | -1.81241 | H | -9.68805 | 1.445282 | -2.61453 |
| H | 1.967533 | -2.09928 | -0.07733 | H | -9.12919 | 0.21785 | -3.7632 |
| **INT2’** | | | | | | | |
| C | 0.681399 | -0.1523 | -3.55794 | C | -6.36401 | -0.40713 | 3.029634 |
| C | 1.131356 | -1.35276 | -4.19521 | C | -7.31932 | -1.10043 | 2.285887 |
| C | 0.288283 | -0.21035 | -2.1477 | C | -7.00197 | -1.4139 | 0.959096 |
| C | 0.094188 | -1.49704 | -1.5323 | C | -5.77568 | -1.0658 | 0.387735 |
| C | 0.414336 | -2.66951 | -2.27663 | C | -5.52813 | -1.42081 | -1.06076 |
| C | 0.978996 | -2.59661 | -3.5184 | C | -4.18184 | 0.705784 | 3.43389 |
| C | 0.07443 | 0.94702 | -1.33926 | C | -8.65031 | -1.48983 | 2.873596 |
| C | -0.51504 | 0.812007 | -0.075 | H | 0.191453 | -3.64584 | -1.86182 |
| C | -1.00767 | -0.40174 | 0.38241 | H | 1.250292 | -3.51458 | -4.04488 |
| C | -0.51754 | -1.59508 | -0.2231 | H | -0.69348 | 1.715718 | 0.50587 |
| C | -2.14115 | -0.40738 | 1.343688 | H | -2.03272 | 0.381461 | 2.098997 |
| C | -2.33242 | -1.72106 | 1.991055 | H | -3.07853 | -1.81393 | 2.779907 |
| C | -1.58699 | -2.80017 | 1.64421 | H | -1.68596 | -3.71507 | 2.228607 |
| C | -0.59978 | -2.77659 | 0.592222 | H | -1.38958 | -5.2427 | 0.879646 |
| C | 0.285344 | -3.93836 | 0.485112 | H | -0.21019 | -7.33853 | 0.354007 |
| C | -0.33444 | -5.19375 | 0.609723 | H | 2.090997 | -7.24455 | -0.59537 |
| C | 0.31711 | -6.38717 | 0.266516 | H | 4.00322 | -5.99468 | -1.3557 |
| C | 1.594644 | -6.33246 | -0.25522 | H | 5.122009 | -3.81005 | -1.78226 |
| C | 2.285498 | -5.09994 | -0.38061 | H | 5.299409 | -1.44259 | -1.35778 |
| C | 1.686166 | -3.88383 | 0.101834 | H | 3.689514 | -0.93422 | -1.87422 |
| C | 3.555018 | -5.06099 | -1.00973 | H | 4.997712 | -0.47395 | 0.861322 |
| C | 4.185882 | -3.85243 | -1.22052 | H | 4.35209 | 0.710938 | -0.2532 |
| C | 3.654685 | -2.66557 | -0.68193 | H | 1.293514 | -1.56023 | 1.545313 |
| C | 2.482948 | -2.6958 | 0.097823 | H | 2.242361 | 1.584016 | 0.912903 |
| C | 4.262981 | -1.32371 | -1.00962 | H | 1.962807 | -2.25623 | -5.97674 |
| C | 4.184945 | -0.30829 | 0.129614 | H | 1.929275 | -0.1438 | -7.29944 |
| C | 2.89153 | -0.38768 | 0.890544 | H | 0.922249 | 1.911246 | -6.28446 |
| C | 2.137969 | -1.50509 | 0.866199 | H | 0.141772 | 1.913071 | -3.98622 |
| C | 2.490572 | 0.836797 | 1.688687 | H | 2.396819 | 1.990302 | -2.04409 |
| O | 1.350665 | 0.631407 | 2.507544 | H | 2.946809 | 4.360359 | -2.59862 |
| C | 1.604757 | -1.32544 | -5.53074 | H | 1.152197 | 6.081047 | -2.54054 |
| C | 1.571629 | -0.15915 | -6.26793 | H | -1.11081 | 6.743666 | -2.04036 |
| C | 1.02021 | 1.000759 | -5.68946 | H | -3.35811 | 6.211388 | -1.12975 |
| C | 0.586078 | 1.003899 | -4.37424 | H | -4.7455 | 4.581977 | 0.088099 |
| H | 0.621544 | 0.39031 | 1.920667 | H | -5.03728 | 3.332618 | -1.13816 |
| C | 0.308496 | 2.351276 | -1.7587 | H | -3.3751 | 2.971061 | 1.423763 |
| C | 1.606468 | 2.743658 | -2.05275 | H | -4.9813 | 2.287211 | 1.115897 |
| C | 1.918093 | 4.081842 | -2.3625 | H | -2.30559 | 0.851628 | -1.79213 |
| C | 0.922241 | 5.033373 | -2.33319 | H | -3.39686 | -0.68184 | -0.39427 |
| C | -0.41432 | 4.690727 | -1.9918 | H | 5.489111 | 4.256416 | 2.820803 |
| C | -0.76562 | 3.317414 | -1.74134 | H | 5.722533 | 0.615514 | 5.086157 |
| C | -1.39169 | 5.709922 | -1.82715 | H | 4.194658 | -1.08848 | 4.826422 |
| C | -2.64116 | 5.412563 | -1.33402 | H | 2.666214 | -0.80063 | 3.947149 |
| C | -3.00871 | 4.0719 | -1.0778 | H | 4.066199 | -1.47041 | 3.097944 |
| C | -2.13819 | 3.027028 | -1.39119 | H | 2.379121 | 3.710048 | 1.320748 |
| C | -4.30373 | 3.699729 | -0.39704 | H | 3.90767 | 4.603855 | 1.212443 |
| C | -4.0427 | 2.584974 | 0.62804 | H | 3.626834 | 3.19499 | 0.176738 |
| C | -3.42397 | 1.396425 | -0.07017 | H | 7.203181 | 4.048466 | 4.479453 |
| C | -2.64476 | 1.662458 | -1.1516 | H | 6.559523 | 3.215927 | 5.913881 |
| C | -3.5093 | -0.01215 | 0.463011 | H | 7.74155 | 2.397691 | 4.879156 |
| C | 3.625002 | 1.414984 | 2.519475 | H | -6.5935 | -0.13486 | 4.063336 |
| C | 4.081586 | 2.72401 | 2.26222 | H | -7.73695 | -1.94229 | 0.345582 |
| C | 5.138433 | 3.241153 | 3.024702 | H | -6.41789 | -1.88992 | -1.50188 |
| C | 5.747801 | 2.501766 | 4.041156 | H | -5.28489 | -0.5324 | -1.6656 |
| C | 5.268241 | 1.209143 | 4.287516 | H | -4.69226 | -2.13159 | -1.17754 |
| C | 4.220733 | 0.650451 | 3.5489 | H | -4.75461 | 1.186601 | 4.238818 |
| C | 3.760481 | -0.75045 | 3.875011 | H | -3.46371 | 0.028673 | 3.926571 |
| C | 3.467394 | 3.597353 | 1.190656 | H | -3.60234 | 1.492464 | 2.938278 |
| C | 6.871728 | 3.073957 | 4.865708 | H | -8.74384 | -1.16305 | 3.918909 |
| C | -4.80712 | -0.37837 | 1.165025 | H | -9.48115 | -1.04424 | 2.302633 |
| C | -5.11747 | -0.03437 | 2.501195 | H | -8.79172 | -2.58264 | 2.845215 |
| **INT3** | | | | | | | |
| C | 1.218676 | 2.972279 | 2.524959 | C | 2.05221 | -1.21805 | 3.906879 |
| C | 2.039784 | 4.110134 | 2.24665 | C | 1.301839 | -2.37202 | 3.9998 |
| C | 0.702691 | 2.182907 | 1.422713 | C | 0.051388 | -2.45324 | 3.336682 |
| C | 0.833937 | 2.672756 | 0.106433 | C | -0.4491 | -1.31551 | 2.628635 |
| C | 1.601638 | 3.856662 | -0.12685 | C | -0.69708 | -3.66387 | 3.305333 |
| C | 2.226197 | 4.516186 | 0.893497 | C | -1.84282 | -3.75991 | 2.554236 |
| C | -0.06773 | 0.965759 | 1.622787 | C | -2.34722 | -2.641 | 1.833664 |
| C | -1.11626 | 0.717957 | 0.741126 | C | -1.6791 | -1.42751 | 1.914758 |
| C | -1.00433 | 1.192019 | -0.61791 | C | -3.60409 | -2.78839 | 1.010954 |
| C | 0.138977 | 1.985505 | -0.9679 | C | -3.72216 | -1.69232 | -0.05481 |
| C | -1.81342 | 0.68808 | -1.66872 | C | -3.54086 | -0.33729 | 0.572532 |
| C | -1.44627 | 0.837141 | -2.99212 | C | -2.24172 | -0.14486 | 1.335686 |
| C | -0.22485 | 1.44383 | -3.31576 | C | -4.44064 | 0.663207 | 0.541385 |
| C | 0.584233 | 1.995652 | -2.32441 | C | 1.971423 | -3.55722 | -0.85562 |
| C | 1.92877 | 2.501212 | -2.72388 | C | 2.332559 | -4.70227 | -0.11326 |
| C | 1.947236 | 3.477127 | -3.71226 | C | 2.75881 | -5.85729 | -0.78578 |
| C | 3.127494 | 4.153163 | -4.08515 | C | 2.831674 | -5.91701 | -2.17803 |
| C | 4.293121 | 3.878187 | -3.41262 | C | 2.460185 | -4.77569 | -2.89749 |
| C | 4.336233 | 2.875077 | -2.40534 | C | 2.037642 | -3.59758 | -2.27157 |
| C | 3.164924 | 2.093804 | -2.09158 | C | 1.674772 | -2.41891 | -3.14412 |
| C | 5.545236 | 2.663955 | -1.69545 | C | 2.261419 | -4.7425 | 1.39648 |
| C | 5.612141 | 1.721191 | -0.69715 | C | 3.290548 | -7.16267 | -2.89277 |
| C | 4.511332 | 0.883491 | -0.42836 | C | -5.73582 | 0.710919 | -0.17885 |
| C | 3.314205 | 1.005449 | -1.1491 | C | -6.81114 | -0.15523 | 0.113331 |
| C | 4.595664 | -0.13863 | 0.681273 | C | -8.00029 | -0.04662 | -0.62583 |
| C | 3.90563 | -1.45034 | 0.327128 | C | -8.16514 | 0.902686 | -1.63408 |
| C | 2.548015 | -1.18211 | -0.26476 | C | -7.10457 | 1.787328 | -1.87853 |
| C | 2.302647 | -0.03997 | -0.93493 | C | -5.90682 | 1.718141 | -1.16645 |
| C | 1.522577 | -2.28342 | -0.13069 | C | -4.81061 | 2.71664 | -1.44229 |
| O | 0.247973 | -1.8302 | -0.56591 | C | -6.7536 | -1.17407 | 1.225033 |
| C | 2.578663 | 4.868493 | 3.319165 | H | 4.091246 | 0.289427 | 1.568444 |
| C | 2.271628 | 4.566815 | 4.629628 | H | 4.505732 | -2.02787 | -0.40235 |
| C | 1.382236 | 3.504527 | 4.907967 | H | 3.821197 | -2.08363 | 1.223792 |
| C | 0.867816 | 2.735399 | 3.882931 | H | 1.33456 | 0.047114 | -1.40402 |
| H | -0.41874 | -2.39258 | -0.1519 | H | 1.463702 | -2.50484 | 0.945958 |
| C | 0.323536 | -0.1033 | 2.567633 | H | 3.224009 | 5.718143 | 3.081395 |
| C | 1.575051 | -0.10642 | 3.186931 | H | 2.685886 | 5.162996 | 5.446122 |
| C | 2.05221 | -1.21805 | 3.906879 | H | 1.089639 | 3.295246 | 5.939642 |
| C | 1.301839 | -2.37202 | 3.9998 | H | 0.169906 | 1.935047 | 4.121414 |
| C | 0.051388 | -2.45324 | 3.336682 | H | 2.22364 | 0.760217 | 3.089533 |
| C | -0.4491 | -1.31551 | 2.628635 | H | 3.035788 | -1.1601 | 4.378807 |
| C | -0.69708 | -3.66387 | 3.305333 | H | 1.671397 | -3.24209 | 4.547315 |
| C | -1.84282 | -3.75991 | 2.554236 | H | -0.32335 | -4.52722 | 3.861007 |
| C | -2.34722 | -2.641 | 1.833664 | H | -2.38669 | -4.70695 | 2.501865 |
| C | -1.6791 | -1.42751 | 1.914758 | H | -3.61688 | -3.78398 | 0.537587 |
| C | -3.60409 | -2.78839 | 1.010954 | H | -4.48268 | -2.75604 | 1.67638 |
| C | -3.72216 | -1.69232 | -0.05481 | H | -2.91905 | -1.85246 | -0.79724 |
| C | -3.54086 | -0.33729 | 0.572532 | H | -4.67643 | -1.75863 | -0.59283 |
| C | -2.24172 | -0.14486 | 1.335686 | H | -2.53766 | 0.43928 | 2.22603 |
| C | -4.44064 | 0.663207 | 0.541385 | H | -4.14208 | 1.600231 | 1.029721 |
| C | 1.971423 | -3.55722 | -0.85562 | H | 3.035143 | -6.73737 | -0.19799 |
| C | 2.332559 | -4.70227 | -0.11326 | H | 2.501405 | -4.79897 | -3.99083 |
| C | 1.522577 | -2.28342 | -0.13069 | H | 1.586262 | -2.7334 | -4.19418 |
| O | 0.247973 | -1.8302 | -0.56591 | H | 0.733085 | -1.9555 | -2.82573 |
| C | 2.578663 | 4.868493 | 3.319165 | H | 2.444921 | -1.63349 | -3.09205 |
| C | 2.271628 | 4.566815 | 4.629628 | H | 1.24318 | -4.54296 | 1.761862 |
| C | 1.382236 | 3.504527 | 4.907967 | H | 2.560513 | -5.73255 | 1.769171 |
| C | 0.867816 | 2.735399 | 3.882931 | H | 2.917931 | -3.99812 | 1.872455 |
| H | -0.41874 | -2.39258 | -0.1519 | H | 3.512587 | -7.9761 | -2.18647 |
| C | 0.323536 | -0.1033 | 2.567633 | H | 2.523529 | -7.52528 | -3.59709 |
| C | 1.575051 | -0.10642 | 3.186931 | H | 4.201967 | -6.97062 | -3.48348 |
| C | -9.43898 | 0.990573 | -2.43534 | H | -8.82777 | -0.72255 | -0.38961 |
| H | 1.675941 | 4.249804 | -1.13674 | H | -7.21799 | 2.559126 | -2.64582 |
| H | 2.823391 | 5.407688 | 0.68806 | H | -5.0987 | 3.406782 | -2.24807 |
| H | -2.71156 | 0.131571 | -1.43747 | H | -4.58459 | 3.322438 | -0.54833 |
| H | -2.07546 | 0.417491 | -3.78025 | H | -3.86811 | 2.228468 | -1.72987 |
| H | 0.132163 | 1.438446 | -4.34724 | H | -5.97353 | -0.93431 | 1.959296 |
| H | 0.998144 | 3.773651 | -4.16358 | H | -7.71995 | -1.22625 | 1.749475 |
| H | 3.095514 | 4.919147 | -4.86324 | H | -6.5465 | -2.18636 | 0.839351 |
| H | 5.210434 | 4.4296 | -3.63364 | H | -10.2248 | 0.343344 | -2.01888 |
| H | 6.414538 | 3.276706 | -1.94583 | H | -9.82558 | 2.022257 | -2.46305 |
| H | 6.532627 | 1.589077 | -0.12236 | H | -9.2717 | 0.682746 | -3.48174 |
| H | 5.645194 | -0.30855 | 0.965521 |  |  |  |  |
| **INT4** | | | | | | | |
| C | 2.159413 | 2.783934 | -2.65053 | C | -6.96317 | -3.28268 | -2.23191 |
| C | 3.510615 | 2.793249 | -3.111 | C | -7.03351 | -1.8977 | -2.43512 |
| C | 1.68796 | 1.67543 | -1.83758 | C | -5.90031 | -1.08417 | -2.36413 |
| C | 2.439161 | 0.473246 | -1.82535 | C | -6.01485 | 0.402432 | -2.59296 |
| C | 3.795153 | 0.528232 | -2.29211 | C | -3.22589 | -3.72783 | -1.59993 |
| C | 4.328389 | 1.656829 | -2.84725 | C | -8.20163 | -4.13614 | -2.3285 |
| C | 0.420113 | 1.724975 | -1.13051 | H | 4.423586 | -0.34941 | -2.21792 |
| C | -0.31256 | 0.543237 | -1.02959 | H | 5.370688 | 1.666417 | -3.17438 |
| C | 0.371287 | -0.72187 | -1.15226 | H | -1.37889 | -1.91213 | -0.73972 |
| C | 1.794412 | -0.75965 | -1.39801 | H | -0.20035 | -4.05953 | -0.59708 |
| C | -0.30797 | -1.9342 | -0.88267 | H | 2.284135 | -4.11331 | -0.83624 |
| C | 0.353919 | -3.14102 | -0.79954 | H | 4.026686 | -2.78606 | -3.28277 |
| C | 1.744358 | -3.1711 | -0.9507 | H | 6.386117 | -3.56659 | -3.24423 |
| C | 2.47234 | -2.0114 | -1.21961 | H | 7.614679 | -3.69432 | -1.07968 |
| C | 3.957229 | -2.26196 | -1.20607 | H | 7.585277 | -3.5206 | 1.366973 |
| C | 4.58913 | -2.75411 | -2.34712 | H | 6.390023 | -3.0655 | 3.482086 |
| C | 5.919903 | -3.21388 | -2.3222 | H | 4.135313 | -2.65363 | 4.547149 |
| C | 6.607225 | -3.27498 | -1.12491 | H | 4.275752 | -0.93292 | 4.181181 |
| C | 5.982944 | -2.84505 | 0.072131 | H | 2.015945 | -2.81128 | 3.272498 |
| C | 4.677212 | -2.26266 | 0.02447 | H | 1.863408 | -1.5006 | 4.460031 |
| C | 6.587585 | -3.07499 | 1.341924 | H | 2.896722 | -0.18862 | 0.527577 |
| C | 5.922359 | -2.8189 | 2.526416 | H | 5.032504 | 3.881674 | -4.20253 |
| C | 4.644988 | -2.22398 | 2.512477 | H | 3.509993 | 5.732437 | -4.87002 |
| C | 4.084532 | -1.83892 | 1.272206 | H | 1.088753 | 5.623761 | -4.24587 |
| C | 3.903466 | -1.89604 | 3.783826 | H | 0.242967 | 3.792349 | -2.85368 |
| C | 2.388627 | -1.81061 | 3.546904 | H | 1.771596 | 4.07169 | -0.65156 |
| C | 2.132576 | -0.83859 | 2.423027 | H | 1.05114 | 6.003463 | 0.663399 |
| C | 3.016225 | -0.90612 | 1.335454 | H | -1.15937 | 5.997338 | 1.816489 |
| C | 1.140874 | 0.131631 | 2.364491 | H | -3.19569 | 4.765951 | 2.483066 |
| C | 3.987328 | 3.883367 | -3.88393 | H | -4.52428 | 2.673174 | 2.472319 |
| C | 3.139592 | 4.903703 | -4.26281 | H | -4.29394 | 0.171838 | 2.184044 |
| C | 1.775667 | 4.8497 | -3.89696 | H | -5.01706 | 0.497205 | 0.605598 |
| C | 1.300238 | 3.818741 | -3.11127 | H | -2.42235 | -1.03523 | 1.143817 |
| C | -0.05403 | 2.945544 | -0.42801 | H | -3.93737 | -1.69097 | 0.468414 |
| C | 0.776566 | 4.049327 | -0.21648 | H | -2.06828 | 1.297967 | -1.81332 |
| C | 0.374862 | 5.152173 | 0.561455 | H | -3.15701 | -0.28239 | -2.92621 |
| C | -0.84515 | 5.148734 | 1.204243 | H | -1.76063 | 2.864776 | 4.637747 |
| C | -1.68681 | 4.012364 | 1.11552 | H | -2.79399 | -1.3008 | 4.387689 |
| C | -1.30604 | 2.914042 | 0.278828 | H | -1.66747 | -2.68602 | 2.846052 |
| C | -2.88934 | 3.916425 | 1.868052 | H | -0.13924 | -2.23282 | 2.06364 |
| C | -3.62582 | 2.758306 | 1.855411 | H | -0.15763 | -2.78748 | 3.753805 |
| C | -3.25019 | 1.651241 | 1.047007 | H | 0.798072 | 2.875596 | 2.235113 |
| C | -2.14366 | 1.764713 | 0.215023 | H | 0.16704 | 3.722039 | 3.653291 |
| C | -4.0522 | 0.375521 | 1.127915 | H | 1.549613 | 2.611786 | 3.817935 |
| C | -3.29416 | -0.8032 | 0.511069 | H | -4.28154 | 1.849466 | 4.836938 |
| C | -2.84092 | -0.38209 | -0.86028 | H | -4.29761 | 0.187345 | 5.488048 |
| C | -1.83294 | 0.753845 | -0.87987 | H | -3.46335 | 1.496304 | 6.364534 |
| C | -3.46884 | -0.76003 | -1.98732 | H | -5.631 | -4.9215 | -1.81212 |
| C | -0.03526 | 0.300412 | 3.174866 | H | -8.00114 | -1.43813 | -2.65698 |
| C | -0.36321 | 1.634083 | 3.582171 | H | -7.05753 | 0.699343 | -2.77338 |
| C | -1.52511 | 1.850937 | 4.307792 | H | -5.63733 | 0.976499 | -1.73154 |
| C | -2.41268 | 0.805494 | 4.613953 | H | -5.41886 | 0.723828 | -3.46371 |
| C | -2.09192 | -0.48771 | 4.186629 | H | -2.88237 | -3.54177 | -0.56759 |
| C | -0.91733 | -0.77609 | 3.486916 | H | -3.3022 | -4.81721 | -1.72484 |
| C | -0.70095 | -2.18787 | 3.006088 | H | -2.43788 | -3.35967 | -2.27279 |
| C | 0.584601 | 2.768458 | 3.309116 | H | -7.99586 | -5.18171 | -2.05767 |
| C | -3.68232 | 1.088976 | 5.363776 | H | -8.99739 | -3.76208 | -1.66413 |
| C | -4.64366 | -1.66647 | -2.06838 | H | -8.60955 | -4.1281 | -3.35306 |
| C | -4.54987 | -3.05967 | -1.87625 | H | 1.285344 | 0.895718 | 1.592075 |
| C | -5.71159 | -3.84056 | -1.9578 |  |  |  |  |

| **TS2** | | | | | | | |
| --- | --- | --- | --- | --- | --- | --- | --- |
| C | 2.888148 | 3.974755 | 0.071577 | C | -6.72944 | 1.783427 | -2.72352 |
| C | 4.243504 | 4.172208 | -0.32993 | C | -5.52692 | 2.126526 | -2.10181 |
| C | 2.273538 | 2.678312 | -0.13023 | C | -5.48062 | 3.257797 | -1.10525 |
| C | 2.904362 | 1.712579 | -0.95883 | C | -3.18033 | -0.43956 | -3.72961 |
| C | 4.26892 | 1.943943 | -1.30867 | C | -8.11904 | 0.398044 | -4.31587 |
| C | 4.922093 | 3.099333 | -0.97147 | H | 4.811556 | 1.193022 | -1.86305 |
| C | 1.004912 | 2.328901 | 0.47238 | H | 5.9685 | 3.23149 | -1.25626 |
| C | 0.119387 | 1.539162 | -0.30033 | H | -1.22428 | -0.09296 | -1.98747 |
| C | 0.67561 | 0.641118 | -1.23203 | H | -0.31609 | -2.27034 | -2.7408 |
| C | 2.129607 | 0.553829 | -1.40772 | H | 2.124753 | -2.64676 | -2.60905 |
| C | -0.17264 | -0.32171 | -1.88113 | H | 4.704812 | 0.116816 | -3.56315 |
| C | 0.32653 | -1.50874 | -2.29636 | H | 6.974744 | -0.8691 | -3.70428 |
| C | 1.716423 | -1.78137 | -2.08511 | H | 7.584229 | -2.82617 | -2.3005 |
| C | 2.635993 | -0.67002 | -1.89177 | H | 6.874239 | -4.63562 | -0.74364 |
| C | 4.044375 | -1.16541 | -1.97291 | H | 5.081625 | -5.78259 | 0.510374 |
| C | 4.972448 | -0.70012 | -2.89123 | H | 2.615932 | -6.06584 | 0.739967 |
| C | 6.258512 | -1.27962 | -2.98977 | H | 2.4969 | -4.76822 | 1.928993 |
| C | 6.596897 | -2.36716 | -2.21346 | H | 0.979993 | -4.94166 | -0.74136 |
| C | 5.642104 | -2.94789 | -1.33676 | H | 0.285392 | -5.06233 | 0.889064 |
| C | 4.358998 | -2.33984 | -1.20252 | H | 2.120517 | -1.33704 | 0.207283 |
| C | 5.887357 | -4.17574 | -0.654 | H | 5.905522 | 5.558334 | -0.43519 |
| C | 4.887356 | -4.81405 | 0.043292 | H | 4.627627 | 7.466857 | 0.520628 |
| C | 3.588519 | -4.24698 | 0.149652 | H | 2.207676 | 7.177761 | 1.081228 |
| C | 3.362799 | -2.99252 | -0.40508 | H | 1.114291 | 4.99953 | 0.807458 |
| C | 2.46383 | -4.98079 | 0.844974 | H | 2.595989 | 3.422736 | 2.412137 |
| C | 1.077632 | -4.5836 | 0.299442 | C | -6.80926 | 0.736336 | -3.65183 |
| C | 0.947953 | -3.08685 | 0.349752 | H | 2.148627 | 3.559885 | 4.815994 |
| C | 2.056948 | -2.33405 | -0.2289 | H | 0.010715 | 2.691192 | 5.752046 |
| C | -0.00718 | -2.37198 | 0.999813 | H | -2.13342 | 1.514275 | 5.422444 |
| C | 4.861662 | 5.434841 | -0.13756 | H | -3.72225 | 0.354406 | 3.911212 |
| C | 4.149635 | 6.494551 | 0.384609 | H | -3.88219 | -0.8555 | 1.723187 |
| C | 2.784873 | 6.326987 | 0.712879 | H | -4.546 | 0.670527 | 1.123341 |
| C | 2.170496 | 5.099891 | 0.56041 | H | -2.22832 | -0.93245 | -0.06493 |
| C | 0.67036 | 2.604286 | 1.871836 | H | -3.80623 | -0.61377 | -0.81675 |
| C | 1.62441 | 3.098668 | 2.774784 | H | -1.50139 | 2.816996 | -0.06572 |
| C | 1.381503 | 3.153481 | 4.15426 | H | -2.70174 | 2.781355 | -1.95347 |
| C | 0.19546 | 2.666323 | 4.675402 | H | -2.75642 | -2.38473 | 4.701765 |
| C | -0.7807 | 2.094631 | 3.825438 | H | -4.18516 | -4.454 | 1.214445 |
| C | -0.56341 | 2.085754 | 2.411105 | H | -3.08462 | -4.07452 | -0.89065 |
| C | -1.96148 | 1.494069 | 4.34395 | H | -1.46033 | -3.35017 | -0.98989 |
| C | -2.84415 | 0.861046 | 3.504764 | H | -1.6559 | -5.03649 | -0.48427 |
| C | -2.64067 | 0.840828 | 2.09784 | H | -0.17535 | -0.62167 | 3.193379 |
| C | -1.54139 | 1.503262 | 1.561584 | H | -0.66674 | -1.33244 | 4.741409 |
| C | -3.61253 | 0.08922 | 1.225119 | H | 0.587969 | -2.11859 | 3.748862 |
| C | -3.03361 | -0.18836 | -0.16471 | H | -5.21562 | -3.02263 | 4.433381 |
| C | -2.49221 | 1.097366 | -0.72828 | H | -5.68823 | -4.10445 | 3.095317 |
| C | -1.36078 | 1.728076 | 0.066256 | H | -4.69949 | -4.71752 | 4.436921 |
| C | -3.10151 | 1.787625 | -1.70858 | H | -5.67394 | -0.7755 | -4.68186 |
| C | -1.22474 | -2.82475 | 1.67807 | H | -7.63229 | 2.349781 | -2.47717 |
| C | -1.43107 | -2.37841 | 3.011697 | H | -6.47573 | 3.699766 | -0.95658 |
| C | -2.61101 | -2.71914 | 3.67105 | H | -5.10496 | 2.919202 | -0.12608 |
| C | -3.61419 | -3.47531 | 3.045979 | H | -4.80261 | 4.062151 | -1.43714 |
| C | -3.40206 | -3.88763 | 1.725946 | H | -2.88302 | -1.14494 | -2.9345 |
| C | -2.2258 | -3.58544 | 1.027636 | H | -3.36074 | -1.03316 | -4.63668 |
| C | -2.09739 | -4.02908 | -0.40844 | H | -2.32059 | 0.219465 | -3.92089 |
| C | -0.36891 | -1.57381 | 3.712914 | H | -8.03219 | -0.49283 | -4.95426 |
| C | -4.87288 | -3.8438 | 3.785387 | H | -8.90717 | 0.207379 | -3.56955 |
| C | -4.35338 | 1.394018 | -2.40526 | H | -8.47005 | 1.231536 | -4.9468 |
| C | -4.41023 | 0.345883 | -3.34626 | H | 0.174433 | -1.292 | 1.066326 |
| C | -5.63749 | 0.035217 | -3.94882 |  |  |  |  |

| **INT5** | | | | | | | |
| --- | --- | --- | --- | --- | --- | --- | --- |
| C | 3.020971 | 3.888369 | 0.507652 | C | 3.974012 | -0.96367 | -2.12089 |
| C | 4.307742 | 4.211126 | -0.02562 | C | 4.952936 | -0.42935 | -2.94752 |
| C | 2.384348 | 2.653838 | 0.098694 | C | 6.228451 | -1.02663 | -3.05953 |
| C | 2.919869 | 1.888069 | -0.96959 | C | 6.520414 | -2.18121 | -2.36629 |
| C | 4.19623 | 2.254603 | -1.47327 | C | 5.524147 | -2.82575 | -1.5844 |
| C | 4.88061 | 3.343149 | -0.99167 | C | -2.56499 | 0.18718 | 3.595808 |
| C | 1.163732 | 2.172155 | 0.712353 | C | -2.44786 | 0.470988 | 2.208021 |
| C | 0.196353 | 1.577596 | -0.15712 | C | -1.35068 | 1.197902 | 1.751902 |
| C | 0.675285 | 0.856739 | -1.24607 | C | -3.50107 | -0.03883 | 1.25965 |
| C | 2.12539 | 0.767952 | -1.49504 | C | -3.01036 | -0.04237 | -0.19127 |
| C | -0.21645 | 0.010115 | -2.01729 | C | -2.43501 | 1.30816 | -0.51927 |
| C | 0.223984 | -1.18775 | -2.42472 | C | -1.24768 | 1.731931 | 0.328971 |
| C | 1.61679 | -1.58382 | -2.13863 | C | -3.04608 | 2.198408 | -1.32096 |
| C | 2.593546 | -0.44151 | -2.00729 | C | -1.44966 | -3.10636 | 1.158653 |
| C | 3.974012 | -0.96367 | -2.12089 | C | -1.5498 | -3.00275 | 2.565132 |
| C | 4.952936 | -0.42935 | -2.94752 | C | -2.71991 | -3.42654 | 3.202263 |
| C | 6.228451 | -1.02663 | -3.05953 | C | -3.80016 | -3.953 | 2.482086 |
| C | 6.520414 | -2.18121 | -2.36629 | C | -3.6776 | -4.05338 | 1.090059 |
| C | 5.524147 | -2.82575 | -1.5844 | C | -2.52213 | -3.6438 | 0.413059 |
| C | 4.232587 | -2.22842 | -1.46763 | C | -2.45886 | -3.743 | -1.09113 |
| C | 5.748189 | -4.08999 | -0.97025 | C | -0.38972 | -2.46391 | 3.360845 |
| C | 4.721326 | -4.76139 | -0.34883 | C | -5.04437 | -4.43054 | 3.185258 |
| C | 3.416061 | -4.20232 | -0.26669 | C | -4.34325 | 2.000155 | -2.01796 |
| C | 3.199728 | -2.92997 | -0.77313 | C | -4.49249 | 1.160673 | -3.14057 |
| C | 2.268482 | -5.00018 | 0.313444 | C | -5.75837 | 1.024756 | -3.72761 |
| C | 0.918482 | -4.61369 | -0.32294 | C | -6.87993 | 1.700807 | -3.23987 |
| C | 0.729115 | -3.13297 | -0.17524 | C | -6.70838 | 2.540638 | -2.13097 |
| C | 1.847568 | -2.26911 | -0.69831 | C | -5.46482 | 2.705535 | -1.51733 |
| C | -0.25572 | -2.52657 | 0.509229 | C | -5.32092 | 3.614201 | -0.32198 |
| C | 4.955086 | 5.414394 | 0.362204 | C | -3.32124 | 0.417106 | -3.7329 |
| C | 4.334197 | 6.30561 | 1.210375 | C | -8.23181 | 1.553657 | -3.88906 |
| C | 3.029286 | 6.029296 | 1.680374 | H | 4.638856 | 1.680502 | -2.27292 |
| C | 2.389567 | 4.855129 | 1.33744 | H | 5.862531 | 3.5885 | -1.40296 |
| C | 0.927829 | 2.126504 | 2.142938 | H | -1.23989 | 0.330543 | -2.1679 |
| C | 1.963851 | 2.377358 | 3.063194 | H | -0.43488 | -1.89798 | -2.92711 |
| C | 1.810145 | 2.126107 | 4.429563 | H | 1.967595 | -2.30397 | -2.88992 |
| C | 0.631043 | 1.572918 | 4.909217 | H | 4.720646 | 0.429142 | -3.57711 |
| C | -0.42489 | 1.240209 | 4.02996 | H | 6.975181 | -0.57137 | -3.71302 |
| C | -0.29656 | 1.538615 | 2.636473 | H | 7.507518 | -2.64219 | -2.44756 |
| C | -1.60125 | 0.584982 | 4.488964 | H | 6.740465 | -4.54107 | -1.04206 |
| C | -2.56499 | 0.18718 | 3.595808 | H | 4.896499 | -5.75479 | 0.071736 |
| C | -2.44786 | 0.470988 | 2.208021 | H | 2.463087 | -6.07464 | 0.173553 |
| C | -1.35068 | 1.197902 | 1.751902 | H | 2.207582 | -4.8354 | 1.404208 |
| C | -3.50107 | -0.03883 | 1.25965 | H | 0.934197 | -4.89799 | -1.39108 |
| C | -3.01036 | -0.04237 | -0.19127 | H | 0.09551 | -5.16092 | 0.157095 |
| C | -2.43501 | 1.30816 | -0.51927 | H | 1.920953 | -1.42532 | 0.003034 |
| C | -1.24768 | 1.731931 | 0.328971 | H | 5.94507 | 5.630835 | -0.04572 |
| C | -3.04608 | 2.198408 | -1.32096 | H | 4.832562 | 7.233397 | 1.49839 |
| C | -1.44966 | -3.10636 | 1.158653 | H | 2.516609 | 6.757055 | 2.313021 |
| C | 3.020971 | 3.888369 | 0.507652 | H | 1.378036 | 4.678921 | 1.700263 |
| C | 4.307742 | 4.211126 | -0.02562 | H | 2.923106 | 2.744599 | 2.706539 |
| C | 2.384348 | 2.653838 | 0.098694 | H | 2.634 | 2.342177 | 5.111966 |
| C | 2.919869 | 1.888069 | -0.96959 | H | 0.516227 | 1.360891 | 5.975106 |
| C | 4.19623 | 2.254603 | -1.47327 | H | -1.70428 | 0.369133 | 5.554566 |
| C | 4.88061 | 3.343149 | -0.99167 | H | -3.43811 | -0.37043 | 3.940621 |
| C | 1.163732 | 2.172155 | 0.712353 | H | -3.79608 | -1.05427 | 1.568129 |
| C | 0.196353 | 1.577596 | -0.15712 | H | -4.40485 | 0.591171 | 1.339797 |
| C | 0.675285 | 0.856739 | -1.24607 | H | -2.23981 | -0.82054 | -0.29708 |
| C | 2.12539 | 0.767952 | -1.49504 | H | -3.83715 | -0.29425 | -0.86637 |
| C | -0.21645 | 0.010115 | -2.01729 | H | -1.35376 | 2.827922 | 0.43795 |
| C | 0.223984 | -1.18775 | -2.42472 | H | -2.60938 | 3.204629 | -1.38726 |
| C | 4.232587 | -2.22842 | -1.46763 | H | -2.78933 | -3.34786 | 4.290951 |
| C | 5.748189 | -4.08999 | -0.97025 | H | -4.5127 | -4.4568 | 0.510626 |
| C | 4.721326 | -4.76139 | -0.34883 | H | -3.4166 | -4.08897 | -1.50396 |
| C | 3.416061 | -4.20232 | -0.26669 | H | -2.22417 | -2.76818 | -1.54591 |
| C | 3.199728 | -2.92997 | -0.77313 | H | -1.67825 | -4.44463 | -1.42399 |
| C | 2.268482 | -5.00018 | 0.313444 | H | -0.10907 | -1.45033 | 3.034452 |
| C | 0.918482 | -4.61369 | -0.32294 | H | -0.62418 | -2.419 | 4.432985 |
| C | 0.729115 | -3.13297 | -0.17524 | H | 0.504829 | -3.09394 | 3.228713 |
| C | 1.847568 | -2.26911 | -0.69831 | H | -5.19992 | -3.90244 | 4.137783 |
| C | -0.25572 | -2.52657 | 0.509229 | H | -5.94032 | -4.29087 | 2.562009 |
| C | 4.955086 | 5.414394 | 0.362204 | H | -4.97418 | -5.50736 | 3.416857 |
| C | 4.334197 | 6.30561 | 1.210375 | H | -5.86658 | 0.375622 | -4.60096 |
| C | 3.029286 | 6.029296 | 1.680374 | H | -7.5706 | 3.083113 | -1.73241 |
| C | 2.389567 | 4.855129 | 1.33744 | H | -6.28495 | 4.061803 | -0.0428 |
| C | 0.927829 | 2.126504 | 2.142938 | H | -4.928 | 3.071799 | 0.553348 |
| C | 1.963851 | 2.377358 | 3.063194 | H | -4.61419 | 4.437443 | -0.5212 |
| C | 1.810145 | 2.126107 | 4.429563 | H | -3.01767 | -0.43566 | -3.10172 |
| C | 0.631043 | 1.572918 | 4.909217 | H | -3.57312 | 0.011442 | -4.72255 |
| C | -0.42489 | 1.240209 | 4.02996 | H | -2.44298 | 1.069658 | -3.84867 |
| C | -0.29656 | 1.538615 | 2.636473 | H | -8.22051 | 0.792025 | -4.68181 |
| C | -1.60125 | 0.584982 | 4.488964 | H | -8.9994 | 1.267816 | -3.1519 |
| C | 1.61679 | -1.58382 | -2.13863 | H | -8.55946 | 2.504836 | -4.34074 |
| C | 2.593546 | -0.44151 | -2.00729 | H | -0.14866 | -1.44273 | 0.653316 |

| **7** | | | | | | | |
| --- | --- | --- | --- | --- | --- | --- | --- |
| C | 4.543872 | -2.76739 | 0.188355 | C | 0.506829 | 5.836026 | -3.80568 |
| C | 4.407737 | -4.18581 | 0.27774 | C | 1.232132 | 4.837672 | -3.15101 |
| C | 3.377534 | -1.91647 | 0.398425 | C | 2.737107 | 4.805701 | -3.24614 |
| C | 2.106367 | -2.51659 | 0.219014 | C | -1.63581 | 2.867981 | -1.53877 |
| C | 2.005372 | -3.93949 | 0.409466 | C | -1.64874 | 6.985169 | -4.45509 |
| C | 3.106521 | -4.7364 | 0.504214 | H | 1.013549 | -4.3782 | 0.500109 |
| C | 3.462204 | -0.49822 | 0.688935 | H | 3.003555 | -5.80925 | 0.682665 |
| C | 2.367235 | 0.306578 | 0.375721 | H | -0.04352 | 1.534451 | 0.570917 |
| C | 1.072687 | -0.28394 | 0.180361 | H | -2.22034 | 0.443035 | 0.666691 |
| C | 0.939103 | -1.69478 | -0.0079 | H | 1.495832 | -3.48858 | -1.92977 |
| C | -0.11061 | 0.475782 | 0.355478 | H | 1.170561 | -5.29374 | -3.54723 |
| C | -1.34384 | -0.13646 | 0.389298 | H | -1.11912 | -6.17533 | -4.01019 |
| C | -1.49426 | -1.4808 | 0.003763 | H | -3.53804 | -5.98464 | -3.72326 |
| C | -0.35939 | -2.21707 | -0.37795 | H | -5.49744 | -4.7947 | -2.7743 |
| C | -0.55628 | -3.36758 | -1.29498 | H | -6.2851 | -2.71995 | -1.80012 |
| C | 0.49307 | -3.88729 | -2.05361 | H | -5.96511 | -3.18995 | -0.12645 |
| C | 0.304194 | -4.90563 | -3.00672 | H | -4.71184 | -0.78658 | -1.56796 |
| C | -0.95851 | -5.38909 | -3.26851 | H | -5.97563 | -0.71285 | -0.31372 |
| C | -2.08144 | -4.82039 | -2.61538 | H | -2.70578 | -2.84691 | 0.999327 |
| C | -1.89104 | -3.8072 | -1.61655 | H | 5.422406 | -6.09466 | 0.19054 |
| C | -3.40242 | -5.19695 | -2.978 | H | 7.629927 | -5.12166 | -0.42 |
| C | -4.48546 | -4.54009 | -2.44781 | H | 7.833359 | -2.65035 | -0.77343 |
| C | -4.31558 | -3.50679 | -1.49085 | H | 5.906264 | -1.17675 | -0.37761 |
| C | -3.03998 | -3.18726 | -1.04059 | H | 5.421578 | -1.75983 | 2.140774 |
| C | -5.51043 | -2.71825 | -1.0169 | H | 7.049687 | -0.80202 | 3.680643 |
| C | -5.10475 | -1.28187 | -0.66163 | H | 7.088042 | 1.655801 | 4.119221 |
| C | -4.05071 | -1.341 | 0.404069 | H | 6.068629 | 3.862237 | 3.810917 |
| C | -2.83495 | -2.20972 | 0.10054 | H | 4.292798 | 5.289938 | 2.833611 |
| C | -4.19749 | -0.78528 | 1.620664 | H | 2.029351 | 5.304612 | 1.871928 |
| C | 5.542123 | -5.01298 | 0.088014 | H | 2.717423 | 5.207211 | 0.249088 |
| C | 6.765659 | -4.47267 | -0.26037 | H | 0.68262 | 3.273096 | 1.471959 |
| C | 6.883236 | -3.07895 | -0.44624 | H | 0.384042 | 4.427314 | 0.14754 |
| C | 5.79898 | -2.24979 | -0.22394 | H | 3.393488 | 1.699333 | -0.72519 |
| C | 4.545551 | 0.10657 | 1.518386 | H | 1.878012 | 2.109642 | -2.36342 |
| C | 5.439344 | -0.68017 | 2.246913 | H | -7.61037 | 0.077855 | 4.630318 |
| C | 6.361282 | -0.13258 | 3.159658 | H | -6.98117 | 2.991627 | 1.53653 |
| C | 6.380657 | 1.22162 | 3.408505 | H | -4.89831 | 2.935959 | 0.309476 |
| C | 5.437176 | 2.065849 | 2.771443 | H | -3.91038 | 1.459011 | 0.199439 |
| C | 4.519211 | 1.517252 | 1.816792 | H | -5.44533 | 1.56093 | -0.66374 |
| C | 5.355403 | 3.448195 | 3.093857 | H | -4.62673 | -1.61972 | 4.338318 |
| C | 4.37332 | 4.237275 | 2.548879 | H | -6.33513 | -1.87067 | 4.765008 |
| C | 3.437976 | 3.706947 | 1.620745 | H | -5.68146 | -2.4967 | 3.226948 |
| C | 3.540793 | 2.376569 | 1.235488 | H | -8.39185 | 2.724716 | 4.746754 |
| C | 2.339261 | 4.594803 | 1.087705 | H | -8.72017 | 3.379324 | 3.123639 |
| C | 1.140777 | 3.77347 | 0.600128 | H | -9.52163 | 1.886794 | 3.669924 |
| C | 1.637169 | 2.757236 | -0.39065 | H | -2.64384 | 4.943029 | -2.92653 |
| C | 2.692149 | 1.787801 | 0.12622 | H | 1.048158 | 6.588195 | -4.38768 |
| C | 1.364831 | 2.823991 | -1.70503 | H | 3.117118 | 5.641446 | -3.85112 |
| C | -5.31012 | 0.067645 | 2.096824 | H | 3.204127 | 4.858973 | -2.24964 |
| C | -6.01845 | -0.35501 | 3.252556 | H | 3.094052 | 3.86746 | -3.70299 |
| C | -7.06552 | 0.422563 | 3.746154 | H | -1.54866 | 3.024603 | -0.45068 |
| C | -7.43767 | 1.630715 | 3.138221 | H | -2.70578 | 2.907108 | -1.78883 |
| C | -6.7197 | 2.043408 | 2.015724 | H | -1.26947 | 1.851501 | -1.73743 |
| C | -5.66149 | 1.290019 | 1.481249 | H | -2.73053 | 6.915267 | -4.26952 |
| C | -4.93666 | 1.83685 | 0.275208 | H | -1.31426 | 7.985999 | -4.13524 |
| C | -5.65217 | -1.65049 | 3.932044 | H | -1.48934 | 6.92736 | -5.54494 |
| C | -8.57399 | 2.450616 | 3.694316 | H | -3.42935 | -1.0158 | 2.370181 |
| C | 0.546422 | 3.862684 | -2.38564 | H | -2.73053 | 6.915267 | -4.26952 |
| C | -0.86106 | 3.902219 | -2.3136 | H | -1.31426 | 7.985999 | -4.13524 |
| C | -1.55172 | 4.921482 | -2.98608 | H | -1.48934 | 6.92736 | -5.54494 |
| C | -0.89123 | 5.898917 | -3.73404 |  |  |  |  |

| **INT6** | | | | | | | |
| --- | --- | --- | --- | --- | --- | --- | --- |
| C | -4.42637 | -2.69025 | -0.47773 | C | 1.29772 | 4.532266 | 3.160569 |
| C | -4.21563 | -4.10026 | -0.52418 | C | 0.746855 | 4.651946 | 4.441334 |
| C | -3.27362 | -1.79835 | -0.60598 | C | -0.44877 | 3.970977 | 4.709797 |
| C | -1.97735 | -2.32889 | -0.32408 | C | -1.09329 | 3.20702 | 3.736717 |
| C | -1.8087 | -3.74609 | -0.44465 | C | -2.38064 | 2.492462 | 4.061943 |
| C | -2.87381 | -4.58704 | -0.6069 | C | 1.399644 | 3.61605 | 0.828837 |
| C | -3.38339 | -0.42795 | -1.00441 | C | 1.410436 | 5.485363 | 5.505048 |
| C | -2.31043 | 0.463733 | -0.69793 | H | -0.80079 | -4.1543 | -0.42484 |
| C | -0.97942 | -0.0905 | -0.46713 | H | -2.70767 | -5.65955 | -0.73198 |
| C | -0.83582 | -1.45743 | -0.10851 | H | 0.085976 | 1.67335 | -1.13493 |
| C | 0.177135 | 0.654022 | -0.76629 | H | 2.295497 | 0.602223 | -1.05429 |
| C | 1.42288 | 0.060608 | -0.69714 | H | -1.41828 | -3.07102 | 1.942254 |
| C | 1.59188 | -1.21342 | -0.12961 | H | -1.10259 | -4.7323 | 3.713317 |
| C | 0.462062 | -1.91861 | 0.330293 | H | 1.189291 | -5.51239 | 4.30758 |
| C | 0.651328 | -2.97431 | 1.346572 | H | 3.614756 | -5.29474 | 4.072766 |
| C | -0.41265 | -3.44478 | 2.119026 | H | 5.572749 | -4.15094 | 3.069132 |
| C | -0.23284 | -4.37427 | 3.158576 | H | 6.342632 | -2.15476 | 1.924954 |
| C | 1.0351 | -4.80072 | 3.493168 | H | 6.087056 | -2.80441 | 0.302114 |
| C | 2.166283 | -4.26908 | 2.824237 | H | 4.745819 | -0.28913 | 1.445082 |
| C | 1.985887 | -3.35613 | 1.732151 | H | 6.041944 | -0.32335 | 0.224359 |
| C | 3.482986 | -4.58178 | 3.255705 | H | 2.848184 | -2.65013 | -0.95161 |
| C | 4.56579 | -3.95103 | 2.694255 | H | -5.13521 | -6.05961 | -0.49052 |
| C | 4.40321 | -3.01713 | 1.638344 | H | -7.4365 | -5.18621 | -0.11569 |
| C | 3.134861 | -2.77224 | 1.123861 | H | -7.78835 | -2.72743 | 0.172557 |
| C | 5.594753 | -2.25167 | 1.122637 | H | -5.91385 | -1.16205 | -0.06152 |
| C | 5.171636 | -0.86982 | 0.606838 | H | -5.03221 | -1.77153 | -2.73533 |
| C | 4.153758 | -1.06368 | -0.47807 | H | -6.84904 | -0.83602 | -4.10769 |
| C | 2.946338 | -1.92282 | -0.11944 | H | -7.38969 | 1.596921 | -3.98631 |
| C | 4.326922 | -0.63827 | -1.74331 | H | -6.8575 | 3.855945 | -3.11576 |
| C | -5.31706 | -4.98438 | -0.42534 | H | -5.42134 | 5.344131 | -1.75482 |
| C | -6.59296 | -4.49904 | -0.2072 | H | -3.25 | 5.70946 | -0.73942 |
| C | -6.79279 | -3.11113 | -0.06067 | H | -3.66588 | 4.785108 | 0.70644 |
| C | -5.73494 | -2.22805 | -0.19607 | H | -1.27785 | 4.289648 | -1.1527 |
| C | -4.47524 | 0.10336 | -1.81771 | H | -1.23643 | 4.810127 | 0.546468 |
| C | -5.24392 | -0.70605 | -2.67159 | H | -1.47815 | 1.291118 | 1.728713 |
| C | -6.27271 | -0.17363 | -3.45949 | H | 7.77996 | 0.038772 | -4.75106 |
| C | -6.56273 | 1.180889 | -3.40636 | H | 7.006027 | 3.207376 | -1.9563 |
| C | -5.76673 | 2.046582 | -2.6187 | H | 4.884239 | 3.208662 | -0.78616 |
| C | -4.68057 | 1.510131 | -1.86519 | H | 3.958179 | 1.710623 | -0.51925 |
| C | -6.01586 | 3.443023 | -2.55451 | H | 5.480126 | 1.974532 | 0.334032 |
| C | -5.21189 | 4.273178 | -1.79733 | H | 4.836822 | -1.71065 | -4.36369 |
| C | -4.09376 | 3.769416 | -1.10153 | H | 6.559083 | -1.94634 | -4.7339 |
| C | -3.80472 | 2.386338 | -1.15617 | H | 5.895829 | -2.4515 | -3.15818 |
| C | -3.22328 | 4.699132 | -0.30461 | H | 8.526874 | 2.638944 | -5.10266 |
| C | -1.78537 | 4.19456 | -0.17591 | H | 8.746869 | 3.508136 | -3.56324 |
| C | -1.7889 | 2.750341 | 0.258011 | H | 9.628695 | 1.994315 | -3.87515 |
| C | -2.60366 | 1.845744 | -0.56279 | H | 2.249207 | 5.02525 | 2.944472 |
| C | -1.26251 | 2.324218 | 1.437246 | H | -0.89083 | 4.03905 | 5.707696 |
| C | 5.425563 | 0.198132 | -2.2745 | H | -2.68352 | 2.67317 | 5.102406 |
| C | 6.16916 | -0.31016 | -3.37255 | H | -3.20404 | 2.817959 | 3.405602 |
| C | 7.206088 | 0.447823 | -3.91438 | H | -2.28258 | 1.401517 | 3.927805 |
| C | 7.533481 | 1.71705 | -3.41387 | H | 0.809252 | 3.969512 | -0.02911 |
| C | 6.779314 | 2.21209 | -2.34962 | H | 2.346588 | 4.172955 | 0.826413 |
| C | 5.730354 | 1.481815 | -1.7679 | H | 1.634766 | 2.557665 | 0.644598 |
| C | 4.968566 | 2.122331 | -0.63172 | H | 2.428279 | 5.781158 | 5.213863 |
| C | 5.853817 | -1.67277 | -3.93667 | H | 0.835133 | 6.407337 | 5.695505 |
| C | 8.664151 | 2.509912 | -4.01669 | H | 1.469164 | 4.941059 | 6.460664 |
| C | -0.53963 | 3.130889 | 2.431693 | H | 3.594414 | -0.97777 | -2.48776 |
| C | 0.685478 | 3.783665 | 2.148417 |  |  |  |  |

| **TS3** | | | | | | | |
| --- | --- | --- | --- | --- | --- | --- | --- |
| C | 4.982609 | 2.139382 | -0.97155 | C | -2.93766 | -3.50839 | 1.681778 |
| C | 4.873687 | 3.477783 | -1.46437 | C | -3.51731 | -2.7438 | 2.696378 |
| C | 3.786733 | 1.301563 | -0.90936 | C | -2.77916 | -1.66541 | 3.208157 |
| C | 2.531392 | 1.976085 | -0.91883 | C | -1.50672 | -1.34949 | 2.738045 |
| C | 2.457324 | 3.314469 | -1.42537 | C | -0.80831 | -0.13686 | 3.308254 |
| C | 3.581061 | 4.009359 | -1.76462 | C | -1.19483 | -4.08493 | 0.01719 |
| C | 3.818046 | -0.13843 | -0.7418 | C | -4.91086 | -3.02106 | 3.188319 |
| C | 2.641869 | -0.78887 | -0.34378 | H | 1.473412 | 3.760922 | -1.571 |
| C | 1.380378 | -0.12113 | -0.49574 | H | 3.506119 | 5.006903 | -2.20298 |
| C | 1.321868 | 1.274881 | -0.5562 | H | 0.242078 | -1.86729 | -1.09444 |
| C | 0.162148 | -0.91048 | -0.581 | H | -1.97374 | -0.78218 | -0.91489 |
| C | -1.06634 | -0.22776 | -0.68641 | H | 2.052622 | 3.487856 | 0.839948 |
| C | -1.14231 | 1.122032 | -0.39894 | H | 1.877039 | 5.186767 | 2.576161 |
| C | 0.048565 | 1.870288 | -0.15042 | H | -0.31512 | 5.660917 | 3.662016 |
| C | -0.06074 | 3.042208 | 0.721674 | H | -2.68912 | 5.127681 | 3.953692 |
| C | 1.063712 | 3.72172 | 1.219096 | H | -4.67125 | 3.764742 | 3.348156 |
| C | 0.971876 | 4.677186 | 2.240596 | H | -5.42009 | 1.655624 | 2.300743 |
| C | -0.24385 | 4.93939 | 2.844613 | H | -5.60796 | 2.457555 | 0.739921 |
| C | -1.41003 | 4.248309 | 2.436466 | H | -3.83681 | 0.010671 | 1.297867 |
| C | -1.34281 | 3.331506 | 1.338181 | H | -5.35026 | 0.024231 | 0.359521 |
| C | -2.64305 | 4.418956 | 3.123871 | H | -2.41827 | 2.608883 | -1.16371 |
| C | -3.74257 | 3.669872 | 2.779976 | H | 5.928458 | 5.294443 | -1.98478 |
| C | -3.69684 | 2.76606 | 1.686354 | H | 8.140367 | 4.473758 | -1.19371 |
| C | -2.52848 | 2.66033 | 0.938908 | H | 8.296976 | 2.227326 | -0.09968 |
| C | -4.88936 | 1.899396 | 1.366583 | H | 6.337112 | 0.770553 | 0.035724 |
| C | -4.46883 | 0.62054 | 0.628042 | H | 5.852834 | 0.277596 | -2.55892 |
| C | -3.69704 | 1.011017 | -0.59948 | H | 7.729647 | -1.22671 | -3.08557 |
| C | -2.46712 | 1.862524 | -0.34704 | H | 8.002415 | -3.37975 | -1.86102 |
| C | -4.09593 | 0.735223 | -1.85229 | H | 7.123265 | -5.0167 | -0.20458 |
| C | 6.031448 | 4.288115 | -1.57204 | H | 5.370046 | -5.71306 | 1.402477 |
| C | 7.256327 | 3.836803 | -1.12152 | H | 3.049112 | -5.48848 | 2.197379 |
| C | 7.347515 | 2.563423 | -0.52197 | H | 3.192043 | -3.87923 | 2.919175 |
| C | 6.240461 | 1.73752 | -0.45222 | H | 1.376833 | -4.71717 | 0.59815 |
| C | 4.950823 | -1.02024 | -1.09634 | H | 0.849663 | -4.22831 | 2.214138 |
| C | 5.915971 | -0.67876 | -2.04361 | H | 0.71023 | -0.8014 | 1.66067 |
| C | 6.992655 | -1.53751 | -2.34245 | H | -8.38364 | 0.511277 | -3.61144 |
| C | 7.137289 | -2.73856 | -1.67821 | H | -6.64212 | -3.17422 | -2.25113 |
| C | 6.142542 | -3.1642 | -0.75863 | H | -4.48201 | -3.29241 | -1.44594 |
| C | 4.999885 | -2.33421 | -0.53376 | H | -3.33535 | -2.08211 | -2.06065 |
| C | 6.242012 | -4.38988 | -0.04873 | H | -4.00583 | -1.92114 | -0.42822 |
| C | 5.256421 | -4.78526 | 0.837058 | H | -5.47255 | 2.41484 | -3.70651 |
| C | 4.08004 | -4.02534 | 0.99204 | H | -7.2308 | 2.548412 | -3.47212 |
| C | 3.925848 | -2.83691 | 0.262705 | H | -6.11837 | 2.688173 | -2.08613 |
| C | 3.000138 | -4.41396 | 1.968686 | H | -9.80664 | -1.71319 | -2.87932 |
| C | 1.613155 | -4.04848 | 1.44555 | H | -9.08898 | -2.08181 | -4.45585 |
| C | 1.56415 | -2.6117 | 0.972049 | H | -8.87391 | -3.21424 | -3.10019 |
| C | 2.672379 | -2.10136 | 0.296337 | H | -3.50879 | -4.33268 | 1.246664 |
| C | 0.406086 | -1.75441 | 1.227358 | H | -3.21999 | -1.04561 | 3.993129 |
| C | -5.30934 | -0.03365 | -2.22844 | H | -1.41748 | 0.319592 | 4.099635 |
| C | -6.39248 | 0.663927 | -2.8128 | H | 0.172745 | -0.38238 | 3.744546 |
| C | -7.54754 | -0.03714 | -3.16788 | H | -0.64616 | 0.638078 | 2.541457 |
| C | -7.66347 | -1.41947 | -2.96889 | H | -0.31851 | -3.68083 | -0.49886 |
| C | -6.57707 | -2.09304 | -2.40255 | H | -0.93733 | -5.10332 | 0.348711 |
| C | -5.40051 | -1.42745 | -2.03214 | H | -2.00395 | -4.18548 | -0.7216 |
| C | -4.24928 | -2.218 | -1.45946 | H | -5.25278 | -4.02529 | 2.901122 |
| C | -6.3048 | 2.153284 | -3.03156 | H | -4.9757 | -2.93047 | 4.283349 |
| C | -8.92023 | -2.14897 | -3.36831 | H | -5.62135 | -2.2922 | 2.760858 |
| C | -0.91241 | -2.16557 | 1.725403 | H | -3.51116 | 1.163206 | -2.67875 |
| C | -1.66031 | -3.23953 | 1.17682 |  |  |  |  |

| **INT7** | | | | | | | |
| --- | --- | --- | --- | --- | --- | --- | --- |
| C | 4.964312 | 2.088232 | -0.86903 | C | -2.45455 | -4.5632 | 0.293983 |
| C | 4.88435 | 3.416777 | -1.39027 | C | -3.32292 | -4.52668 | 1.385553 |
| C | 3.737038 | 1.312632 | -0.71502 | C | -3.0573 | -3.59444 | 2.395115 |
| C | 2.510989 | 2.029973 | -0.68176 | C | -1.95172 | -2.74061 | 2.343685 |
| C | 2.463714 | 3.348513 | -1.24029 | C | -1.74806 | -1.75393 | 3.471107 |
| C | 3.598879 | 3.987237 | -1.6474 | C | -0.5108 | -3.81208 | -1.06986 |
| C | 3.703397 | -0.12496 | -0.55903 | C | -4.51642 | -5.4408 | 1.476567 |
| C | 2.540459 | -0.73186 | -0.03304 | H | 1.494901 | 3.832039 | -1.36239 |
| C | 1.329449 | -0.01755 | -0.00238 | H | 3.541238 | 4.973905 | -2.11236 |
| C | 1.308833 | 1.378851 | -0.20484 | H | 0.02405 | -1.3789 | -0.81624 |
| C | 0.072736 | -0.81662 | 0.14156 | H | -2.11994 | -0.5465 | 0.094423 |
| C | -1.17011 | -0.01283 | 0.137237 | H | 2.216147 | 3.419327 | 1.257163 |
| C | -1.15357 | 1.338203 | 0.106794 | H | 2.167352 | 5.456471 | 2.618681 |
| C | 0.112323 | 2.053061 | 0.224372 | H | -0.00784 | 6.503685 | 3.227883 |
| C | 0.08162 | 3.324369 | 0.917239 | H | -2.45613 | 6.454734 | 3.247413 |
| C | 1.257894 | 3.897466 | 1.4409 | H | -4.57839 | 5.322656 | 2.640636 |
| C | 1.23114 | 5.041336 | 2.241581 | H | -5.61255 | 3.216562 | 1.964522 |
| C | 0.018154 | 5.618397 | 2.587693 | H | -5.407 | 3.528819 | 0.238919 |
| C | -1.20497 | 5.049243 | 2.163451 | H | -4.25602 | 1.128162 | 1.769124 |
| C | -1.1906 | 3.901218 | 1.305521 | H | -5.60743 | 1.081117 | 0.61169 |
| C | -2.45109 | 5.572093 | 2.604288 | H | -2.18028 | 2.707083 | -1.08681 |
| C | -3.62549 | 4.947228 | 2.26013 | H | 5.986672 | 5.174293 | -2.00933 |
| C | -3.62748 | 3.804902 | 1.417985 | H | 8.20036 | 4.269935 | -1.32241 |
| C | -2.42365 | 3.327099 | 0.905329 | H | 8.324292 | 2.036394 | -0.20136 |
| C | -4.91633 | 3.081889 | 1.122322 | H | 6.311855 | 0.667821 | 0.074801 |
| C | -4.66606 | 1.590689 | 0.852518 | H | 5.535325 | 0.269783 | -2.5775 |
| C | -3.6915 | 1.460252 | -0.28196 | H | 7.346524 | -1.25339 | -3.26748 |
| C | -2.38065 | 2.209274 | -0.11644 | H | 7.705521 | -3.40521 | -2.07173 |
| C | -3.96606 | 0.835543 | -1.44034 | H | 6.954281 | -5.06092 | -0.36136 |
| C | 6.067737 | 4.173312 | -1.57915 | H | 5.302756 | -5.76396 | 1.351258 |
| C | 7.294711 | 3.674942 | -1.1875 | H | 3.051985 | -5.50041 | 2.342334 |
| C | 7.366186 | 2.408027 | -0.57084 | H | 3.280308 | -3.90188 | 3.055546 |
| C | 6.230643 | 1.63352 | -0.41954 | H | 1.160011 | -4.75811 | 1.011828 |
| C | 4.754006 | -1.02991 | -1.04447 | H | 0.911801 | -4.08414 | 2.603726 |
| C | 5.636188 | -0.68875 | -2.07227 | H | 0.211874 | -1.39877 | 2.199699 |
| C | 6.674105 | -1.55474 | -2.462 | H | -7.8395 | 0.430071 | -3.95175 |
| C | 6.865529 | -2.758 | -1.80854 | H | -7.09301 | -2.62315 | -1.02135 |
| C | 5.955116 | -3.18692 | -0.80712 | H | -5.1849 | -2.66943 | 0.25101 |
| C | 4.839248 | -2.35006 | -0.48495 | H | -3.73599 | -1.96593 | -0.47378 |
| C | 6.096121 | -4.4263 | -0.12906 | H | -4.59733 | -1.09493 | 0.801618 |
| C | 5.167379 | -4.82234 | 0.81354 | H | -4.65713 | 1.811661 | -4.03892 |
| C | 4.017209 | -4.04444 | 1.071326 | H | -6.37116 | 2.227585 | -4.26288 |
| C | 3.83112 | -2.84369 | 0.384794 | H | -5.49528 | 2.757476 | -2.80406 |
| C | 3.002213 | -4.42452 | 2.120162 | H | -8.92531 | -2.08841 | -3.99072 |
| C | 1.578743 | -4.02744 | 1.729678 | H | -9.06371 | -2.78647 | -2.35816 |
| C | 1.485778 | -2.65786 | 1.108496 | H | -9.77764 | -1.19686 | -2.71987 |
| C | 2.567948 | -2.10409 | 0.490516 | H | -2.65019 | -5.26956 | -0.51732 |
| C | 0.167533 | -1.91408 | 1.226268 | H | -3.73267 | -3.53067 | 3.252734 |
| C | -5.21728 | 0.120078 | -1.78469 | H | -2.56937 | -1.82214 | 4.197633 |
| C | -6.0279 | 0.654901 | -2.81499 | H | -0.80972 | -1.93432 | 4.021307 |
| C | -7.21381 | 0.004442 | -3.16186 | H | -1.71436 | -0.71355 | 3.106825 |
| C | -7.62301 | -1.17416 | -2.52305 | H | 0.559153 | -3.62666 | -0.91008 |
| C | -6.79978 | -1.69565 | -1.52096 | H | -0.6161 | -4.80591 | -1.52768 |
| C | -5.60154 | -1.07516 | -1.14003 | H | -0.85775 | -3.08309 | -1.82356 |
| C | -4.73917 | -1.72614 | -0.08788 | H | -4.60826 | -6.07826 | 0.585848 |
| C | -5.62243 | 1.926535 | -3.51693 | H | -4.4467 | -6.0983 | 2.358489 |
| C | -8.91132 | -1.8506 | -2.91461 | H | -5.45116 | -4.86546 | 1.580994 |
| C | -1.06343 | -2.81322 | 1.242789 | H | -3.21777 | 0.91296 | -2.24191 |
| C | -1.33816 | -3.71949 | 0.193721 |  |  |  |  |

| **INT8** | | | | | | | |
| --- | --- | --- | --- | --- | --- | --- | --- |
| C | -5.14704 | 1.607633 | 0.498473 | C | 1.45872 | -2.82717 | 0.740894 |
| C | -5.24937 | 2.865335 | 1.166928 | C | 2.619201 | -3.37995 | 1.292621 |
| C | -3.84365 | 0.967872 | 0.369919 | C | 3.730717 | -3.71811 | 0.51159 |
| C | -2.70437 | 1.799969 | 0.482157 | C | 3.650664 | -3.48964 | -0.86244 |
| C | -2.83476 | 3.011908 | 1.243113 | C | 2.51097 | -2.92971 | -1.45799 |
| C | -4.05069 | 3.49372 | 1.62985 | C | 2.516594 | -2.69313 | -2.95112 |
| C | -3.66054 | -0.44956 | 0.126446 | C | 0.305695 | -2.49208 | 1.654844 |
| C | -2.43639 | -0.8936 | -0.37438 | C | 4.965524 | -4.30251 | 1.145407 |
| C | -1.30617 | 0.00047 | -0.45436 | H | -1.92921 | 3.543225 | 1.529024 |
| C | -1.4324 | 1.370514 | -0.06841 | H | -4.12207 | 4.399485 | 2.236513 |
| C | -0.0246 | -0.50893 | -0.78406 | H | 2.074415 | -0.16214 | -0.65811 |
| C | 1.089513 | 0.286065 | -0.56547 | H | -2.42648 | 3.678309 | -1.10722 |
| C | 0.980713 | 1.639814 | -0.23643 | H | -2.53672 | 6.051553 | -1.71 |
| C | -0.29275 | 2.240763 | -0.20758 | H | -0.49672 | 7.483482 | -1.57166 |
| C | -0.3628 | 3.691198 | -0.50275 | H | 1.917312 | 7.677173 | -1.23181 |
| C | -1.52898 | 4.28028 | -0.99083 | H | 4.113651 | 6.613847 | -0.78433 |
| C | -1.58828 | 5.637286 | -1.36038 | H | 5.384436 | 4.567797 | -0.76892 |
| C | -0.46202 | 6.427573 | -1.29256 | H | 4.945404 | 4.014531 | 0.851823 |
| C | 0.779818 | 5.858837 | -0.90945 | H | 4.312933 | 2.538533 | -1.76234 |
| C | 0.843702 | 4.478824 | -0.51945 | H | 5.559139 | 2.069703 | -0.58111 |
| C | 1.977189 | 6.621829 | -0.95437 | H | 1.997803 | 2.525509 | 1.334749 |
| C | 3.191861 | 6.03132 | -0.70416 | H | -6.57871 | 4.428292 | 1.855189 |
| C | 3.279007 | 4.656457 | -0.36718 | H | -8.61871 | 3.408584 | 0.859482 |
| C | 2.118903 | 3.901954 | -0.23937 | H | -8.41731 | 1.304117 | -0.4828 |
| C | 4.626382 | 4.000274 | -0.20671 | H | -6.24766 | 0.169659 | -0.69706 |
| C | 4.576009 | 2.544799 | -0.68912 | H | -5.65631 | -0.25033 | 2.035271 |
| C | 3.546571 | 1.801287 | 0.11045 | H | -7.35219 | -1.93045 | 2.615162 |
| C | 2.1773 | 2.464995 | 0.241264 | H | -7.3989 | -4.13756 | 1.458594 |
| C | 3.809887 | 0.671787 | 0.795785 | H | -6.31571 | -5.74727 | -0.10554 |
| C | -6.51746 | 3.480266 | 1.314441 | H | -4.41784 | -6.33531 | -1.59425 |
| C | -7.64826 | 2.918007 | 0.753325 | H | -2.1149 | -5.89046 | -2.30196 |
| C | -7.5368 | 1.724365 | 0.008676 | H | -2.34535 | -4.36838 | -3.17845 |
| C | -6.31688 | 1.084191 | -0.11023 | H | -0.63319 | -4.79244 | -0.6596 |
| C | -4.64859 | -1.46213 | 0.56809 | H | -0.06897 | -4.36116 | -2.26539 |
| C | -5.62182 | -1.21431 | 1.533636 | H | 0.382347 | -1.83972 | -2.3577 |
| C | -6.59634 | -2.17911 | 1.866529 | H | 7.23017 | -1.45052 | 3.08164 |
| C | -6.61823 | -3.40549 | 1.237747 | H | 7.240982 | -1.8289 | -1.19718 |
| C | -5.58876 | -3.74888 | 0.320707 | H | 5.213272 | -1.29667 | -2.39082 |
| C | -4.55986 | -2.79147 | 0.040642 | H | 3.983103 | -0.20314 | -1.71621 |
| C | -5.52031 | -5.02329 | -0.29939 | H | 5.539585 | 0.438751 | -2.26017 |
| C | -4.45795 | -5.35207 | -1.11765 | H | 3.991028 | -0.39026 | 3.473273 |
| C | -3.3881 | -4.45291 | -1.31528 | H | 5.603476 | -0.21508 | 4.20384 |
| C | -3.4266 | -3.18966 | -0.72062 | H | 4.860343 | 1.139428 | 3.310703 |
| C | -2.19558 | -4.80094 | -2.1697 | H | 8.348089 | -3.61628 | 1.385094 |
| C | -0.91369 | -4.22992 | -1.57236 | H | 9.108683 | -2.71259 | 0.05663 |
| C | -1.06247 | -2.77743 | -1.20131 | H | 9.351719 | -2.20044 | 1.746794 |
| C | -2.26552 | -2.28912 | -0.79286 | H | 2.654946 | -3.55261 | 2.372452 |
| C | 0.172962 | -1.92574 | -1.27693 | H | 4.507005 | -3.74147 | -1.49328 |
| C | 5.048309 | -0.1342 | 0.796222 | H | 3.471851 | -3.01292 | -3.39037 |
| C | 5.626789 | -0.45434 | 2.053117 | H | 1.716144 | -3.25014 | -3.4651 |
| C | 6.787099 | -1.22627 | 2.106587 | H | 2.380301 | -1.62812 | -3.20079 |
| C | 7.393638 | -1.72701 | 0.945318 | H | -0.61344 | -3.02571 | 1.369111 |
| C | 6.798308 | -1.42904 | -0.27992 | H | 0.544051 | -2.75932 | 2.69404 |
| C | 5.637571 | -0.64562 | -0.38198 | H | 0.061514 | -1.41946 | 1.626295 |
| C | 5.058107 | -0.4141 | -1.75445 | H | 4.731729 | -5.23087 | 1.69237 |
| C | 4.994116 | 0.046074 | 3.326795 | H | 5.734449 | -4.53084 | 0.393872 |
| C | 8.615478 | -2.60419 | 1.033838 | H | 5.405449 | -3.59394 | 1.86386 |
| C | 1.404477 | -2.58991 | -0.65325 | H | 3.022328 | 0.297635 | 1.460761 |

| **INT9** | | | | | | | |
| --- | --- | --- | --- | --- | --- | --- | --- |
| C | -5.07504 | 1.557526 | 0.478905 | C | 1.59517 | -2.85162 | 0.494013 |
| C | -5.178 | 2.83058 | 1.117 | C | 2.778564 | -3.40911 | 0.993894 |
| C | -3.76158 | 0.947204 | 0.321461 | C | 3.875261 | -3.69284 | 0.172988 |
| C | -2.63254 | 1.800215 | 0.381774 | C | 3.774618 | -3.36307 | -1.18116 |
| C | -2.76105 | 3.028101 | 1.115528 | C | 2.610935 | -2.80288 | -1.72566 |
| C | -3.97717 | 3.492635 | 1.524546 | C | 2.588915 | -2.46503 | -3.1991 |
| C | -3.54821 | -0.4721 | 0.131964 | C | 0.459204 | -2.58371 | 1.451563 |
| C | -2.33623 | -0.91661 | -0.42712 | C | 5.100724 | -4.37436 | 0.720322 |
| C | -1.23841 | -0.01391 | -0.56571 | H | -1.8605 | 3.581798 | 1.37407 |
| C | -1.37519 | 1.364747 | -0.19844 | H | -4.04888 | 4.40848 | 2.115493 |
| C | 0.037927 | -0.51315 | -0.98365 | H | 2.113634 | -0.12092 | -1.11653 |
| C | 1.138118 | 0.294776 | -0.88459 | H | -2.42703 | 3.651501 | -1.38657 |
| C | 1.033589 | 1.671961 | -0.5474 | H | -2.57915 | 6.076421 | -1.79412 |
| C | -0.27455 | 2.247895 | -0.44211 | H | -0.6177 | 7.550759 | -1.33987 |
| C | -0.38107 | 3.681016 | -0.69956 | H | 1.759067 | 7.788867 | -0.70524 |
| C | -1.55831 | 4.275413 | -1.18422 | H | 3.927132 | 6.769636 | -0.07813 |
| C | -1.64276 | 5.653278 | -1.42626 | H | 5.333539 | 4.788935 | -0.09658 |
| C | -0.54941 | 6.470869 | -1.19018 | H | 4.585019 | 4.043073 | 1.319615 |
| C | 0.685331 | 5.906108 | -0.78655 | H | 4.769507 | 2.827285 | -1.49397 |
| C | 0.784659 | 4.494631 | -0.59502 | H | 5.60436 | 2.266706 | -0.03118 |
| C | 1.839207 | 6.706419 | -0.5793 | H | -6.51424 | 4.3852 | 1.814531 |
| C | 3.050995 | 6.136356 | -0.23311 | H | -8.56482 | 3.294592 | 0.924025 |
| C | 3.187021 | 4.739977 | -0.11765 | H | -8.3708 | 1.158467 | -0.36236 |
| C | 2.059452 | 3.910374 | -0.31692 | H | -6.19059 | 0.05705 | -0.62699 |
| C | 4.516229 | 4.123184 | 0.217613 | H | -5.4366 | -0.23569 | 2.1311 |
| C | 4.6814 | 2.731686 | -0.39761 | H | -7.07332 | -1.92421 | 2.848561 |
| C | 3.486546 | 1.875868 | -0.04903 | H | -7.14775 | -4.15702 | 1.749991 |
| C | 2.186364 | 2.476245 | -0.30365 | H | -6.14102 | -5.79565 | 0.168797 |
| C | 3.591908 | 0.669227 | 0.591484 | H | -4.32445 | -6.40349 | -1.40849 |
| C | -6.45119 | 3.42502 | 1.296892 | H | -2.06897 | -5.95156 | -2.2602 |
| C | -7.58865 | 2.822398 | 0.794509 | H | -2.38332 | -4.46133 | -3.16094 |
| C | -7.48062 | 1.60938 | 0.081323 | H | -0.4903 | -4.79763 | -0.76002 |
| C | -6.25409 | 0.988359 | -0.06634 | H | -0.05192 | -4.38503 | -2.40856 |
| C | -4.48873 | -1.48154 | 0.647273 | H | 0.374057 | -1.87339 | -2.55399 |
| C | -5.41812 | -1.21284 | 1.65439 | H | 6.252564 | -1.77803 | 3.453294 |
| C | -6.3561 | -2.17834 | 2.065584 | H | 7.568402 | -1.45174 | -0.62355 |
| C | -6.39016 | -3.42246 | 1.466936 | H | 5.847822 | -0.88468 | -2.27044 |
| C | -5.40998 | -3.7809 | 0.504563 | H | 4.483684 | 0.172188 | -1.85256 |
| C | -4.40702 | -2.82307 | 0.14678 | H | 6.135745 | 0.820073 | -1.89952 |
| C | -5.36469 | -5.07048 | -0.08561 | H | 2.961076 | -0.85524 | 2.942594 |
| C | -4.34685 | -5.40976 | -0.95437 | H | 4.239215 | -0.86843 | 4.177093 |
| C | -3.30032 | -4.50502 | -1.23536 | H | 3.761774 | 0.659004 | 3.398189 |
| C | -3.31834 | -3.22764 | -0.66935 | H | 7.999761 | -3.52722 | 2.050444 |
| C | -2.1585 | -4.86075 | -2.15306 | H | 8.999532 | -2.53666 | 0.96041 |
| C | -0.84724 | -4.25631 | -1.65853 | H | 8.814886 | -2.08471 | 2.67527 |
| C | -0.99221 | -2.80154 | -1.30027 | H | 2.84032 | -3.63807 | 2.061382 |
| C | -2.17413 | -2.3189 | -0.83073 | H | 4.626193 | -3.55286 | -1.83997 |
| C | 0.225301 | -1.93731 | -1.46221 | H | 3.562575 | -2.67893 | -3.66096 |
| C | 4.788462 | -0.12185 | 0.843788 | H | 1.831852 | -3.04775 | -3.74937 |
| C | 4.964249 | -0.65785 | 2.15294 | H | 2.367828 | -1.39946 | -3.37805 |
| C | 6.107981 | -1.39565 | 2.439094 | H | -0.40788 | -3.23282 | 1.254032 |
| C | 7.076913 | -1.66928 | 1.459442 | H | 0.77704 | -2.76162 | 2.488494 |
| C | 6.852087 | -1.20023 | 0.163461 | H | 0.094336 | -1.54787 | 1.383095 |
| C | 5.727149 | -0.4361 | -0.17192 | H | 4.986028 | -5.47099 | 0.673467 |
| C | 5.53086 | -0.05739 | -1.61876 | H | 5.999027 | -4.10985 | 0.145277 |
| C | 3.933103 | -0.41516 | 3.224428 | H | 5.275049 | -4.10383 | 1.771161 |
| C | 8.288948 | -2.49299 | 1.797663 | H | 2.674298 | 0.267706 | 1.028459 |
| C | 1.501578 | -2.56155 | -0.88674 |  |  |  |  |

| **TS4** | | | | | | | |
| --- | --- | --- | --- | --- | --- | --- | --- |
| C | 4.642977 | 1.486349 | 1.370245 | C | -2.77008 | -3.30935 | -0.32021 |
| C | 4.749605 | 2.863923 | 1.746655 | C | -3.77264 | -3.73591 | -1.20325 |
| C | 3.49454 | 1.05068 | 0.600873 | C | -3.74433 | -3.42674 | -2.56489 |
| C | 2.404353 | 1.939492 | 0.427679 | C | -2.65761 | -2.68215 | -3.04175 |
| C | 2.520594 | 3.293068 | 0.883566 | C | -1.63219 | -2.23845 | -2.19949 |
| C | 3.664641 | 3.748335 | 1.470736 | C | -0.4831 | -1.46387 | -2.80041 |
| C | 3.362686 | -0.30707 | 0.084907 | C | -2.86401 | -3.72158 | 1.128593 |
| C | 2.091583 | -0.86737 | 0.085731 | C | -4.83621 | -3.87841 | -3.4993 |
| C | 0.919311 | -0.00909 | 0.109147 | H | 1.669927 | 3.962427 | 0.790731 |
| C | 1.137131 | 1.426215 | -0.05348 | H | 3.734005 | 4.785288 | 1.807122 |
| C | -0.36085 | -0.56835 | 0.092841 | H | -2.28293 | -0.13746 | -0.68141 |
| C | -1.48584 | 0.304972 | -0.07685 | H | 2.164787 | 3.506935 | -1.80416 |
| C | -1.20808 | 1.694134 | -0.36615 | H | 2.190897 | 5.775951 | -2.77251 |
| C | 0.070044 | 2.240948 | -0.53591 | H | 0.186909 | 7.237358 | -2.65202 |
| C | 0.127471 | 3.606241 | -1.11913 | H | -2.22968 | 7.524568 | -2.0096 |
| C | 1.261834 | 4.114735 | -1.74561 | H | -4.32473 | 6.605532 | -1.0819 |
| C | 1.27592 | 5.412713 | -2.30027 | H | -5.46861 | 4.693353 | 0.222354 |
| C | 0.159343 | 6.224805 | -2.24304 | H | -5.20649 | 3.521615 | -1.06498 |
| C | -1.04588 | 5.73908 | -1.67 | H | -4.4002 | 3.285678 | 1.89161 |
| C | -1.06364 | 4.41484 | -1.13776 | H | -5.43313 | 2.215067 | 0.957073 |
| C | -2.24654 | 6.505421 | -1.61608 | H | 5.954397 | 4.373422 | 2.723575 |
| C | -3.42168 | 5.990427 | -1.09525 | H | 7.722936 | 2.780144 | 3.444327 |
| C | -3.46924 | 4.664463 | -0.60545 | H | 7.447317 | 0.336981 | 2.975668 |
| C | -2.29743 | 3.916982 | -0.64121 | H | 5.534622 | -0.4744 | 1.688477 |
| C | -4.74216 | 3.962706 | -0.16272 | H | 5.758774 | 0.600968 | -0.89039 |
| C | -4.53277 | 2.843466 | 0.884603 | H | 7.643206 | -0.75863 | -1.73438 |
| C | -3.30627 | 2.016587 | 0.60005 | H | 7.516144 | -3.24208 | -1.70244 |
| C | -2.3241 | 2.552734 | -0.1907 | H | 6.203239 | -5.31599 | -1.25583 |
| C | -2.90625 | 0.76144 | 1.258097 | H | 4.082912 | -6.39447 | -0.55586 |
| C | 5.882464 | 3.313127 | 2.470198 | H | 1.66516 | -6.19997 | -0.15865 |
| C | 6.857486 | 2.426292 | 2.879949 | H | 1.706573 | -5.18849 | 1.291769 |
| C | 6.709066 | 1.049632 | 2.601983 | H | 0.408672 | -4.49488 | -1.40799 |
| C | 5.63213 | 0.592831 | 1.868763 | H | -0.39738 | -4.74209 | 0.127913 |
| C | 4.488874 | -1.08111 | -0.45153 | H | -0.93779 | -2.28031 | 1.151274 |
| C | 5.659708 | -0.48387 | -0.91243 | H | -6.52952 | -2.0643 | 0.468175 |
| C | 6.738258 | -1.25967 | -1.38461 | H | -4.80161 | -2.05794 | 4.403143 |
| C | 6.665953 | -2.63777 | -1.37771 | H | -2.91758 | -0.85364 | 4.919724 |
| C | 5.473584 | -3.29314 | -0.96645 | H | -1.78573 | -0.48306 | 3.605896 |
| C | 4.353062 | -2.5018 | -0.55888 | H | -2.89793 | 0.766137 | 4.198981 |
| C | 5.346978 | -4.70677 | -0.95732 | H | -5.85529 | -0.55588 | -1.17229 |
| C | 4.162257 | -5.3047 | -0.57728 | H | -4.63036 | 0.698874 | -0.90328 |
| C | 3.029687 | -4.52956 | -0.24391 | H | -4.14368 | -0.96457 | -1.28998 |
| C | 3.113587 | -3.13648 | -0.26769 | H | -7.68838 | -3.19732 | 2.303752 |
| C | 1.728272 | -5.1571 | 0.184895 | H | -6.34462 | -4.21587 | 2.861711 |
| C | 0.53013 | -4.35618 | -0.31542 | H | -7.08603 | -3.05697 | 3.979002 |
| C | 0.67596 | -2.87848 | -0.05536 | H | -4.6 | -4.33103 | -0.80852 |
| C | 1.914032 | -2.30963 | -0.05522 | H | -2.59814 | -2.449 | -4.10857 |
| C | -0.58368 | -2.06537 | 0.125006 | H | -0.47501 | -1.57242 | -3.89374 |
| C | -3.87797 | -0.26489 | 1.642491 | H | -0.55018 | -0.3854 | -2.58192 |
| C | -4.85449 | -0.74681 | 0.729004 | H | 0.489149 | -1.80559 | -2.41813 |
| C | -5.78507 | -1.68819 | 1.174266 | H | -2.86907 | -2.85808 | 1.807226 |
| C | -5.79061 | -2.16895 | 2.489264 | H | -3.78944 | -4.2828 | 1.31193 |
| C | -4.81391 | -1.69035 | 3.37401 | H | -2.02316 | -4.36748 | 1.428806 |
| C | -3.84933 | -0.76537 | 2.975477 | H | -5.36789 | -3.01598 | -3.93457 |
| C | -2.8115 | -0.30876 | 3.972195 | H | -4.42474 | -4.45803 | -4.34115 |
| C | -4.87115 | -0.35624 | -0.72714 | H | -5.57648 | -4.50703 | -2.9843 |
| C | -6.78575 | -3.20596 | 2.931259 | H | -2.13189 | 0.934645 | 2.008731 |
| C | -1.70117 | -2.53349 | -0.81602 |  |  |  |  |

| **INT10** | | | | | | | |
| --- | --- | --- | --- | --- | --- | --- | --- |
| C | 4.605144 | 1.536428 | 1.251073 | C | -2.74905 | -3.34844 | -0.37361 |
| C | 4.725385 | 2.932014 | 1.548241 | C | -3.68764 | -3.79276 | -1.31575 |
| C | 3.451331 | 1.067483 | 0.511697 | C | -3.59244 | -3.47427 | -2.6719 |
| C | 2.361433 | 1.957354 | 0.295952 | C | -2.49841 | -2.70323 | -3.08296 |
| C | 2.498856 | 3.337952 | 0.665261 | C | -1.53517 | -2.23595 | -2.18167 |
| C | 3.648788 | 3.811804 | 1.221754 | C | -0.37531 | -1.43496 | -2.72485 |
| C | 3.320355 | -0.3078 | 0.054581 | C | -2.91816 | -3.78599 | 1.059869 |
| C | 2.0487 | -0.86563 | 0.085098 | C | -4.62941 | -3.93303 | -3.66308 |
| C | 0.870511 | -0.00349 | 0.083103 | H | 1.6564 | 4.0104 | 0.538228 |
| C | 1.097041 | 1.431946 | -0.148 | H | 3.73078 | 4.865564 | 1.497196 |
| C | -0.39701 | -0.55567 | 0.1595 | H | -2.17265 | -0.11256 | -0.80162 |
| C | -1.57845 | 0.332171 | 0.016816 | H | 2.021736 | 3.484302 | -2.10195 |
| C | -1.23358 | 1.707794 | -0.39589 | H | 2.010507 | 5.79201 | -2.98884 |
| C | 0.023478 | 2.231 | -0.65851 | H | 0.081436 | 7.300573 | -2.59767 |
| C | 0.055023 | 3.602485 | -1.23925 | H | -2.24897 | 7.631955 | -1.66044 |
| C | 1.139908 | 4.107195 | -1.94586 | H | -4.24696 | 6.733584 | -0.52681 |
| C | 1.135864 | 5.429821 | -2.44471 | H | -5.28303 | 4.757495 | 0.837904 |
| C | 0.058372 | 6.269839 | -2.23641 | H | -5.2617 | 3.677463 | -0.55186 |
| C | -1.09957 | 5.79427 | -1.56334 | H | -4.06043 | 3.170636 | 2.235543 |
| C | -1.10918 | 4.442566 | -1.10747 | H | -5.2596 | 2.221024 | 1.368879 |
| C | -2.25818 | 6.591885 | -1.32584 | H | 5.946974 | 4.481735 | 2.43569 |
| C | -3.38195 | 6.086336 | -0.69164 | H | 7.700189 | 2.91372 | 3.242995 |
| C | -3.42771 | 4.732181 | -0.28107 | H | 7.40162 | 0.45104 | 2.91342 |
| C | -2.3034 | 3.954603 | -0.51786 | H | 5.47939 | -0.41262 | 1.67313 |
| C | -4.65182 | 4.036014 | 0.298657 | H | 5.713672 | 0.541734 | -0.98546 |
| C | -4.3489 | 2.822543 | 1.222735 | H | 7.598521 | -0.86793 | -1.74297 |
| C | -3.22226 | 1.989916 | 0.696531 | H | 7.475226 | -3.3441 | -1.54942 |
| C | -2.33362 | 2.56577 | -0.15916 | H | 6.163673 | -5.38757 | -0.96406 |
| C | -2.70601 | 0.635254 | 1.150541 | H | 4.046287 | -6.41793 | -0.18828 |
| C | 5.863202 | 3.409805 | 2.242596 | H | 1.626142 | -6.19664 | 0.192948 |
| C | 6.830359 | 2.536748 | 2.700756 | H | 1.668328 | -5.09619 | 1.57689 |
| C | 6.669254 | 1.14825 | 2.501319 | H | 0.385926 | -4.56445 | -1.16728 |
| C | 5.587173 | 0.661843 | 1.79528 | H | -0.43328 | -4.73019 | 0.373219 |
| C | 4.447505 | -1.11101 | -0.43675 | H | -1.04676 | -2.24794 | 1.206537 |
| C | 5.615839 | -0.54261 | -0.93751 | H | -6.46156 | -2.00799 | 0.197185 |
| C | 6.694861 | -1.34618 | -1.35969 | H | -4.93541 | -2.08968 | 4.210688 |
| C | 6.624564 | -2.72133 | -1.26324 | H | -3.04011 | -0.97724 | 4.82871 |
| C | 5.433938 | -3.34979 | -0.80809 | H | -1.80643 | -0.67293 | 3.592802 |
| C | 4.313827 | -2.53464 | -0.45086 | H | -2.8915 | 0.632938 | 4.106418 |
| C | 5.307978 | -4.76005 | -0.7044 | H | -5.66827 | -0.49322 | -1.36954 |
| C | 4.124929 | -5.33201 | -0.28203 | H | -4.42537 | 0.716426 | -1.00322 |
| C | 2.992271 | -4.53688 | 0.001953 | H | -3.96403 | -0.93533 | -1.47819 |
| C | 3.075678 | -3.14879 | -0.11435 | H | -7.79797 | -3.03399 | 2.016602 |
| C | 1.691052 | -5.13447 | 0.470359 | H | -6.54065 | -4.1873 | 2.504515 |
| C | 0.495007 | -4.36595 | -0.08294 | H | -7.186 | -3.03972 | 3.69252 |
| C | 0.636071 | -2.87545 | 0.092892 | H | -4.52035 | -4.41057 | -0.97072 |
| C | 1.876663 | -2.3095 | 0.045615 | H | -2.3823 | -2.46498 | -4.14376 |
| C | -0.61927 | -2.04841 | 0.204207 | H | -0.32881 | -1.52365 | -3.81881 |
| C | -3.75789 | -0.3844 | 1.49432 | H | -0.46278 | -0.36013 | -2.49542 |
| C | -4.71999 | -0.79831 | 0.544157 | H | 0.587739 | -1.7738 | -2.31859 |
| C | -5.72654 | -1.68628 | 0.93973 | H | -2.92313 | -2.93899 | 1.756862 |
| C | -5.82516 | -2.16765 | 2.249064 | H | -3.8699 | -4.31595 | 1.191137 |
| C | -4.87471 | -1.73229 | 3.179489 | H | -2.11668 | -4.47061 | 1.380298 |
| C | -3.84124 | -0.85835 | 2.826793 | H | -5.27735 | -3.09441 | -3.97011 |
| C | -2.84507 | -0.44661 | 3.886983 | H | -4.16206 | -4.32919 | -4.57786 |
| C | -4.68869 | -0.34528 | -0.89463 | H | -5.27555 | -4.71576 | -3.24073 |
| C | -6.89732 | -3.15075 | 2.636984 | H | -2.10179 | 0.837494 | 2.045776 |
| C | -1.67779 | -2.53618 | -0.80437 |  |  |  |  |

| **INT11** | | | | | | | |
| --- | --- | --- | --- | --- | --- | --- | --- |
| C | 4.988295 | 1.522564 | 0.671531 | C | -4.8824 | -0.92634 | 1.656801 |
| C | 5.212609 | 2.915449 | 0.910683 | C | -4.78312 | -0.68488 | 3.146272 |
| C | 3.66399 | 1.075497 | 0.256275 | C | -3.36639 | 0.200023 | -1.68489 |
| C | 2.585826 | 1.988114 | 0.303587 | C | -7.27459 | -2.78564 | -0.68009 |
| C | 2.837951 | 3.349407 | 0.668346 | C | -1.62232 | -2.53904 | 0.018003 |
| C | 4.101812 | 3.809215 | 0.898223 | C | -2.67072 | -3.29546 | 0.589242 |
| C | 3.358195 | -0.31123 | -0.03624 | C | -3.56172 | -3.97989 | -0.24529 |
| C | 2.087104 | -0.78423 | 0.293173 | C | -3.45219 | -3.943 | -1.63573 |
| C | 0.967164 | 0.122615 | 0.406299 | C | -2.39981 | -3.20511 | -2.18259 |
| C | 1.21762 | 1.515214 | 0.140261 | C | -1.47615 | -2.51376 | -1.38898 |
| C | -0.35103 | -0.39069 | 0.683996 | C | -0.34326 | -1.80016 | -2.08654 |
| C | -1.39469 | 0.532164 | 0.738321 | C | -2.86712 | -3.43684 | 2.080068 |
| C | -1.12287 | 1.849826 | 0.30816 | C | -4.4587 | -4.6327 | -2.5185 |
| C | 0.098509 | 2.372495 | -0.10207 | H | 1.998688 | 4.028463 | 0.786847 |
| C | 0.04853 | 3.727932 | -0.72417 | H | 4.267329 | 4.857879 | 1.157461 |
| C | 1.060287 | 4.232975 | -1.53509 | H | 1.937197 | 3.621485 | -1.74383 |
| C | 0.994438 | 5.533788 | -2.08385 | H | 1.827629 | 5.89005 | -2.69432 |
| C | -0.09198 | 6.352758 | -1.85471 | H | -0.1276 | 7.36381 | -2.26801 |
| C | -1.20302 | 5.8642 | -1.11482 | H | -2.43944 | 7.631825 | -1.33052 |
| C | -1.13896 | 4.542829 | -0.57192 | H | -4.43799 | 6.662821 | -0.25029 |
| C | -2.40311 | 6.612158 | -0.93829 | H | -5.45545 | 4.666033 | 1.096113 |
| C | -3.52332 | 6.068525 | -0.33058 | H | -5.26507 | 3.652051 | -0.32991 |
| C | -3.50703 | 4.737992 | 0.146621 | H | -4.4182 | 3.029966 | 2.546496 |
| C | -2.3164 | 4.029274 | 0.039601 | H | -5.41627 | 2.083094 | 1.441389 |
| C | -4.75341 | 3.981685 | 0.594859 | H | 6.64984 | 4.450032 | 1.421025 |
| C | -4.52367 | 2.726991 | 1.48503 | H | 8.535041 | 2.85794 | 1.758277 |
| C | -3.28439 | 1.98685 | 1.076511 | H | 8.101303 | 0.394708 | 1.577329 |
| C | -2.29682 | 2.656724 | 0.448752 | H | 5.90599 | -0.43419 | 0.922767 |
| C | -2.85466 | 0.532183 | 1.282753 | H | 5.423208 | 0.259109 | -1.74587 |
| C | 6.504841 | 3.376768 | 1.272498 | H | 7.055275 | -1.33824 | -2.70726 |
| C | 7.545333 | 2.493623 | 1.472879 | H | 6.966536 | -3.74946 | -2.10196 |
| C | 7.304332 | 1.108035 | 1.354423 | H | 5.809833 | -5.62076 | -0.88949 |
| C | 6.061814 | 0.639221 | 0.97243 | H | 3.926839 | -6.40436 | 0.524935 |
| C | 4.309722 | -1.23402 | -0.67244 | H | 1.67822 | -6.00056 | 1.445749 |
| C | 5.326356 | -0.80293 | -1.51977 | H | 2.002346 | -4.63387 | 2.525655 |
| C | 6.264289 | -1.71255 | -2.05311 | H | 0.17478 | -4.63764 | 0.056087 |
| C | 6.21225 | -3.05302 | -1.72751 | H | -0.29814 | -4.43158 | 1.733976 |
| C | 5.159502 | -3.55006 | -0.91097 | H | -0.94703 | -1.96386 | 1.956569 |
| C | 4.163242 | -2.63647 | -0.44103 | H | -5.44268 | -1.391 | -2.15265 |
| C | 5.046895 | -4.91736 | -0.5466 | H | -6.64576 | -2.13664 | 1.898732 |
| C | 3.991576 | -5.35304 | 0.231427 | H | -5.56226 | -1.24572 | 3.681603 |
| C | 2.964074 | -4.46953 | 0.629275 | H | -3.81285 | -0.98974 | 3.568393 |
| C | 3.027074 | -3.12577 | 0.259649 | H | -4.91421 | 0.381131 | 3.394905 |
| C | 1.795748 | -4.90666 | 1.472224 | H | -3.57427 | -0.2036 | -2.68519 |
| C | 0.511343 | -4.21706 | 1.02325 | H | -3.58248 | 1.280772 | -1.69843 |
| C | 0.679894 | -2.72638 | 0.85212 | H | -2.29311 | 0.097722 | -1.49583 |
| C | 1.890772 | -2.2193 | 0.498225 | H | -7.60389 | -2.49918 | -1.69046 |
| C | -0.56784 | -1.8784 | 0.925166 | H | -6.94278 | -3.83738 | -0.73276 |
| C | -3.96223 | -0.36647 | 0.74079 | H | -8.14793 | -2.75294 | -0.01075 |
| C | -4.20095 | -0.5012 | -0.64578 | H | -4.37613 | -4.54803 | 0.211398 |
| C | -5.28426 | -1.27433 | -1.07724 | H | -2.28343 | -3.16762 | -3.26959 |
| C | -6.16152 | -1.89831 | -0.18737 | H | -0.46639 | -1.86323 | -3.1767 |
| C | -5.95221 | -1.69283 | 1.178451 | H | -0.28271 | -0.7369 | -1.81699 |

| **1** | | | | | | | |
| --- | --- | --- | --- | --- | --- | --- | --- |
| C | 3.620725 | 0.692007 | 0.600023 | C | -4.59766 | -3.57754 | -0.90936 |
| C | -0.95118 | 1.947256 | -0.35987 | C | 5.588833 | -0.0714 | 1.998698 |
| C | 3.371726 | -0.56959 | -0.02986 | H | 5.35963 | -1.11103 | 1.776003 |
| C | 2.014356 | -0.95154 | -0.21904 | C | -3.51629 | -0.58747 | 2.28282 |
| C | 2.631681 | 1.709882 | 0.437273 | C | -4.25752 | 4.37898 | -0.07785 |
| C | -2.03696 | 2.841607 | -0.36986 | H | -5.15637 | 4.99094 | 0.035876 |
| C | 0.95885 | 0.016291 | -0.14518 | C | -3.06551 | 5.006836 | -0.53072 |
| C | 0.346184 | 2.380896 | -0.50518 | C | -3.89659 | -3.7542 | 0.287091 |
| C | -3.19467 | 2.191198 | 0.070344 | H | -4.32595 | -4.38379 | 1.07138 |
| C | 1.697257 | -2.31726 | -0.51864 | C | 1.823973 | 5.59326 | -1.96025 |
| C | 4.420799 | -1.45762 | -0.5194 | H | 2.777001 | 5.945328 | -2.36179 |
| C | 2.901855 | 3.020926 | 0.963002 | C | 0.742892 | 6.471843 | -1.88176 |
| H | 2.138801 | 3.789145 | 0.880316 | H | 0.853676 | 7.510981 | -2.20235 |
| C | -0.44326 | -0.42979 | -0.14522 | C | -4.47435 | -1.36396 | 2.94038 |
| C | 5.725816 | -1.00981 | -0.79342 | H | -4.2643 | -1.7108 | 3.956444 |
| H | 5.969008 | 0.042798 | -0.65887 | C | 4.06601 | 3.311057 | 1.607781 |
| C | -1.44692 | 0.629498 | 0.000601 | H | 4.240587 | 4.318021 | 1.994954 |
| C | -2.10884 | -2.31709 | -0.48619 | C | -0.50323 | 6.017115 | -1.41239 |
| C | 1.348881 | 1.389596 | -0.12003 | C | 2.429426 | -4.60998 | -1.08092 |
| C | 4.09948 | -2.8137 | -0.82291 | C | -1.68035 | 6.854601 | -1.32758 |
| C | 4.769292 | 0.955749 | 1.461715 | H | -1.57707 | 7.903026 | -1.61953 |
| C | 6.719186 | -1.88944 | -1.22635 | C | -2.20327 | -1.28561 | -2.8063 |
| H | 7.724622 | -1.50916 | -1.42041 | H | -2.12806 | -0.23039 | -2.50033 |
| C | 0.516445 | 3.763547 | -0.99951 | H | -2.82109 | -1.33427 | -3.71428 |
| C | -3.77739 | -0.13756 | 0.965623 | H | -1.18376 | -1.61215 | -3.0656 |
| C | -1.90687 | 4.190136 | -0.63602 | C | 1.069759 | -5.002 | -1.1161 |
| C | 1.719254 | 4.266388 | -1.52545 | H | 0.824722 | -6.03923 | -1.35758 |
| H | 2.589213 | 3.613691 | -1.59759 | C | -1.90351 | -3.45997 | 1.780553 |
| C | 5.023639 | 2.289571 | 1.900524 | H | -1.28779 | -4.36748 | 1.657968 |
| C | 6.438324 | -3.24053 | -1.40728 | H | -2.59808 | -3.63482 | 2.614148 |
| H | 7.222288 | -3.93371 | -1.72191 | H | -1.22041 | -2.65033 | 2.064164 |
| C | -0.6163 | 4.656349 | -1.01195 | C | 0.061868 | -4.09314 | -0.88607 |
| C | 4.783235 | -5.09172 | -1.48128 | H | -0.97577 | -4.41622 | -0.9498 |
| H | 5.58096 | -5.78495 | -1.75943 | C | 6.15128 | 2.565372 | 2.71389 |
| C | -2.81766 | 0.774304 | 0.312916 | H | 6.33629 | 3.599939 | 3.014271 |
| C | 5.127418 | -3.72128 | -1.2312 | C | -5.95409 | -4.20096 | -1.10635 |
| C | -0.7228 | -1.78053 | -0.35186 | H | -6.73734 | -3.57784 | -0.64022 |
| C | -2.79184 | -2.13304 | -1.70686 | H | -6.01497 | -5.19662 | -0.64026 |
| C | -4.32903 | 3.009586 | 0.244624 | H | -6.20448 | -4.30336 | -2.17287 |
| H | -5.26508 | 2.601765 | 0.632635 | C | -5.69283 | -1.70196 | 2.340413 |
| C | 0.340528 | -2.72981 | -0.5807 | C | -5.36602 | 0.056006 | -1.02594 |
| C | 2.741943 | -3.25655 | -0.77464 | H | -5.89895 | 1.020157 | -0.9892 |
| C | -4.0216 | -2.77247 | -1.89694 | H | -6.02067 | -0.66283 | -1.53876 |
| H | -4.54682 | -2.63158 | -2.84573 | H | -4.47203 | 0.206264 | -1.64332 |
| C | -5.01779 | -0.43581 | 0.357698 | C | -5.94748 | -1.21347 | 1.056487 |
| C | -2.6558 | -3.151 | 0.511472 | H | -6.89818 | -1.45606 | 0.572443 |
| C | -4.59766 | -3.57754 | -0.90936 | C | 3.489094 | -5.51965 | -1.39922 |
| C | 5.588833 | -0.0714 | 1.998698 | H | 3.234414 | -6.56154 | -1.60862 |
| H | 5.35963 | -1.11103 | 1.776003 | C | 6.976129 | 1.548762 | 3.153598 |
| C | -3.51629 | -0.58747 | 2.28282 | H | 7.838032 | 1.770053 | 3.787296 |
| C | 3.620725 | 0.692007 | 0.600023 | C | -2.89674 | 6.387774 | -0.89598 |
| C | -0.95118 | 1.947256 | -0.35987 | H | -3.75224 | 7.066081 | -0.83925 |
| C | 3.371726 | -0.56959 | -0.02986 | C | -2.24968 | -0.19333 | 3.000778 |
| C | 2.014356 | -0.95154 | -0.21904 | H | -1.34991 | -0.55247 | 2.481711 |
| C | 2.631681 | 1.709882 | 0.437273 | H | -2.23697 | -0.596 | 4.023706 |
| C | -2.03696 | 2.841607 | -0.36986 | H | -2.15391 | 0.902678 | 3.058502 |
| C | 0.95885 | 0.016291 | -0.14518 | C | 6.666254 | 0.214409 | 2.816818 |
| C | 0.346184 | 2.380896 | -0.50518 | H | 7.272716 | -0.60327 | 3.213195 |
| C | 2.741943 | -3.25655 | -0.77464 | C | -6.68045 | -2.60187 | 3.03672 |
| C | -4.0216 | -2.77247 | -1.89694 | H | -6.5954 | -2.52943 | 4.13163 |
| H | -4.54682 | -2.63158 | -2.84573 | H | -6.50674 | -3.65847 | 2.764587 |
| C | -5.01779 | -0.43581 | 0.357698 | H | -7.71728 | -2.36194 | 2.755073 |
| C | -2.6558 | -3.151 | 0.511472 |  |  |  |  |

**Chiral optical properties part**:

a. Optimized structures with no imaginary frequencies

| **1** (B3LYP-D3(BJ)/6-31G(d), Solvent = Toluene, PCM) | | | | | | | |
| --- | --- | --- | --- | --- | --- | --- | --- |
| C | 3.613922 | 0.695542 | 0.60142 | H | -4.31805 | -4.39625 | 1.032 |
| C | -0.953631 | 1.938647 | -0.366294 | C | 1.811873 | 5.582791 | -1.96493 |
| C | 3.368164 | -0.565591 | -0.025949 | H | 2.759069 | 5.93409 | -2.36351 |
| C | 2.01355 | -0.949231 | -0.217213 | C | 0.730281 | 6.457993 | -1.88965 |
| C | 2.624321 | 1.709747 | 0.43632 | H | 0.838929 | 7.490815 | -2.2104 |
| C | -2.03889 | 2.83099 | -0.376071 | C | -4.4503 | -1.36897 | 2.953713 |
| C | 0.956999 | 0.014538 | -0.142401 | H | -4.23383 | -1.71769 | 3.961452 |
| C | 0.340842 | 2.373855 | -0.507388 | C | 4.052296 | 3.314566 | 1.601175 |
| C | -3.191823 | 2.182572 | 0.072164 | H | 4.223758 | 4.316336 | 1.986215 |
| C | 1.699883 | -2.313654 | -0.517745 | C | -0.51257 | 6.001942 | -1.41998 |
| C | 4.418365 | -1.450841 | -0.513415 | C | 2.436843 | -4.60108 | -1.08054 |
| C | 2.890456 | 3.021074 | 0.958732 | C | -1.689 | 6.837557 | -1.33507 |
| H | 2.129355 | 3.782244 | 0.873156 | H | -1.58709 | 7.879533 | -1.62744 |
| C | -0.442935 | -0.434643 | -0.146011 | C | -2.19948 | -1.26945 | -2.81326 |
| C | 5.720197 | -1.000447 | -0.787173 | H | -2.15441 | -0.216 | -2.51539 |
| H | 5.959884 | 0.046435 | -0.651104 | H | -2.80423 | -1.33825 | -3.72225 |
| C | -1.446139 | 0.62354 | 0.0037 | H | -1.17622 | -1.57473 | -3.05919 |
| C | -2.103809 | -2.320645 | -0.499188 | C | 1.080441 | -4.99581 | -1.11754 |
| C | 1.344376 | 1.386519 | -0.120688 | H | 0.839793 | -6.0276 | -1.35938 |
| C | 4.099997 | -2.804779 | -0.818148 | C | -1.90103 | -3.48856 | 1.756596 |
| C | 4.760654 | 0.963318 | 1.461778 | H | -1.28547 | -4.38822 | 1.626759 |
| C | 6.713892 | -1.876811 | -1.220436 | H | -2.59744 | -3.67342 | 2.579094 |
| H | 7.71274 | -1.496681 | -1.412941 | H | -1.22664 | -2.68328 | 2.052389 |
| C | 0.50978 | 3.754908 | -1.003035 | C | 0.071882 | -4.09076 | -0.88969 |
| C | -3.769219 | -0.141507 | 0.978524 | H | -0.95856 | -4.41492 | -0.95524 |
| C | -1.9105 | 4.177927 | -0.639606 | C | 6.130628 | 2.577809 | 2.71444 |
| C | 1.709993 | 4.25803 | -1.528346 | H | 6.308795 | 3.608064 | 3.011844 |
| H | 2.577342 | 3.611328 | -1.594467 | C | -5.95346 | -4.18224 | -1.13356 |
| C | 5.009536 | 2.296667 | 1.897877 | H | -6.72974 | -3.55684 | -0.67285 |
| C | 6.435602 | -3.226102 | -1.403729 | H | -6.0218 | -5.17317 | -0.67156 |
| H | 7.216228 | -3.913411 | -1.718206 | H | -6.19794 | -4.2816 | -2.19612 |
| C | -0.622886 | 4.644229 | -1.016998 | C | -5.67257 | -1.70194 | 2.363633 |
| C | 4.787537 | -5.077319 | -1.480962 | C | -5.37617 | 0.06031 | -0.9986 |
| H | 5.582827 | -5.763717 | -1.759034 | H | -5.90926 | 1.017854 | -0.95964 |
| C | -2.813789 | 0.767757 | 0.318513 | H | -6.02798 | -0.65962 | -1.50191 |
| C | 5.12773 | -3.708269 | -1.228342 | H | -4.49076 | 0.208738 | -1.61926 |
| C | -0.718909 | -1.78304 | -0.356962 | C | -5.93904 | -1.20871 | 1.086642 |
| C | -2.785548 | -2.125152 | -1.71637 | H | -6.88974 | -1.44649 | 0.613273 |
| C | -4.323953 | 2.999006 | 0.25138 | C | 3.496638 | -5.50751 | -1.39918 |
| H | -5.252068 | 2.594368 | 0.644232 | H | 3.244385 | -6.54335 | -1.60963 |
| C | 0.346166 | -2.728843 | -0.582203 | C | 6.955208 | 1.566025 | 3.159224 |
| C | 2.745419 | -3.249668 | -0.771662 | H | 7.808486 | 1.789654 | 3.792828 |
| C | -4.014729 | -2.758736 | -1.912017 | C | -2.90186 | 6.371513 | -0.90005 |
| H | -4.538291 | -2.60825 | -2.853549 | H | -3.75317 | 7.045057 | -0.84256 |
| C | -5.014612 | -0.432655 | 0.383333 | C | -2.2252 | -0.20609 | 3.004017 |
| C | -2.6506 | -3.163877 | 0.487616 | H | -1.33224 | -0.56824 | 2.486994 |
| C | -4.591513 | -3.568786 | -0.932258 | H | -2.21501 | -0.61014 | 4.020657 |
| C | 5.579317 | -0.059488 | 2.003329 | H | -2.12632 | 0.883979 | 3.06692 |
| H | 5.355375 | -1.09409 | 1.781447 | C | 6.65145 | 0.231676 | 2.823248 |
| C | -3.497896 | -0.595172 | 2.290124 | H | 7.255513 | -0.5785 | 3.221303 |
| C | -4.25577 | 4.365648 | -0.07457 | C | -6.65734 | -2.6005 | 3.068925 |
| H | -5.150133 | 4.972615 | 0.041441 | H | -6.57425 | -2.5133 | 4.157383 |
| C | -3.067866 | 4.992622 | -0.532524 | H | -6.47985 | -3.65392 | 2.81213 |
| C | -3.890018 | -3.762679 | 0.258258 | H | -7.68914 | -2.36809 | 2.784491 |

| (*P*^5^*M*^5’^)**-1** (B3LYP/6-31G(d)) | | | | | | | |
| --- | --- | --- | --- | --- | --- | --- | --- |
| C | -1.454539 | 1.548792 | -0.357423 | C | 5.566895 | -3.07581 | -1.00621 |
| C | 2.632474 | -1.096209 | -0.374797 | H | 5.845076 | -4.05351 | -1.39259 |
| C | -2.16372 | 0.377448 | 0.064076 | C | -1.709 | -3.31127 | -0.51303 |
| C | -1.518481 | -0.875131 | -0.128871 | C | -3.68174 | -2.04499 | 0.215696 |
| C | -0.021958 | 1.487629 | -0.371128 | C | -3.47225 | 2.791382 | -1.27206 |
| C | 4.00478 | -1.384797 | -0.41186 | H | -4.07715 | 1.898209 | -1.19442 |
| C | -0.100737 | -0.947963 | -0.319166 | C | 6.583528 | -2.2035 | -0.57442 |
| C | 2.119111 | 0.106806 | 0.045792 | H | 7.615901 | -2.53969 | -0.6391 |
| C | 4.219983 | -2.67762 | -0.914216 | C | 6.325464 | -0.91904 | -0.03119 |
| C | -2.303462 | -2.077796 | -0.138415 | C | 3.7094 | 2.949239 | 2.059102 |
| C | -3.486493 | 0.374565 | 0.689554 | H | 3.386407 | 3.804204 | 2.646785 |
| C | 0.712263 | 2.671443 | -0.727298 | C | 5.069429 | 2.685593 | 1.916074 |
| H | 1.789739 | 2.615499 | -0.788869 | H | 5.805475 | 3.340228 | 2.376896 |
| C | 0.492278 | -2.241341 | -0.652896 | C | 0.092511 | 3.840747 | -1.03633 |
| C | -4.006921 | 1.488542 | 1.368456 | H | 0.681123 | 4.71559 | -1.30207 |
| H | -3.409523 | 2.389134 | 1.44823 | C | 5.494033 | 1.547098 | 1.212155 |
| C | 1.930654 | -2.281895 | -0.816843 | C | -4.45758 | -3.23592 | 0.183589 |
| C | 0.664154 | 0.246602 | -0.156796 | C | 6.884577 | 1.165009 | 1.099993 |
| C | -4.252402 | -0.829183 | 0.698276 | H | 7.625475 | 1.836269 | 1.528034 |
| C | -2.109535 | 2.751635 | -0.87872 | C | -3.85049 | -4.44024 | -0.25441 |
| C | -5.282529 | 1.463567 | 1.934614 | H | -4.45114 | -5.34549 | -0.29665 |
| H | -5.654223 | 2.345357 | 2.449112 | C | -2.51775 | -4.47685 | -0.58899 |
| C | 3.098234 | 1.015114 | 0.68783 | H | -2.05716 | -5.41007 | -0.90352 |
| C | 4.966558 | -0.505875 | 0.033391 | C | -1.94814 | 5.092556 | -1.63314 |
| C | 2.743294 | 2.132063 | 1.464896 | H | -1.32822 | 5.969838 | -1.80281 |
| H | 1.696564 | 2.367469 | 1.617239 | C | -5.814 | -3.19077 | 0.637497 |
| C | -1.329962 | 3.915922 | -1.145963 | H | -6.40616 | -4.10157 | 0.59286 |
| C | -6.078567 | 0.32875 | 1.837426 | C | -3.29471 | 5.117955 | -1.92935 |
| H | -7.083123 | 0.322286 | 2.253176 | H | -3.75867 | 6.022788 | -2.31215 |
| C | 4.509227 | 0.706011 | 0.622609 | C | 7.289828 | -0.00168 | 0.508651 |
| C | -6.34513 | -2.04646 | 1.157292 | H | 8.347631 | -0.25112 | 0.467697 |
| H | -7.36435 | -2.03478 | 1.535614 | C | -4.0515 | 3.940408 | -1.77629 |
| C | 2.883468 | -3.2379 | -1.153886 | H | -5.09791 | 3.925979 | -2.06888 |
| C | -5.577012 | -0.837687 | 1.23308 | H | 0.128672 | -4.30045 | -1.06496 |
| C | -0.316837 | -3.349812 | -0.78171 | H | 2.685392 | -4.24701 | -1.49748 |

| (*M*^5^*M*^5’^)**-1** (B3LYP/6-31G(d)) | | | | | | | |
| --- | --- | --- | --- | --- | --- | --- | --- |
| C | -1.408644 | 1.527961 | 0.171207 | C | 3.944856 | 3.199511 | -1.12219 |
| C | 2.604041 | -1.255366 | 0.195738 | C | 5.278794 | 2.885078 | -0.88 |
| C | -2.216725 | 0.354663 | 0.154873 | C | 0.087379 | 3.737359 | 1.11552 |
| C | -1.573838 | -0.913834 | 0.012697 | C | 5.62062 | 1.60772 | -0.40793 |
| C | -0.005447 | 1.391022 | 0.406138 | C | -4.57718 | -3.15634 | -0.5746 |
| C | 3.965382 | -1.585284 | 0.2843 | C | 6.991027 | 1.174129 | -0.24614 |
| C | -0.140761 | -1.02663 | 0.032068 | C | -3.92154 | -4.3869 | -0.83002 |
| C | 2.132317 | 0.025979 | 0.052828 | C | -2.55651 | -4.48573 | -0.72777 |
| C | 4.124064 | -2.969695 | 0.44993 | C | -1.62144 | 5.31526 | 0.241646 |
| C | -2.377711 | -2.088275 | -0.212954 | C | -6.00415 | -3.0589 | -0.55933 |
| C | -3.667294 | 0.367807 | 0.40994 | C | -2.739 | 5.558834 | -0.53442 |
| C | 0.669375 | 2.514198 | 1.014937 | C | 7.331337 | -0.11144 | 0.079316 |
| C | 0.438698 | -2.370589 | 0.005253 | C | -3.41269 | 4.479305 | -1.13455 |
| C | -4.324475 | 1.426673 | 1.057894 | H | 1.650731 | 2.347751 | 1.440889 |
| C | 1.859811 | -2.491726 | 0.240865 | H | -3.75213 | 2.262573 | 1.43595 |
| C | 0.660822 | 0.153131 | 0.162457 | H | -6.17794 | 2.281841 | 1.724812 |
| C | -4.445405 | -0.784286 | 0.090882 | H | 1.901711 | 2.578351 | -1.06927 |
| C | -1.895464 | 2.894724 | -0.066002 | H | -7.56838 | 0.4018 | 0.874081 |
| C | -5.71027 | 1.435455 | 1.229547 | H | -7.70938 | -1.86143 | -0.10564 |
| C | 3.178303 | 1.031539 | -0.272725 | H | 2.530585 | -4.58863 | 0.459266 |
| C | 4.969618 | -0.65043 | 0.180339 | H | 0.092416 | -4.44932 | -0.29546 |
| C | 2.918975 | 2.293942 | -0.83509 | H | 5.686129 | -4.47019 | 0.715912 |
| C | -1.158551 | 3.996925 | 0.452161 | H | 7.529861 | -2.86538 | 0.565001 |
| C | -6.486377 | 0.381186 | 0.77001 | H | -3.52893 | 2.360992 | -1.37809 |
| C | 4.576051 | 0.680412 | -0.134508 | H | 3.687974 | 4.165183 | -1.549 |
| C | -6.624837 | -1.911491 | -0.16296 | H | 6.061786 | 3.607772 | -1.09749 |
| C | 2.768262 | -3.532282 | 0.403847 | H | 0.596879 | 4.551443 | 1.624775 |
| C | -5.86882 | -0.756482 | 0.222098 | H | 7.773019 | 1.909559 | -0.42053 |
| C | -0.361889 | -3.463266 | -0.236668 | H | -4.52061 | -5.26084 | -1.07352 |
| C | 5.452536 | -3.418045 | 0.57118 | H | -2.05957 | -5.43884 | -0.89033 |
| C | 6.510594 | -2.493885 | 0.486873 | H | -1.05783 | 6.140179 | 0.671222 |
| C | -1.762195 | -3.353667 | -0.403789 | H | -6.58622 | -3.93711 | -0.82773 |
| C | -3.799758 | -2.004749 | -0.268884 | H | -3.07647 | 6.577298 | -0.70597 |
| C | -3.001397 | 3.178442 | -0.900576 | H | 8.378589 | -0.39207 | 0.164711 |
| C | 6.313687 | -1.106185 | 0.271382 | H | -4.25734 | 4.664309 | -1.79254 |

| (*M*^5^*P*^5’^)**-1** (B3LYP/6-31G(d)) | | | | | | | |
| --- | --- | --- | --- | --- | --- | --- | --- |
| C | 1.454573 | 1.548775 | -0.357612 | C | -3.70933 | 2.949 | 2.05952 |
| C | -2.632469 | -1.0962 | -0.374735 | C | -5.06937 | 2.685409 | 1.916418 |
| C | 2.163781 | 0.377399 | 0.063825 | C | -0.09237 | 3.841016 | -1.03562 |
| C | 1.518493 | -0.875151 | -0.129038 | C | -5.49399 | 1.547013 | 1.212363 |
| C | 0.021973 | 1.487689 | -0.371077 | C | 4.457417 | -3.2361 | 0.183943 |
| C | -4.004775 | -1.384769 | -0.411853 | C | -6.88455 | 1.164998 | 1.100065 |
| C | 0.100728 | -0.947932 | -0.31934 | C | 3.850253 | -4.44046 | -0.25379 |
| C | -2.119094 | 0.10679 | 0.045884 | C | 2.517515 | -4.47705 | -0.58841 |
| C | -4.219999 | -2.677452 | -0.914578 | C | 1.948297 | 5.09275 | -1.63265 |
| C | 2.303389 | -2.077877 | -0.138358 | C | 5.81383 | -3.19096 | 0.63787 |
| C | 3.486558 | 0.374563 | 0.689261 | C | 3.294763 | 5.117985 | -1.92929 |
| C | -0.712173 | 2.671678 | -0.726777 | C | -7.28983 | -0.00158 | 0.508511 |
| C | -0.492299 | -2.241313 | -0.652963 | C | 4.051414 | 3.940262 | -1.77681 |
| C | 4.007114 | 1.488644 | 1.367893 | H | -1.78965 | 2.615903 | -0.78816 |
| C | -1.930672 | -2.281827 | -0.816946 | H | 3.409786 | 2.389288 | 1.447521 |
| C | -0.664135 | 0.246629 | -0.156866 | H | 5.654556 | 2.345589 | 2.448202 |
| C | 4.252399 | -0.829246 | 0.698155 | H | -1.69649 | 2.367238 | 1.617563 |
| C | 2.109552 | 2.751609 | -0.878868 | H | 7.083278 | 0.322345 | 2.252671 |
| C | 5.282756 | 1.463695 | 1.933967 | H | 7.364274 | -2.03488 | 1.535708 |
| C | -3.098202 | 1.015013 | 0.688051 | H | -2.68541 | -4.24671 | -1.4982 |
| C | -4.966545 | -0.505854 | 0.033395 | H | -0.12887 | -4.30053 | -1.06458 |
| C | -2.743239 | 2.131871 | 1.465242 | H | -5.84515 | -4.05309 | -1.39347 |
| C | 1.330067 | 3.916087 | -1.14559 | H | -7.61594 | -2.53936 | -0.6398 |
| C | 6.078698 | 0.328798 | 1.836987 | H | 4.076865 | 1.897875 | -1.19552 |
| C | -4.509197 | 0.705941 | 0.622777 | H | -3.38634 | 3.803906 | 2.64728 |
| C | 6.345052 | -2.046585 | 1.157395 | H | -5.80541 | 3.340041 | 2.377248 |
| C | -2.883488 | -3.237695 | -1.154335 | H | -0.68098 | 4.715994 | -1.3009 |
| C | 5.577025 | -0.837734 | 1.232929 | H | -7.62544 | 1.836238 | 1.528154 |
| C | 0.316722 | -3.349877 | -0.781504 | H | 4.450834 | -5.34576 | -0.29583 |
| C | -5.566933 | -3.0755 | -1.006834 | H | 2.056856 | -5.41031 | -0.90272 |
| C | -6.583557 | -2.20324 | -0.574937 | H | 1.328463 | 5.97017 | -1.80192 |
| C | 1.708856 | -3.311394 | -0.512755 | H | 6.405897 | -4.10183 | 0.593425 |
| C | 3.681651 | -2.045085 | 0.215784 | H | 3.758779 | 6.022832 | -2.31198 |
| C | 3.472154 | 2.791215 | -1.272677 | H | -8.34764 | -0.25095 | 0.46742 |
| C | -6.325477 | -0.918905 | -0.0314 | H | 5.097713 | 3.92577 | -2.0698 |

| Compound **2** (B3LYP-D3(BJ)/6-31G(d), Solvent = Toluene, PCM) | | | | | | | |
| --- | --- | --- | --- | --- | --- | --- | --- |
| C | -4.37119 | -1.22842 | -0.10171 | C | 4.194781 | -2.52597 | 0.096475 |
| C | 1.40275 | 3.2124 | 1.19058 | H | 4.840348 | -3.37874 | -0.0826 |
| C | -3.88312 | 0.08683 | 0.12778 | C | -5.87056 | -5.09021 | -1.18323 |
| C | -1.67536 | -0.84664 | 0.62193 | C | 7.057861 | -2.19237 | -0.27679 |
| C | -2.50715 | 0.28781 | 0.41803 | C | 0.112653 | -5.06174 | 2.680694 |
| C | -0.26673 | -0.65853 | 0.77166 | H | 0.697275 | -5.75641 | 3.276659 |
| C | -0.6047 | 1.78394 | 0.86216 | C | 6.646799 | 0.29492 | -0.38008 |
| C | -5.75984 | -1.41148 | -0.37721 | C | 2.871262 | -2.71428 | 0.343893 |
| C | -2.21131 | -2.16752 | 0.5504 | H | 2.469371 | -3.71915 | 0.324709 |
| C | -1.97504 | 1.61349 | 0.57194 | C | -8.04186 | -0.5282 | -0.56943 |
| C | 1.67551 | 0.84665 | 0.62196 | H | -8.71518 | 0.321241 | -0.54032 |
| C | 0.00211 | 3.0262 | 1.33734 | C | 7.281792 | 5.260471 | -1.35257 |
| C | 0.26689 | 0.65852 | 0.77175 | H | 7.652451 | 6.240882 | -1.63945 |
| C | -3.49523 | -2.35994 | -0.00395 | C | -7.65977 | -2.91082 | -0.85718 |
| C | -1.40257 | -3.21247 | 1.19018 | C | -3.59825 | -5.90678 | -1.35849 |
| C | 3.88326 | -0.08674 | 0.12765 | H | -2.89675 | -6.69087 | -1.62779 |
| C | -6.64669 | -0.29475 | -0.37979 | C | -7.42032 | 2.728852 | -1.52749 |
| C | 2.50729 | -0.28776 | 0.41788 | C | 7.561606 | -2.75938 | 0.909132 |
| C | -4.76952 | 1.21217 | 0.01483 | C | -4.96331 | -6.13125 | -1.45455 |
| C | -6.13504 | 1.02069 | -0.20773 | H | -5.34523 | -7.09625 | -1.77659 |
| C | 2.21146 | 2.16755 | 0.55058 | C | 8.142514 | 4.215392 | -1.18281 |
| C | -0.72724 | 3.96339 | 2.10459 | H | 9.211047 | 4.350808 | -1.32776 |
| H | -1.77636 | 3.78339 | 2.30241 | C | -8.43402 | 3.847903 | 0.823709 |
| C | 4.76969 | -1.21203 | 0.01468 | H | -8.83159 | 4.277165 | 1.740976 |
| C | 0.60487 | -1.78397 | 0.86185 | C | 8.041898 | 0.528395 | -0.57012 |
| C | -0.00191 | -3.02631 | 1.33686 | H | 8.715167 | -0.32109 | -0.54111 |
| C | 5.87045 | 5.09041 | -1.18338 | C | 8.795861 | -4.40564 | -0.40558 |
| C | -0.11236 | 5.06138 | 2.68156 | C | -7.56634 | 2.755607 | 0.908683 |
| H | -0.69693 | 5.75593 | 3.27772 | C | -8.80389 | 4.399299 | -0.40609 |
| C | -2.87112 | 2.71436 | 0.34449 | C | -8.52893 | -1.78877 | -0.79046 |
| H | -2.46922 | 3.71923 | 0.3256 | H | -9.59385 | -1.94436 | -0.94209 |
| C | 7.65972 | 2.91099 | -0.85786 | C | -7.28194 | -5.26027 | -1.35211 |
| C | 2.01232 | 4.31356 | 1.82728 | H | -7.65266 | -6.24067 | -1.63898 |
| H | 3.08812 | 4.42235 | 1.77106 | C | -8.14263 | -4.21521 | -1.18208 |
| C | 6.27297 | 2.71899 | -0.63694 | H | -9.2112 | -4.35062 | -1.32678 |

| (*M*^5^P^4^*M*^5^*P*^4^)-**2** (B3LYP/6-31G(d)) | | | | | | | |
| --- | --- | --- | --- | --- | --- | --- | --- |
| C | -4.420785 | 1.007572 | -0.205654 | C | 7.279974 | 3.007471 | -1.61791 |
| C | 2.772162 | 2.147348 | 1.139051 | H | 8.071877 | 3.668081 | -1.9621 |
| C | -3.363365 | 1.927969 | 0.042573 | C | 4.420732 | -1.00753 | -0.20577 |
| C | -1.877262 | 0.05514 | 0.555405 | C | 5.229595 | 1.301255 | -0.64445 |
| C | -2.062469 | 1.449652 | 0.350929 | C | 6.534782 | 0.784607 | -0.91051 |
| C | -0.549205 | -0.45162 | 0.714064 | C | -1.2621 | -3.80531 | 2.095896 |
| C | 0.320611 | 1.857103 | 0.814769 | H | -0.25456 | -4.14407 | 2.304774 |
| C | -5.722313 | 1.51206 | -0.514953 | C | -2.48012 | 4.228085 | 0.050276 |
| C | -2.982283 | -0.849122 | 0.478395 | H | -2.64493 | 5.290177 | -0.11396 |
| C | -0.964639 | 2.363872 | 0.522317 | C | 4.883794 | -3.80492 | -0.32737 |
| C | 1.877214 | -0.055074 | 0.555311 | C | -3.62679 | -3.96228 | 2.531572 |
| C | 1.451671 | 2.649719 | 1.302418 | H | -4.46545 | -4.44509 | 3.025537 |
| C | 0.549167 | 0.451686 | 0.714066 | C | 5.981103 | 3.472011 | -1.48144 |
| C | -4.197264 | -0.407383 | -0.097015 | H | 5.739382 | 4.499153 | -1.74053 |
| C | -2.772155 | -2.147311 | 1.139145 | C | 3.626958 | 3.962097 | 2.531654 |
| C | 3.363314 | -1.927919 | 0.0425 | H | 4.465691 | 4.444846 | 3.025557 |
| C | -5.961269 | 2.918659 | -0.532365 | C | 8.315165 | -2.52752 | -1.01772 |
| C | 2.062423 | -1.449583 | 0.35086 | H | 9.316863 | -2.90554 | -1.20756 |
| C | -3.602265 | 3.341233 | -0.069505 | C | 2.480084 | -4.22805 | 0.050433 |
| C | -4.883845 | 3.804953 | -0.327422 | H | 2.644893 | -5.29016 | -0.11366 |
| C | 2.982227 | 0.849189 | 0.478231 | C | -7.57618 | -1.65572 | -1.35876 |
| C | 1.262209 | 3.805107 | 2.096349 | C | -2.32774 | -4.464 | 2.686602 |
| H | 0.254697 | 4.143749 | 2.305553 | H | -2.1464 | -5.33895 | 3.30487 |
| C | 3.60221 | -3.341194 | -0.069479 | C | 5.961217 | -2.91864 | -0.53235 |
| C | -0.320644 | -1.857041 | 0.814692 | C | 1.229245 | -3.76424 | 0.31288 |
| C | -1.451659 | -2.649714 | 1.302291 | H | 0.399008 | -4.45939 | 0.322425 |
| C | 7.576188 | 1.655705 | -1.358803 | C | -7.28414 | 3.394497 | -0.77307 |
| C | 2.327926 | 4.463717 | 2.687024 | H | -7.45818 | 4.467614 | -0.76305 |
| H | 2.146642 | 5.338505 | 3.305533 | C | 8.888173 | 1.120581 | -1.56727 |
| C | -1.229282 | 3.764285 | 0.312739 | H | 9.680031 | 1.799944 | -1.87304 |
| H | -0.399046 | 4.459432 | 0.32211 | C | -8.0961 | 1.122113 | -1.06343 |
| C | 8.096073 | -1.122123 | -1.063432 | C | -5.98104 | -3.47197 | -1.48142 |
| C | 3.835003 | 2.81475 | 1.785325 | H | -5.73929 | -4.4991 | -1.74053 |
| H | 4.833846 | 2.400934 | 1.716426 | C | -7.27993 | -3.00747 | -1.61788 |
| C | 6.796752 | -0.616479 | -0.800906 | H | -8.07181 | -3.6681 | -1.96207 |

| (*M*^5^*P*^4^*M*^5^*M*^4^)-**2** (B3LYP/6-31G(d)) | | | | | | | |
| --- | --- | --- | --- | --- | --- | --- | --- |
| C | -4.476217 | -0.983963 | 0.047836 | C | 7.09016 | -2.86011 | 2.248683 |
| C | 2.746809 | -2.279373 | -0.916974 | H | 7.833159 | -3.47493 | 2.750588 |
| C | -3.417891 | -1.899007 | -0.201427 | C | 4.497182 | 0.932937 | -0.01943 |
| C | -1.862624 | -0.016726 | -0.478923 | C | 5.167555 | -1.283 | 0.881613 |
| C | -2.090439 | -1.422716 | -0.381432 | C | 6.484416 | -0.77775 | 1.111451 |
| C | -0.522971 | 0.478069 | -0.625899 | C | -1.28561 | 4.222532 | -0.68259 |
| C | 0.292978 | -1.870242 | -0.776155 | H | -0.35225 | 4.679673 | -0.38807 |
| C | -5.808198 | -1.478822 | 0.208195 | C | -2.58098 | -4.21313 | -0.26206 |
| C | -2.962064 | 0.89062 | -0.375499 | H | -2.77406 | -5.27775 | -0.15517 |
| C | -1.009613 | -2.355016 | -0.535913 | C | 5.147921 | 3.627361 | -0.62191 |
| C | 1.896572 | -0.009217 | -0.560915 | C | -3.58776 | 4.481401 | -1.35015 |
| C | 1.420129 | -2.714368 | -1.183326 | H | -4.40868 | 5.107084 | -1.6885 |
| C | 0.54846 | -0.469509 | -0.683628 | C | 5.781905 | -3.30068 | 2.116296 |
| C | -4.212963 | 0.422589 | 0.107486 | H | 5.485146 | -4.25911 | 2.533341 |
| C | -2.716772 | 2.278701 | -0.764147 | C | 3.617428 | -4.21383 | -2.12888 |
| C | 3.499588 | 1.830786 | -0.487661 | H | 4.468767 | -4.77476 | -2.50453 |
| C | -6.079641 | -2.870101 | 0.058588 | C | 8.451007 | 2.386535 | 0.583239 |
| C | 2.157679 | 1.383111 | -0.617574 | H | 9.465687 | 2.74704 | 0.734301 |
| C | -3.694111 | -3.310794 | -0.214094 | C | 2.805246 | 4.02002 | -1.334 |
| C | -5.002549 | -3.762264 | -0.121705 | H | 3.075485 | 4.99444 | -1.73355 |
| C | 2.968037 | -0.936347 | -0.366691 | C | -7.61808 | 1.606107 | 1.363782 |
| C | 1.237889 | -3.876707 | -1.968899 | C | -2.35512 | 5.046159 | -0.98841 |
| H | 0.237629 | -4.154556 | -2.277593 | H | -2.22721 | 6.124994 | -0.96592 |
| C | 3.841974 | 3.189719 | -0.793351 | C | 6.149662 | 2.772481 | -0.11552 |
| C | -0.253656 | 1.888424 | -0.713028 | C | 1.510005 | 3.603479 | -1.3757 |
| C | -1.395603 | 2.806064 | -0.716419 | H | 0.781931 | 4.239017 | -1.85881 |
| C | 7.461089 | -1.589989 | 1.76723 | C | -7.43022 | -3.32354 | 0.127954 |
| C | 2.310736 | -4.626112 | -2.421722 | H | -7.62953 | -4.38252 | -0.01635 |
| H | 2.135335 | -5.505282 | -3.035642 | C | 8.788091 | -1.07878 | 1.938261 |
| C | -1.307343 | -3.756902 | -0.382391 | H | 9.528931 | -1.71576 | 2.415203 |
| H | -0.487867 | -4.462068 | -0.334537 | C | -8.20638 | -1.07221 | 0.617129 |
| C | 8.144522 | 1.043155 | 0.939577 | C | -5.9631 | 3.279645 | 1.924184 |
| C | 3.824227 | -3.04957 | -1.410341 | H | -5.69883 | 4.226652 | 2.386679 |
| H | 4.836704 | -2.697839 | -1.256494 | C | -7.28868 | 2.875065 | 1.876354 |
| C | 6.827514 | 0.557496 | 0.732095 | H | -8.07694 | 3.508884 | 2.275016 |

| (*M*^5^*P*^4^*P*^5^*M*^4^)-**2** (B3LYP/6-31G(d)) | | | | | | | |
| --- | --- | --- | --- | --- | --- | --- | --- |
| C | 4.536147 | -0.946003 | -0.183754 | C | -7.56039 | -2.87803 | -1.38094 |
| C | -2.648193 | -2.278235 | 0.731271 | H | -8.39491 | -3.50156 | -1.69202 |
| C | 3.459917 | -1.856412 | -0.367009 | C | -4.53614 | 0.946004 | 0.183764 |
| C | 1.875904 | 0.00489 | -0.206971 | C | -5.38437 | -1.28141 | -0.50294 |
| C | 2.123362 | -1.395557 | -0.240513 | C | -6.72185 | -0.78358 | -0.42992 |
| C | 0.524992 | 0.471736 | -0.11842 | C | 0.993662 | 3.949621 | -1.37453 |
| C | -0.248534 | -1.871001 | 0.178835 | H | -0.04029 | 4.260565 | -1.45454 |
| C | 5.877535 | -1.434816 | -0.242224 | C | 2.603418 | -4.12922 | -0.75413 |
| C | 2.967289 | 0.927924 | -0.250736 | H | 2.788049 | -5.16109 | -1.04258 |
| C | 1.041956 | -2.33313 | -0.173046 | C | -5.03837 | 3.680708 | 0.74614 |
| C | -1.875902 | -0.004894 | 0.20694 | C | 3.293447 | 4.254858 | -2.01754 |
| C | -1.301478 | -2.736439 | 0.711104 | H | 4.060782 | 4.817516 | -2.54208 |
| C | -0.524991 | -0.471738 | 0.118384 | C | -6.25346 | -3.30684 | -1.55904 |
| C | 4.278038 | 0.450378 | 0.012507 | H | -6.05431 | -4.26425 | -2.03265 |
| C | 2.648192 | 2.278227 | -0.731322 | C | -3.29345 | -4.25489 | 2.017455 |
| C | -3.45991 | 1.856408 | 0.367024 | H | -4.06078 | -4.81756 | 2.541981 |
| C | 6.127432 | -2.80263 | -0.562811 | C | -8.52704 | 2.397342 | 0.451836 |
| C | -2.123358 | 1.395553 | 0.240503 | H | -9.55045 | 2.755917 | 0.532108 |
| C | 3.726127 | -3.244811 | -0.621738 | C | -2.6034 | 4.129206 | 0.754198 |
| C | 5.038385 | -3.680719 | -0.746062 | H | -2.78803 | 5.161063 | 1.042682 |
| C | -2.967289 | -0.927926 | 0.250701 | C | 7.818687 | 1.606814 | 0.833639 |
| C | -0.993661 | -3.949644 | 1.37444 | C | 1.967707 | 4.709091 | -1.99781 |
| H | 0.040293 | -4.260593 | 1.454429 | H | 1.69225 | 5.627039 | -2.50982 |
| C | -3.726115 | 3.244801 | 0.621791 | C | -6.12742 | 2.802625 | 0.562882 |
| C | 0.248535 | 1.870999 | -0.17887 | C | -1.33365 | 3.701676 | 0.514758 |
| C | 1.301477 | 2.736432 | -0.711158 | H | -0.5082 | 4.393271 | 0.635767 |
| C | -7.818701 | -1.606788 | -0.833637 | C | 7.477697 | -3.24815 | -0.67805 |
| C | -1.967706 | -4.709127 | 1.99771 | H | 7.659693 | -4.2866 | -0.94363 |
| H | -1.69225 | -5.627088 | 2.509693 | C | -9.15234 | -1.10234 | -0.69631 |
| C | 1.333659 | -3.701691 | -0.514714 | H | -9.98002 | -1.74917 | -0.97672 |
| H | 0.508212 | -4.393294 | -0.635697 | C | 8.306522 | -1.04119 | -0.08069 |
| C | -8.30652 | 1.0412 | 0.080753 | C | 6.253437 | 3.306878 | 1.558984 |
| C | -3.614332 | -3.052792 | 1.413841 | H | 6.054276 | 4.2643 | 2.032569 |
| H | -4.62648 | -2.677838 | 1.495454 | C | 7.56037 | 2.87807 | 1.380915 |
| C | -6.975191 | 0.558746 | -0.008281 | H | 8.394881 | 3.501609 | 1.691989 |

| Compound **3** (B3LYP/6-31G(d)), Solvent = Toluene, PCM) | | | | | | | |
| --- | --- | --- | --- | --- | --- | --- | --- |
| C | -5.4476 | -6.12556 | -0.70576 | C | -0.12651 | 5.379343 | -1.72798 |
| C | -6.27097 | -5.01443 | -0.39827 | C | 1.220699 | 5.586122 | -1.48153 |
| C | -5.66906 | -3.73078 | -0.29452 | C | 6.971748 | -2.30519 | 0.802436 |
| C | -4.26245 | -3.58045 | -0.49184 | C | 7.498512 | -3.09199 | -0.23889 |
| C | -3.46673 | -4.70601 | -0.81638 | C | 8.292868 | -4.1967 | 0.080443 |
| C | -4.09864 | -5.97418 | -0.91131 | C | 8.577627 | -4.53915 | 1.405158 |
| C | -7.67296 | -5.14731 | -0.19115 | C | 8.039315 | -3.74699 | 2.423197 |
| C | -8.44851 | -4.05599 | 0.099821 | C | 7.242241 | -2.63341 | 2.14447 |
| C | -7.87751 | -2.75756 | 0.202047 | C | 6.672911 | -1.79847 | 3.265912 |
| C | -6.48041 | -2.59534 | 0.005286 | C | 7.203727 | -2.75149 | -1.67976 |
| C | -8.66891 | -1.60983 | 0.492499 | C | 9.463848 | -5.71771 | 1.727759 |
| C | -8.10941 | -0.3651 | 0.581534 | C | -6.9717 | 2.305131 | 0.802752 |
| C | -6.70683 | -0.16598 | 0.389519 | C | -7.49833 | 3.092135 | -0.23884 |
| C | -5.89058 | -1.29868 | 0.097455 | C | -8.29263 | 4.196638 | 0.080257 |
| C | -2.04156 | -4.53326 | -1.03309 | C | -8.57776 | 4.539056 | 1.405173 |
| C | -1.44924 | -3.25376 | -0.81164 | C | -8.0398 | 3.746953 | 2.423091 |
| C | -0.03431 | -3.08672 | -0.92632 | C | -7.24248 | 2.633204 | 2.144496 |
| C | 0.716409 | -4.14543 | -1.45549 | C | -6.67357 | 1.79831 | 3.266189 |
| C | 0.126463 | -5.37924 | -1.72839 | C | -7.20317 | 2.751473 | -1.6796 |
| C | -1.22073 | -5.58604 | -1.48188 | C | -9.46407 | 5.717936 | 1.726366 |
| C | -2.27687 | -2.11959 | -0.52793 | H | -5.90139 | -7.11036 | -0.77712 |
| C | -3.66995 | -2.27864 | -0.37804 | H | -3.50812 | -6.85447 | -1.13422 |
| C | -4.48997 | -1.1417 | -0.10024 | H | -8.11878 | -6.13513 | -0.27037 |
| C | -3.91297 | 0.152334 | -0.02963 | H | -9.51844 | -4.16682 | 0.254715 |
| C | -2.51369 | 0.323669 | -0.25345 | H | -9.73764 | -1.74035 | 0.641079 |
| C | -1.69458 | -0.81584 | -0.47043 | H | -8.72739 | 0.498729 | 0.799993 |
| C | -0.28034 | -0.65611 | -0.53757 | H | 1.761699 | -3.99744 | -1.69336 |
| C | 0.28036 | 0.656123 | -0.53753 | H | 0.730171 | -6.18219 | -2.14077 |
| C | 1.694608 | 0.815852 | -0.47046 | H | -1.64617 | -6.5606 | -1.68736 |
| C | 2.513729 | -0.32368 | -0.25362 | H | 4.704312 | -3.40154 | 0.741208 |
| C | 1.939482 | -1.63951 | -0.26619 | H | 2.33713 | -3.70155 | 0.302141 |
| C | 0.567445 | -1.80153 | -0.57821 | H | -4.70422 | 3.401448 | 0.741747 |
| C | 3.913017 | -0.15236 | -0.02983 | H | -2.33705 | 3.701496 | 0.302582 |
| C | 4.743412 | -1.28137 | 0.275856 | H | 9.737694 | 1.740307 | 0.640897 |
| C | 4.107268 | -2.5616 | 0.403776 | H | 8.727453 | -0.49879 | 0.799691 |

b. Optimized structures of the transition states with one imaginary frequency

| (*1-TS^5^*)-**1** (B3LYP/6-31G(d)) | | | | | | | |
| --- | --- | --- | --- | --- | --- | --- | --- |
| C | -1.364956 | 1.53495 | 0.014249 | C | 5.153639 | -3.46255 | -1.05418 |
| C | 2.548096 | -1.285156 | 0.197574 | H | 5.275633 | -4.4874 | -1.3964 |
| C | -2.234723 | 0.422757 | 0.120383 | C | -1.90565 | -3.32197 | 0.136567 |
| C | -1.630817 | -0.859472 | 0.24877 | C | -3.89786 | -1.90338 | 0.108688 |
| C | -0.056041 | 1.426525 | 0.585265 | C | -2.60098 | 2.881429 | -1.71739 |
| C | 3.8642 | -1.652933 | -0.165514 | H | -3.06306 | 1.972098 | -2.08651 |
| C | -0.195149 | -0.988596 | 0.330899 | C | 6.252078 | -2.59118 | -1.09682 |
| C | 2.154574 | -0.017906 | 0.591485 | H | 7.206171 | -2.96676 | -1.4595 |
| C | 3.915321 | -2.993254 | -0.583829 | C | 6.171214 | -1.23774 | -0.68971 |
| C | -2.476781 | -2.023594 | 0.175361 | C | 4.440072 | 3.003213 | 1.514933 |
| C | -3.694433 | 0.554776 | 0.236075 | H | 4.337316 | 3.936786 | 2.061464 |
| C | 0.358562 | 2.662762 | 1.21541 | C | 5.641415 | 2.680396 | 0.908333 |
| H | 0.962094 | 2.581063 | 2.101196 | H | 6.488523 | 3.361041 | 0.94331 |
| C | 0.329091 | -2.334553 | 0.166912 | C | -0.13688 | 3.883282 | 0.883536 |
| C | -4.333062 | 1.772183 | 0.527193 | H | 0.178534 | 4.770241 | 1.42693 |
| H | -3.743986 | 2.662731 | 0.695631 | C | 5.77598 | 1.42142 | 0.306656 |
| C | 1.742835 | -2.468353 | -0.017903 | C | -4.71432 | -3.06719 | 0.036091 |
| C | 0.64292 | 0.167969 | 0.589235 | C | 7.044859 | 0.973106 | -0.21809 |
| C | -4.50711 | -0.615126 | 0.174829 | H | 7.868054 | 1.683504 | -0.2025 |
| C | -1.719163 | 2.811692 | -0.616021 | C | -4.10347 | -4.34661 | 0.003729 |
| C | -5.722888 | 1.868871 | 0.615964 | H | -4.73604 | -5.22878 | -0.05569 |
| H | -6.174737 | 2.833017 | 0.831857 | C | -2.73913 | -4.46959 | 0.052569 |
| C | 3.332673 | 0.920263 | 0.760582 | H | -2.27059 | -5.44993 | 0.019237 |
| C | 4.912929 | -0.767469 | -0.213925 | C | -1.37852 | 5.230395 | -0.8237 |
| C | 3.340011 | 2.141635 | 1.453168 | H | -0.88716 | 6.135848 | -0.47562 |
| H | 2.484474 | 2.446743 | 2.013034 | C | -6.13688 | -2.92542 | 0.023634 |
| C | -1.071871 | 4.004695 | -0.19754 | H | -6.74496 | -3.82393 | -0.04718 |
| C | -6.522036 | 0.749761 | 0.435517 | C | -2.26669 | 5.276197 | -1.88421 |
| H | -7.605342 | 0.828001 | 0.483502 | H | -2.48671 | 6.22129 | -2.37299 |
| C | 4.651727 | 0.543347 | 0.264274 | C | 7.245827 | -0.29104 | -0.69629 |
| C | -6.723217 | -1.699451 | 0.130781 | H | 8.2234 | -0.58977 | -1.06733 |
| H | -7.805922 | -1.603641 | 0.152018 | C | -2.86509 | 4.088806 | -2.34318 |
| C | 2.555645 | -3.500288 | -0.460773 | H | -3.53408 | 4.114539 | -3.19893 |
| C | -5.931905 | -0.510589 | 0.237971 | H | -0.05839 | -4.42171 | 0.036029 |
| C | -0.501757 | -3.432848 | 0.120028 | H | 2.233078 | -4.50016 | -0.72715 |

| (*2-TS^5'^*)-**1** (B3LYP/6-31G(d)) | | | | | | | |
| --- | --- | --- | --- | --- | --- | --- | --- |
| C | 1.559528 | 1.554274 | -0.577268 | C | -3.65344 | 2.603 | 2.424948 |
| C | -2.617624 | -0.984034 | -0.62669 | C | -5.01426 | 2.445389 | 2.174493 |
| C | 2.295691 | 0.299662 | -0.511581 | C | -0.03851 | 3.895463 | 0.040964 |
| C | 1.536755 | -0.910951 | -0.353015 | C | -5.44795 | 1.481864 | 1.250505 |
| C | 0.122316 | 1.492164 | -0.357206 | C | 4.020498 | -3.28407 | 1.216271 |
| C | -3.996815 | -1.218587 | -0.749718 | C | -6.84606 | 1.17837 | 1.03418 |
| C | 0.114072 | -0.940499 | -0.483007 | C | 3.25936 | -4.48054 | 1.229896 |
| C | -2.076074 | 0.144045 | -0.061252 | C | 2.029652 | -4.53285 | 0.621937 |
| C | -4.230523 | -2.45736 | -1.366521 | C | 1.947788 | 5.313495 | 0.025987 |
| C | 2.161704 | -2.145746 | 0.060265 | C | 5.346576 | -3.226 | 1.743244 |
| C | 3.773524 | 0.099883 | -0.444399 | C | 3.206754 | 5.598066 | -0.46669 |
| C | -0.630762 | 2.710087 | -0.219459 | C | -7.26854 | 0.130861 | 0.261051 |
| C | -0.539333 | -2.222212 | -0.739138 | C | 3.847708 | 4.608836 | -1.21946 |
| C | 4.75824 | 0.792349 | -1.160538 | H | -1.70514 | 2.658381 | -0.27531 |
| C | -1.941399 | -2.173867 | -1.097507 | H | 4.489514 | 1.324775 | -2.05201 |
| C | -0.604057 | 0.268695 | -0.283421 | H | 6.824646 | 1.324274 | -1.42017 |
| C | 4.290997 | -0.984304 | 0.347794 | H | -1.64831 | 2.021649 | 1.957002 |
| C | 2.116675 | 2.933399 | -0.66912 | H | 7.623222 | -0.15027 | 0.430216 |
| C | 6.124101 | 0.719462 | -0.851068 | H | 7.174605 | -2.13047 | 1.834921 |
| C | -3.051845 | 0.958258 | 0.709552 | H | -2.72025 | -4.05635 | -1.95037 |
| C | -4.947375 | -0.36741 | -0.233984 | H | -0.37066 | -4.34483 | -0.61851 |
| C | -2.694333 | 1.874619 | 1.716245 | H | -5.8741 | -3.72963 | -2.02925 |
| C | 1.370746 | 4.034794 | -0.127251 | H | -7.62802 | -2.23799 | -1.19415 |
| C | 6.572996 | -0.115458 | 0.15239 | H | 3.760585 | 2.680205 | -2.03292 |
| C | -4.4704 | 0.722608 | 0.547277 | H | -3.32402 | 3.302302 | 3.188589 |
| C | 6.141619 | -2.145272 | 1.496149 | H | -5.74517 | 3.032705 | 2.725292 |
| C | -2.902345 | -3.060759 | -1.561659 | H | -0.63131 | 4.782219 | 0.251301 |
| C | 5.673128 | -1.048517 | 0.702478 | H | -7.5784 | 1.80119 | 1.542619 |
| C | 0.137922 | -3.392191 | -0.495111 | H | 3.682326 | -5.36718 | 1.69559 |
| C | -5.582717 | -2.799869 | -1.546427 | H | 1.46386 | -5.46087 | 0.602556 |
| C | -6.589667 | -1.940821 | -1.065663 | H | 1.345971 | 6.091862 | 0.489052 |
| C | 1.4471 | -3.371325 | 0.047874 | H | 5.731166 | -4.08415 | 2.288748 |
| C | 3.478266 | -2.128778 | 0.588711 | H | 3.643436 | 6.585864 | -0.35003 |
| C | 3.299764 | 3.337555 | -1.329052 | H | -8.33144 | -0.07422 | 0.156459 |
| C | -6.314346 | -0.73299 | -0.377013 | H | 4.766832 | 4.833772 | -1.75366 |

| (*1-TS^4^*)-**2** (B3LYP/6-31G(d)) | | | | | | | |
| --- | --- | --- | --- | --- | --- | --- | --- |
| C | -4.369923 | 1.018477 | -0.132473 | C | 6.929056 | 3.042456 | -2.071252 |
| C | 2.779163 | 2.137262 | 1.229149 | H | 7.648538 | 3.708121 | -2.541574 |
| C | -3.319688 | 1.913742 | 0.210455 | C | 4.418405 | -0.983484 | -0.118724 |
| C | -1.842599 | 0.003369 | 0.659579 | C | 5.07361 | 1.324638 | -0.782104 |
| C | -2.028688 | 1.411018 | 0.525451 | C | 6.360503 | 0.838723 | -1.166798 |
| C | -0.530758 | -0.544684 | 0.865469 | C | -1.524186 | -4.187601 | 1.520559 |
| C | 0.328042 | 1.786197 | 1.059498 | H | -0.63981 | -4.789463 | 1.453731 |
| C | -5.659219 | 1.542263 | -0.459882 | C | -2.441634 | 4.209803 | 0.378692 |
| C | -2.955936 | -0.86695 | 0.505733 | H | -2.602442 | 5.280049 | 0.274314 |
| C | -0.944175 | 2.315428 | 0.77471 | C | 5.058874 | -3.737809 | 0.069795 |
| C | 1.889368 | -0.089485 | 0.731147 | C | -3.880394 | -4.162973 | 2.044692 |
| C | 1.465301 | 2.584719 | 1.521296 | H | -4.766158 | -4.64394 | 2.449387 |
| C | 0.556433 | 0.381444 | 0.924378 | C | 5.641144 | 3.475141 | -1.795933 |
| C | -4.147339 | -0.395534 | -0.101854 | H | 5.334054 | 4.481013 | -2.068809 |
| C | -2.796706 | -2.205317 | 1.064713 | C | 3.691903 | 3.958805 | 2.571034 |
| C | 3.438721 | -1.919834 | 0.318008 | H | 4.554004 | 4.470756 | 2.989588 |
| C | -5.901409 | 2.945562 | -0.390135 | C | 8.289275 | -2.391842 | -1.206189 |
| C | 2.118265 | -1.483982 | 0.631448 | H | 9.282524 | -2.740187 | -1.478958 |
| C | -3.558435 | 3.331935 | 0.178884 | C | 2.78469 | -4.190455 | 0.920642 |
| C | -4.831243 | 3.813598 | -0.088645 | H | 3.059618 | -5.221398 | 1.129927 |
| C | 2.961063 | 0.844341 | 0.558782 | C | -7.453216 | -1.55895 | -1.613196 |
| C | 1.303193 | 3.704456 | 2.368576 | C | -2.665671 | -4.848306 | 1.942547 |
| H | 0.305721 | 3.994244 | 2.677698 | H | -2.596746 | -5.897434 | 2.217108 |
| C | 3.784602 | -3.307283 | 0.40947 | C | 6.037286 | -2.837503 | -0.395978 |
| C | -0.299335 | -1.96906 | 0.966696 | C | 1.515613 | -3.771157 | 1.16921 |
| C | -1.507032 | -2.809843 | 1.168106 | H | 0.867628 | -4.487568 | 1.63457 |
| C | 7.306202 | 1.717197 | -1.781525 | C | -7.215782 | 3.435137 | -0.647449 |
| C | 2.394131 | 4.392166 | 2.875259 | H | -7.395184 | 4.504589 | -0.569447 |
| H | 2.239086 | 5.239338 | 3.53773 | C | 8.606844 | 1.217277 | -2.111443 |
| C | -1.198632 | 3.727451 | 0.638128 | H | 9.326424 | 1.902077 | -2.553351 |
| H | -0.367707 | 4.417581 | 0.706088 | C | -8.005641 | 1.187116 | -1.130765 |
| C | 7.98635 | -1.009156 | -1.355645 | C | -5.837709 | -3.349968 | -1.810439 |
| C | 3.874826 | 2.839167 | 1.77741 | H | -5.575219 | -4.35097 | -2.141599 |
| H | 4.878445 | 2.471862 | 1.598725 | C | -7.134293 | -2.883821 | -1.965755 |
| C | 6.698442 | -0.539113 | -0.989705 | H | -7.904213 | -3.51918 | -2.396508 |

| (*2-TS^5^*)-**2** (B3LYP/6-31G(d)) | | | | | | | |
| --- | --- | --- | --- | --- | --- | --- | --- |
| C | 4.512957 | -0.957088 | -0.334834 | C | -7.66258 | -2.30814 | -1.85642 |
| C | -2.647892 | -2.393601 | 0.170594 | H | -8.53327 | -2.80761 | -2.27403 |
| C | 3.466707 | -1.885894 | -0.545899 | C | -4.42763 | 0.910543 | 0.460144 |
| C | 1.905082 | -0.010675 | -0.632294 | C | -5.39142 | -1.04866 | -0.71321 |
| C | 2.138371 | -1.411367 | -0.684429 | C | -6.70305 | -0.54004 | -0.46102 |
| C | 0.568428 | 0.41481 | -0.330281 | C | 0.8526 | 4.079406 | -0.85005 |
| C | -0.236434 | -1.94538 | -0.328376 | H | -0.14748 | 4.375676 | -0.56637 |
| C | 5.699352 | -1.439341 | 0.301274 | C | 2.679613 | -4.18095 | -0.90303 |
| C | 3.000141 | 0.925545 | -0.829055 | H | 2.918792 | -5.2228 | -1.10107 |
| C | 1.060749 | -2.361675 | -0.692036 | C | -4.78809 | 3.421618 | 1.731828 |
| C | -1.811009 | -0.086071 | 0.094952 | C | 2.838633 | 4.555603 | -2.0813 |
| C | -1.307255 | -2.865621 | 0.079308 | H | 3.438964 | 5.209718 | -2.70773 |
| C | -0.481685 | -0.549377 | -0.168262 | C | -6.38218 | -2.72723 | -2.18696 |
| C | 4.342688 | 0.435404 | -0.688363 | H | -6.24137 | -3.54808 | -2.88481 |
| C | 2.550335 | 2.319727 | -1.077213 | C | -3.34811 | -4.55481 | 1.08008 |
| C | -3.30976 | 1.713385 | 0.81118 | H | -4.1354 | -5.18762 | 1.480441 |
| C | 5.874553 | -2.828559 | 0.564908 | C | -8.33829 | 2.369858 | 1.27495 |
| C | -2.005046 | 1.265817 | 0.484091 | H | -9.34176 | 2.729369 | 1.48994 |
| C | 3.726933 | -3.297701 | -0.465541 | C | -2.33241 | 3.775309 | 1.724377 |
| C | 4.924567 | -3.74525 | 0.066125 | H | -2.45119 | 4.693629 | 2.294065 |
| C | -2.932683 | -0.964957 | -0.012132 | C | 7.790634 | 1.651412 | 0.445045 |
| C | -1.031225 | -4.171104 | 0.555003 | C | 1.631144 | 4.990265 | -1.54247 |
| H | -0.005489 | -4.505974 | 0.619553 | H | 1.263525 | 5.99651 | -1.72341 |
| C | -3.50203 | 2.994222 | 1.430861 | C | -5.92066 | 2.655527 | 1.378967 |
| C | 0.317816 | 1.792185 | -0.058258 | C | -1.10044 | 3.395164 | 1.286095 |
| C | 1.274265 | 2.754254 | -0.591864 | H | -0.23636 | 4.005249 | 1.524894 |
| C | -7.847308 | -1.201571 | -1.005582 | C | 6.989853 | -3.24819 | 1.350197 |
| C | -2.025757 | -5.011047 | 1.024526 | H | 7.105959 | -4.30871 | 1.559434 |
| H | -1.767146 | -6.00074 | 1.391061 | C | -9.15264 | -0.70283 | -0.6896 |
| C | 1.406115 | -3.725045 | -1.023625 | H | -10.0164 | -1.23135 | -1.08533 |
| H | 0.621534 | -4.395453 | -1.35396 | C | 7.771321 | -0.95461 | 1.545844 |
| C | -8.19011 | 1.14766 | 0.561202 | C | 7.158684 | 2.969528 | -1.45995 |
| C | -3.638333 | -3.261954 | 0.686055 | H | 7.381514 | 3.737692 | -2.19533 |
| H | -4.645881 | -2.890771 | 0.817499 | C | 7.977474 | 2.792137 | -0.36348 |
| C | -6.884671 | 0.661081 | 0.293003 | H | 8.828862 | 3.443967 | -0.18539 |
